# Supplementary material for: Demographic, behavioural and occupational risk factors associated with SARS-CoV-2 infection in UK healthcare workers: a retrospective observational study
Source: BMJ Open. 2022 Nov 7;12(11):e063159. doi: 10.1136/bmjopen-2022-063159 (PMC9644078; doi:10.1136/bmjopen-2022-063159)
Supplement: Supplementary data [file bmjopen-2022-063159supp001.pdf]

## A. Demographics

1. How many people live in your household (select number)
2. Do you rent a room in a shared house (yes/no)
3. Do you live with other healthcare workers (yes/no)
4. Do you live with other key workers (who are not healthcare workers) who have worked during this time? (yes/no)
5. Is there more than one generation of your family living in your household (e.g. Children, parents or grandparents) (yes/no) If yes (may be multiple):

- Children
- Parents
- Grandparents
- More than one of the above
- Other (free text)

6. Are there children living in your house? (yes/no)

- If yes, how many.
- What ages (select from drop down list for each child – multiple depending on how many entered in answer above if possibly [– *on REDCap, or provide boxes for paper form*])

7. Do you have school aged children? (yes/no). If yes:

- Did they attend school between March to May 2020? (yes/no)
- Did they attend school between from June to July 2020? (yes/no)

8. Do you have children who attend nursery. (yes/no). If yes:

- Did they attend nursery between March to May 2020? (yes/no)
- Did they attend nursery between from June to July 2020? (yes/no)

9. Is there anyone in your household who is >65 years old? (yes/no)
10. Did anybody in your household (other than yourself) test positive on a throat swab (PCR test) for COVID-19?
11. Did anybody in your household (other than yourself) test positive on a blood test (antibody test) for COVID-19?
12. Did anybody (other than yourself) have symptoms consistent with COVID-19 between February and July 2020?
13. How do you travel to and from work? (drop down list)

- Walk/run; cycle; personal car; bus; train

14. If you drive, do you share lifts with other healthcare workers who aren't in your immediate household? (yes/no)

## B. Socioeconomic

1. Were you born in the United Kingdom? (yes/no)

2. Ethnicity (drop down list) *[– insert List of NHS ethnicity codes A-Z ]*
3. What is your highest level of education? (Select from list: GCSE; A level; Undergraduate degree; higher degree; other vocational training)
4. Have you been employed in more than one job during this time? (yes/no)
5. Do you have any dependents other than your immediate family members? (yes/no)
6. Do you provide care for anyone outside of your immediate household? (including washing/dressing, cooking, shopping, cleaning, healthcare needs) (yes/no)

**C. Occupational**

1. What is your job role?

- Admin or reception staff
- Staff nurse
- Nursing Sister / Senior nursing staff
- Consultant
- Junior doctor (including FY1/FY2/Core trainee/Speciality Trainee)
- Laboratory staff
- Healthcare Assistant
- Operating department staff
- Manager
- Radiographer
- Midwife
- Physiotherapist / Physiotherapy assistant
- Pharmacy staff
- Cleaning/domestic staff
- Dietician
- Occupational therapist
- SALT
- Porter
- Other (FREE TEXT)

2. Please select all areas you have worked during this time (may be multiple):

- Ward A2 - Neurosciences critical care unit (NCCU)
- Ward J2 - Trauma high dependency unit
- Ward A4 - Neurology / Neurosurgery
- Ward A5 - Neuro-oncology / Neurosurgery
- Ward C2 - Children's oncology and haematology
- Ward C3 - Children’s surgical and medicine
- Ward C4 - Frail and Acute Medicine for the Elderly
- Ward C5 - General medicine and nephrology
- Ward C6 - Medicine for the elderly
- Ward C7 - Gastroenterology
- Ward C8 - Surgical Admissions for ‘Amber’ patients

- Ward C9 - Teenage Cancer Trust Unit
- Ward C10 - Haematology and haematological oncology
- Ward D2 - Children's surgical and medicine
- Ward D3 - John Farman intensive care unit
- Ward D4 - Intermediate dependancy area
- Ward D5 – DME Medicine for the elderly
- Ward D6 - Neuro/Stroke/ Neurosurgery/Gastro  Haematology
- Ward D7 - Diabetes and endocrinology
- Ward D9 - Oncology
- Ward D10 - Respiratory
- Ward EAU 2 - Paediatric Emergency Department
- Ward EAU 3 - Ambulatory care
- Ward EAU 4 - Acute Hub - Green Medical Admissions/Short Stay
- Ward EAU 5 - Acute Hub - Red Medicine
- Ward F2 - Inpatient Occupational Therapy
- Ward F3
- Ward F4 - Renal
- Ward F5 - Transplant high dependency unit
- Ward F6 - Trauma and Orthopaedics
- Ward G2 - Infusion services
- Ward G3 - Diabetes, I.D. and Oncology
- Ward G4 - Hepatology
- Ward G5 - Transplant unit
- Ward G6 - Medicine for the elderly
- Ward J2 - Major trauma unit
- Ward J3 - Post Anaesthetic Care Unit (PACU) and 23 Hour Stays
- Ward K2 - Cardiology
- Ward K3 - Cardiology and coronary care unit
- Ward L2 - Day surgery unit
- Ward L4 - Non-Elective Surgery Patients
- Ward L5 - Non-Elective Surgery Patients
- Ward M4 - Non-Elective Surgery Patients
- Ward M5 - Elective Surgery Patients
- Ward N2 -Amber Medical Admissions for Covid Pathway
- Ward N3 - Respiratory medicine
- Ward R3 - Neurosciences
- Ward S3 - Psychiatry
- Surgical Ambulatory Care Unit
- Clinical Investigation Ward (CIW)
- Clinical Research Facility (CRF)
- Coronary care unit (CCU)
- Haematology day unit
- Intermediate dependency area (IDA)
- Ward EAU 4 - Acute Hub - Green Medical Admissions/Short Stay
- Paediatric intensive care unit (PICU)
- Paediatric Day Unit (PDU)
- Stroke Unit - Ward R2 and Lewin rehabilitation unit
- Delivery unit

- Ward - Lady Mary - Postnatal
- Neonatal unit
- Ward - Sara - Antenatal
- Daphne ward – Gynaecology
- Ward - Charles Wolfson

3. Have you been involved in the direct patient care of patients with confirmed COVID-19? (yes/no)

4. Have you worked in a specified “Red” area between March and July 2020? (yes/no). If yes:

- Less than 1 week
- 1 week
- 1 week – 1 month
- >1 month

5. Which speciality have you predominantly worked in between March and July 2020?

- Emergency Department
- Critical Care
- Acute Medicine
- Respiratory Medicine
- Infectious Diseases
- Medicine (not including Respiratory or Infectious Diseases)
- Operating Department (Theatres)
- ENT
- Surgical specialties
- Paediatrics
- Research
- Non-patient facing role

6. How many hours did you work in the average week from March to May 2020?

7. How many hours did you work in the average week from June to July 2020?

8. Does your working pattern include night shifts? (yes/no)

9. Have you been present during aerosol generating procedures on COVID-19 confirmed patients? (yes/no). If yes:

- tracheal intubation and extubation
- manual ventilation
- tracheotomy or tracheostomy procedures (insertion or removal)
- bronchoscopy
- dental procedures (using high speed devices, for example ultrasonic scalers/high speed drills
- non-invasive ventilation (NIV); Bi-level Positive Airway Pressure Ventilation (BiPAP) and Continuous Positive Airway Pressure Ventilation (CPAP)
- high flow nasal oxygen (HFNO)
- high frequency oscillatory ventilation (HFOV)
- induction of sputum using nebulised saline

- respiratory tract suctioning
- upper ENT airway procedures that involve respiratory suctioning
- upper gastro-intestinal endoscopy where open suction of the upper respiratory tract occurs
-

10. Did you receive formal PPE training? (yes/no)

11. Did you feel that adequate PPE was available to you:

- At all times
- Most of the time
- Some of the time
- Rarely

12. Prior to the introduction of hospital-wide surgical-resistant masks, which type of facemask did you predominantly use at work?

- None
- Water resistant surgical mask
- FFP3
- Respirator hood
- Other respirator
- Other

13. After the introduction of hospital-wide surgical-resistant masks, which type of facemask did you predominantly use at work?

- None
- Water resistant surgical mask
- FFP3
- Respirator hood
- Other respirator
- Other

14. What type of eye protection did you predominantly use at work:

- None
- Own spectacles/glasses
- Protective glasses (hospital supplied)
- Goggles
- Face shield

15. Did you take rest/meal breaks at the same time as colleagues?

- All of the time
- Most of the time
- Some of the time
- Rarely
- Never

16. Did you eat in the staff canteen?

- All of the time
- Most of the time

- Some of the time
- Rarely
- Never

17. Did you use shared rest facilities in your primary area of work (e.g. tea/break room)?

- All of the time
- Most of the time
- Some of the time
- Rarely
- Never

18. Did you use the doctors' mess during this time?

- All of the time
- Most of the time
- Some of the time
- Rarely
- Never

19. Did you wear hospital supplied scrubs at work?

- All of the time
- Most of the time
- Some of the time
- Rarely
- Never

20. Did you wear your own scrubs at work?

- All of the time
- Most of the time
- Some of the time
- Rarely
- Never

21. Did you wear your own clothes to work?

- All of the time
- Most of the time
- Some of the time
- Rarely
- Never

22. Did you use a changing room at work?

- All of the time
- Most of the time
- Some of the time
- Rarely
- Never

23. Did you have dedicated footwear for work during this time?

- All of the time
- Most of the time
- Some of the time
- Rarely
- Never

24. Did you wear your work clothes when leaving the hospital?

- All of the time
- Most of the time
- Some of the time
- Rarely
- Never

25. Did you use a reusable personal water/drinks bottle in your area of work?

- All of the time
- Most of the time
- Some of the time
- Rarely
- Never

26. How would you rate your adherence to trust policy hand-washing technique?

- All of the time
- Most of the time
- Some of the time
- Rarely
- Never

27. How would you rate your adherence to trust policy hand-washing frequency

- All of the time
- Most of the time
- Some of the time
- Rarely
- Never

28. Did you primarily work from home between March to May 2020?

- If yes, was this recommended for shielding reasons?

29. Did you primarily work from home from June to July 2020?

- If yes, was this recommended for shielding reasons?

30. Have you ever been recommended to shield by Occupational Health?

31. Have you ever been in a group that was recommended to shield by Public Health England? (yes/no)

**D. Behavioural**

1. Were you a smoker at any point between March to July 2020? (yes/no) If yes:

- Fewer than 5 per day
- 5-10 per day
- 10-20 per day
- >20 per day

2. Did you regularly drink alcohol between March to July 2020? (yes/no) If yes:

- Daily
- 2-3 times per week
- Once a week
- Less than once a week

3. How frequently did you visit a supermarket or shop between March to May 2020?

- Daily
- 2-3 times per week
- Once a week
- Less than once a week

4. How frequently did you visit a supermarket or shop between June to July 2020?

- Daily
- 2-3 times per week
- Once a week
- Less than once a week

5. How often did you have contact with people outside of your immediate household (not including work) between March to May 2020?

- Daily
- 2-3 times per week
- Once a week
- Less than once a week

6. How often did you have contact with people outside of your immediate household (not including work) between June to July 2020?

- Daily
- 2-3 times per week
- Once a week
- Less than once a week

7. How often did you order food deliveries (e.g. groceries, take-away) between March to May 2020?

- Daily
- 2-3 times per week
- Once a week

- Less than once a week

8. How often did you order food deliveries (e.g. groceries, take-away) between June to July 2020?

- Daily
- 2-3 times per week
- Once a week
- Less than once a week

9. How often did you exercise outdoors from March to May 2020?

- Daily
- 2-3 times per week
- Once a week
- Less than once a week

10. How often did you exercise outdoors June to July 2020?

- Daily
- 2-3 times per week
- Once a week
- Less than once a week

11. How often did you use public transport (not including travel to and from work) from March to May 2020?

- Daily
- 2-3 times per week
- Once a week
- Less than once a week

12. How often did you use public transport (not including travel to and from work) from June to July 2020?

- Daily
- 2-3 times per week
- Once a week
- Less than once a week

13. Did you use a facemask outside of work from March to May 2020?

- All of the time
- Most of the time
- Some of the time
- Rarely
- Never

14. Did you use a facemask outside of work from June to July 2020?

- All of the time
- Most of the time
- Some of the time
- Rarely
- Never

15. If you used a facemask outside of work, in which situations did you use one? (may be multiple)

- Social interaction
- Grocery shopping
- Commuting
- Exercising
- Other

16. Did you attend meetings or handovers where it was not possible to socially distance between March to May 2020? (yes/no)

17. Did you attend meetings or handovers where it was not possible to socially distance between June to July 2020? (yes/no)

**E. Co-morbidities**

1. What was your COVID risk-assessment group?

- Green
- Yellow
- Orange
- Red

2. Were your work duties altered because of your risk group? (yes/no)

3. Self-reported height [give measuring unit options ft/inches or m/cm]

4. Self-reported weight [give measuring usingt options st/lb or kg]

5. Have you even been told you are overweight in a medical setting? (yes/no)

6. Have you even been told you are obese in a medical setting? (yes/no

7. How often do you undertake physical exercise?

- Daily
- 2-3 times per week
- Once a week
- Less than once a week

8. Do you have any of the following co-morbidities:

Heart disease. (yes/no) If yes – select (may be multiple)

- Ischaemic heart disease
- Previous myocardial infarction (heart attack)
- Angina
- Valvular heart disease

- Other

Kidney disease. (yes/no) If yes – select

- Chronic kidney disease – not on dialysis
- Are you on haemodialysis?
- Are you on Peritoneal dialysis?
- Have you had a kidney transplant?
- Vasculitis
- Other

Lung disease. (yes/no) If yes – select (may be multiple)

- Chronic obstructive pulmonary disease (COPD)/ Chronic obstructive airway disease (COAD)
- Asthma
- Interstitial lung disease
- Bronchiectasis
- Emphysema
- Other

Have you ever been diagnosed with high blood pressure (yes/no). If yes:

- Are you on any medication. (yes/no)
- How many different medications (insert number)
- Is your blood pressure well controlled? (yes/no)

Type 1 diabetes (yes/no)

Type 2 diabetes (yes/no). If yes:

- Do you take insulin?
- How many medications do you take for diabetes? (must include zero)
- Is your blood sugar well controlled?

Do you have a compromised immune system due to any of the following?

- Immunosuppression drugs (yes/no)
- Blood disorder (including blood cancer) (yes/no)
- An inherited immune deficiency (yes/no)
- Other (free text) (yes/no)

Have you had a solid organ transplant (yes/no). If yes:

- Kidney
- Heart
- Lung
- Liver
- Small intestine
- Pancreas

Are you currently being treated for cancer? (yes/no). If yes:

- Solid organ cancer
- Blood cancer

- Skin cancer
- Other

9. Have you taken hydroxychloroquine at any time between March to July 2020? (yes/no). If yes:

- More than once daily
- Once daily
- 2 – 6 times a week
- Once a week
- Less than once a week

10. Did you take any of the following medication between March and July 2020 (may be multiple):

- Aspirin
- Angiotensin converting enzyme (ACE) inhibitors (including ramipril, lisinopril, captopril, enalopril and others)
- Angiotensin receptor blockers (ARBs) (including candesartan, irbesartan, losartan, valsartan and others)
- Tacrolimus
- Mycophenolate
- Hydroxychloroquine
- Prednisolone
- Tocilizumab
- Azathioprine
- Methotrexate
- Cyclosporine
- Leflunomide

11. Have you ever had any of the following medication (may be multiple):

- Rituximab
- Abatacept
- Adalimumab
- Etanercept
- Infliximab
- Basiliximab
- Cyclophosphamide

12. Have you ever had chemotherapy for cancer? (yes/no)

13. Have you ever had immunosuppressive medication not listed in the above questions? (yes/no). If yes:

- Free text
