# Supplementary material for: Demographic, behavioural and occupational risk factors associated with SARS-CoV-2 infection in UK healthcare workers: a retrospective observational study
Source: BMJ Open. 2022 Nov 7;12(11):e063159. doi: 10.1136/bmjopen-2022-063159 (PMC9644078; doi:10.1136/bmjopen-2022-063159)
Supplement: Supplementary data [file bmjopen-2022-063159supp002.pdf]

**Univariate logistic regression tables of variables assessed**

A. *Demographic factors and association with risk of SARS-CoV-2 infection in HCWs.*

| **Variable** | **OR** | **95 % CI** | ***p*-value** | n (positive) / N (responses) (%) |
|---|---|---|---|---|
| Number in househould (1) | 1 | - | - | 14/217 (6.5) |
| - 2 | 1.64 | 0.91 – 2.96 | 0.10 | 78/768 (10.1) |
| - 3 | 1.43 | 0.76 – 2.68 | 0.26 | 42/468 (9.0) |
| - 4 | 1.59 | 0.86 – 2.92 | 0.14 | 55/557 (9.9) |
| - 5 | 2.43 | 0.97 – 4.69 | 0.09 | 19/160 (11.9) |
| - 6 | 2.11 | 0.81 – 5.52 | 0.13 | 7/55 (12.7) |
| - 7 | 0.81 | 0.10 – 6.48 | 0.84 | 1/19 (5.2) |
| - 8 | 4.8 | 0.47 – 49.5 | 0.19 | 1/4 (25) |
| - 9 | 1 | - | - | 0/5 (0) |
| Rent room in shared house | 1.84 | 1.22 – 2.74 | 0.003 | 33/209 (16) |
| Live with other HCWs | 1.49 | 1.10 – 2.02 | 0.009 | 70/550 (13) |
| Live other key workers (not HCWs) | 0.95 | 0.70 – 1.29 | 0.73 | 63/663 (9.5) |

| | | | | |
|---|---|---|---|---|
| Multigenerational household | 0.96 | 0.73 – 1.27 | 0.79 | 104/1073 (9.7) |
| Children in household | 1.13 | 0.85 – 1.49 | 0.40 | 99/944 (10.5) |
| Number of children | | | | |
| - 0 | 1 | - | - | 126/1331 (9.5) |
| - 1 | | | | |
| - 2 | 1.04 | 0.72 – 1.50 | 0.84 | 43/439 (9.8) |
| - 3 | 1.28 | 0.70 – 2.35 | 0.42 | 13/110 (11.8) |
| - 4 | 1.91 | 0.41 – 8.83 | 0.41 | 2/12 (16.7) |
| - 5 | 2.39 | 0.27 – 21.6 | 0.44 | 1/5 (20) |
| School aged children | 0.97 | 0.72 – 1.32 | 0.86 | 66/684 (9.7) |
| Children attended school in March | 1.52 | 0.90 – 2.56 | 0.12 | 27/225 (12) |
| Children attended school in June | 0.58 | 0.35 – 0.97 | 0.038 | 30/395 (7.6) |
| Nursery age children | 0.69 | 0.38 – 1.23 | 0.20 | 13/183 (7.1) |
| Children attend nursery March | 1.91 | 0.56 – 6.51 | 0.30 | 6/72 (8.3) |
| Children attend nursery June | 1.05 | 0.31 – 3.55 | 0.94 | 9/125 (7.2) |
| People >65 in household | 1.05 | 0.65 – 1.68 | 0.86 | 21/207 (10.1) |
| Household member positive PCR test | 3.48 | 2.09 – 5.78 | <0.0001 | 22/84 (26) |

| | | | | |
|---|---|---|---|---|
| Household member positive Ab test | 11.29 | 7.08 – 18.01 | <0.0001 | 40/79 (51) |
| Household member symptomatic | 3.71 | 2.8 – 4.96 | <0.0001 | 95/437 (22) |
| Travel to work | | | | |
| - Drive | 1 | - | - | 133/1449 (9.2) |
| - Walk | 1.28 | 0.78 – 2.10 | 0.34 | 20/175 (11.4) |
| - Cycle | 1.19 | 0.84 – 1.69 | 0.33 | 47/438 (10.7) |
| - Bus | 1.15 | 0.63 – 2.10 | 0.65 | 13/125 (10.4) |
| - Train | 1.62 | 0.75 – 3.48 | 0.22 | 8/57 (14.0) |
| Share a car | 1.49 | 0.84 – 2.61 | 0.17 | 15/109 (13.8) |

*B.* Socioeconomic *factors and association with risk of SARS-CoV-2 infection in HCWs.*

| **Variable** | **OR** | **95 % CI** | ***p*-value** | n (positive) / N (responses) (%) |
|---|---|---|---|---|
| Born in UK | 0.59 | 0.44 – 0.79 | <0.001 | 136/1616 (8.4) |
| Ethnicity | 1.06 | 1.03 – 1.10 | <0.001[a] | 1584/2258[b] |
| Highest level of education | | | | |
| - Higher degree | 1 | - | - | 84/869 (9.7) |
| - GCSE | 1.02 | 0.61 – 1.71 | 0.94 | 20/203 (9.9) |
| - A level | 0.90 | 0.54 – 1.51 | 0.70 | 20/227 (8.8) |
| - Undergraduate degree | 1.15 | 0.83 – 1.59 | 0.40 | 79/722 (10.9) |
| - Other vocational training | 0.75 | 0.44 – 1.30 | 0.31 | 17/228 (7.5) |
| More than one job | 0.95 | 0.61 – 1.49 | 0.84 | 24/254 (9.5) |
| Other dependents | 1.21 | 0.63 – 2.30 | 0.57 | 11/95 (11.6) |
| Care outside of household | 0.57 | 0.34 – 0.11 | 0.056 | 19/232 (8.2) |

[a] p-value for likelihood ratio test

[b] number of participants identifying as white British

C. *Occupational factors and association with risk of SARS-CoV-2 infection in HCWs.*

| **Variable** | **OR** | **95 % CI** | **p-value** | **n (positive) / N (responses) (%)** |
|---|---|---|---|---|
| Job role | | | | |
| - Administrative staff | 1 | - | - | 24/336 (7.1) |
| - Staff nurse | 2.02 | 1.18 – 3.43 | 0.01 | 40/298 (13.4) |
| - Senior nursing staff | 1.54 | 0.89 – 2.67 | 1.55 | 33/311 (10.6) |
| - Consultant | 1.66 | 0.86 – 3.19 | 0.13 | 17/150 (11.3) |
| - Junior doctor | 1.67 | 0.77 – 3.63 | 0.20 | 10/88 (11.4) |
| - Laboratory staff | 0.58 | 0.23 – 1.44 | 0.24 | 6/141 (4.3) |
| - Healthcare assistant | 1.71 | 0.93 – 3.15 | 0.08 | 22/189 (11.6) |
| - Theatre staff | 0.54 | 0.07 – 4.18 | 0.56 | 1/25 (4) |
| - Manager | 1.75 | 0.87 – 3.51 | 0.12 | 14/118 (11.9) |
| - Radiographer | 1.39 | 0.54 – 3.56 | 0.69 | 6/62 (9.7) |
| - Midwife | 0.20 | 0.27 – 1.53 | 0.12 | 1/65 (1.5) |

| | | | | |
|---|---|---|---|---|
| - Physio | 4.33 | 1.83 – 10.25 | 0.001 | 9/36 (25) |
| - Pharmacy staff | 2.07 | 0.84 – 5.08 | 0.11 | 7/51 (13.7) |
| - Cleaning/domestic staff | 1 | - | - | 0/6 (0) |
| - Dietician | 0.59 | 0.076 – 4.57 | 0.61 | 1/23 (4.4) |
| - Occupational therapist | 0.87 | 0.11 – 6.84 | 0.89 | 1/16 (6.25) |
| - Speech and Language therapist | 2.29 | 0.63 – 8.38 | 0.48 | 3/20 (15) |
| - Porter | 2.17 | 0.25 – 18.7 | 0.48 | 1/7 (14.3) |
| - Other | 1.17 | 0.05 – 0.12 | 0.59 | 26/314 (8.3) |
| Direct patient care COVID | 1.86 | 1.41 – 2.47 | <0.001 | 103/757 (13.6) |
| Worked in red area | 1.78 | 1.33 – 2.38 | <0.001 | 85/618 (13.8) |
| Time in red area | 0.99 | 0.83 – 1.20 | 0.99 | - |
| Specialty | | | | |
| - Non-patient facing | 1 | - | - | 10/169 (5.9) |
| - Emergency department | 1.32 | 1.10 – 5.77 | 0.44 | 44/574 (7.7) |
| - Critical care | 2.51 | 1.10 – 5.77 | 0.029 | 16/117 (13.7) |
| - Acute medicine | 4.57 | 2.08 – 10.07 | <0.001 | 23/103 (22.3) |
| - Respiratory medicine | 2.0 | 0.51 – 7.74 | 0.32 | 3/27 (11.1) |

| | | | | |
|---|---|---|---|---|
| - Infectious diseases | 1.59 | 0.32 – 7.78 | 0.57 | 2/22 (9.1) |
| - Medical specialties | 4.35 | 2.01 – 9.42 | <0.001 | 26/121 (21.5) |
| - Theatres | 2.01 | 0.80 – 5.04 | 0.14 | 10/89 (11.2) |
| - ENT | 0.66 | 0.08 – 5.41 | 0.70 | 1/25 (4) |
| - Surgical | 2.71 | 1.24 – 5.93 | 0.012 | 22/151 (14.6) |
| - Paediatrics | 0.71 | 0.24 – 2.13 | 0.54 | 5/117 (4.3) |
| - Research | 1.44 | 0.64 – 3.25 | 0.37 | 17/204 (8.3) |
| - Other | 1.27 | 0.61 – 2.66 | 0.53 | 11/101 (10.9) |
| Average hours per week March | 1.02 | 0.96 – 1.09 | 0.49 | - |
| Average hours per week June | 0.99 | 0.93 – 1.06 | 0.84 | - |
| Work nights | 1.68 | 1.26 – 2.25 | <0.001 | 82/604 (13.6) |
| Present for AGPs | 1.30 | 0.93 – 1.84 | 0.13 | 47/396 (11.9) |
| Receive formal PPE training | 1.40 | 1.05 – 1.85 | 0.02 | 129/1141 (11.3) |
| Adequate PPE available | | | | |
| - All of the time | 1 | - | - | 83/1038 (8.0) |
| - Most of the time | 1.34 | 0.98 – 1.83 | 0.065 | 92/882 (10.4) |
| - Some of the time | 1.93 | 1.22 – 3.05 | 0.005 | 28/195 (14.4) |

| | | | | |
|---|---|---|---|---|
| - Rarely | 3.60 | 1.71 – 7.57 | 0.001 | 10/42 (23.8) |
| Use mask at work before widespread | 0.98 | 0.86 – 1.12 | 0.79 | 127/1198 (10.6) |
| Which mask when mandatory | 0.94 | 0.81 – 1.10 | 0.45 | - |
| What type of eye protection | 1.05 | 0.97 – 1.14 | 0.25 | - |
| Rest/meal with colleagues | | | | |
| - Never | 1 | - | - | 21/297 (7.1) |
| - All of the time | 1.49 | 0.81 – 2.76 | 0.20 | 24/235 (10.2) |
| - Most of the time | 1.99 | 1.19 – 3.33 | 0.009 | 64/487 (13.1) |
| - Some of the time | 1.52 | 0.92 – 2.51 | 0.10 | 76/733 (10.4) |
| - Rarely | 1.05 | 0.60 – 1.85 | 0.86 | 35/472 (7.4) |
| Eat in staff canteen | 1.08 | 0.99 – 1.17 | 0.06 | - |
| Shared rest areas | 0.99 | 0.90 – 1.11 | 0.96 | - |
| Use doctors mess | 1.77 | 1.17 – 2.69 | 0.007 | 30/195 (15.4) |
| Hospital supplied scrubs | 1.15 | 1.04 – 1.27 | 0.007 | 124/1056 (11.7) |
| Own scrubs | 1.04 | 0.88 – 1.23 | 0.62 | 26/256 (10.2) |
| Own clothes to work | 0.95 | 0.80 – 1.13 | 0.54 | 165/1684 (9.8) |
| Use changing room at work | 1.04 | 0.92 – 1.18 | 0.52 | 139/1323 (10.5) |

| | | | | |
|---|---|---|---|---|
| Dedicated footwear for work | 1.14 | 0.97 – 1.33 | 0.11 | 151/1390 (10.9) |
| Wear own clothes when going home | 1.00 | 0.87 – 1.16 | 0.95 | 90/937 (9.6) |
| Re-usable water bottle | 1.15 | 0.98 – 1.29 | 0.56 | 178/1760 (10.1) |
| Adherence to handwashing technique | 1.15 | 0.88 – 1.51 | 0.31 | - |
| Handwashing frequency | 1.00 | 0.76 – 1.32 | 0.99 | - |
| Work from home March | 0.60 | 0.39 – 0.91 | 0.016 | 27/410 (6.6) |
| - For shielding? | 0.58 | 0.36 | 0.94 | 4/62 (6.5) |
| Work from home June | 0.58 | 0.36 – 0.94 | 0.026 | 2-/314 (6.4) |
| - For shielding? | 1.81 | 0.63 – 5.22 | 0.27 | 5/50 (10) |

*D. Behavioural factors and association with risk of SARS-CoV-2 infection in HCWs.*

| Variable | OR | 95 % CI | p-value | n (positive) / N (responses) (%) |
|---|---|---|---|---|
| Smoker | 0.37 | 0.18 – 0.76 | 0.007 | 8/196 (4.1) |
| Quantity smoked | 0.54 | 0.20 – 1.48 | 0.23 | - |
| Alcohol | 0.74 | 0.55 – 0.98 | 0.038 | 78/864 (4.1) |
| Frequency of alcohol | 0.91 | 0.66 – 1.24 | 0.54 | - |
| Shopping frequency March | 0.91 | 0.75 – 1.10 | 0.32 | - |
| Shopping frequency June | 0.90 | 0.74 – 1.08 | 0.26 | - |
| Contact with people March | 1.04 | 0.86 – 1.25 | 0.72 | - |
| Contact with people June | 1.06 | 0.90 – 0.25 | 0.50 | - |
| Food deliveries march | | | | |
| - Less than once/week | 1 | - | - | 141/1406 (10.0) |
| - Once a week | 0.96 | 0.70 – 1.31 | 0.81 | 64/661 (9.7) |
| - 2-3 times / week | 0.68 | 0.35 – 1.31 | 0.25 | 10/143 (7.0) |
| - Daily | 5.38 | 1.27 – 22.8 | 0.022 | 3/8 (37.5) |
| Food deliveries June | | | | |

| | | | | |
|---|---|---|---|---|
| - Less than once/week | 1 | - | - | 136/1376 (9.9) |
| - Once a week | 0.96 | 0.70 – 1.30 | 0.78 | 66/695 (9.5) |
| - 2-3 times / week | 1.01 | 0.58 – 1.78 | 0.96 | 15/150 (10) |
| - Daily | 6.10 | 1.01 – 36.7 | 0.049 | 2/5 (40) |
| Exercise outdoors March | | | | |
| - Less than once/week | 1 | - | - | 53/428 (12.4) |
| - Once a week | 1.07 | 0.69 – 1.66 | 0.77 | 40/305 (13) |
| - 2-3 times / week | 0.73 | 0.50 – 1.06 | 0.10 | 72/770 (9.4) |
| - Daily | 0.58 | 0.39 – 0.86 | 0.007 | 54/718 (7.5) |
| Exercise outdoors June | | | | |
| - Less than once/week | 1 | - | - | 51/405 (12.6) |
| - Once a week | 0.89 | 0.57 – 1.41 | 0.63 | 35/307 (11.4) |
| - 2-3 times / week | 0.76 | 0.52 – 1.10 | 0.14 | 79/804 (9.8) |
| - Daily | 0.56 | 0.37 – 0.84 | 0.005 | 53/709 |
| Public transport March | 0.81 | 0.64 – 1.04 | 0.10 | - |
| Public transport June | 0.80 | 0.63 – 1.01 | 0.06 | - |
| Facemask March | 0.99 | 0.90 – 1.10 | 0.86 | - |

| | | | | |
|---|---|---|---|---|
| Facemask June | 1.0 | 0.88 – 1.13 | 1.0 | - |
| No social distancing March | 1.74 | 1.31 – 2.30 | <0.0001 | 127/1021 (12.4) |
| No social distancing June | 1.31 | 1.0 – 1.73 | 0.06 | 105/933 (11.3) |
| Work duties altered for risk | 1.12 | 0.75 – 1.69 | 0.58 | 40/278 (10.8) |

*E. Health factors and association with risk of SARS-CoV-2 infection in HCWs.*

| **Variable** | **OR** | **95 % CI** | **p-value** | **n (positive) / N (responses) (%)** |
|---|---|---|---|---|
| Told overweight | 0.88 | 0.63 – 1.23 | 0.46 | 49/543 (9.0) |
| Told obese | 0.93 | 0.58 – 1.49 | 0.77 | 21/225 (9.3) |
| Exercise frequency | | | | |
| - 2-3 times/week | 1 | - | - | 101/919 (11.0) |
| - Daily | 0.67 | 0.46 – 0.97 | 0.033 | 43/566 (7.6) |
| - Once a week | 0.91 | 0.61 – 1.35 | 0.64 | 37/367 (10.1) |
| - < once a week | 0.91 | 0.61 – 1.34 | 0.63 | 39/387 (10.1) |
| Heart disease | 1.17 | 0.41 – 3.34 | 0.77 | 4/35 (11.4) |
| Lung disease | 0.74 | 0.36 – 1.55 | 0.43 | 8/106 (7.6) |
| Kidney disease | - | - | - | 0/10 (0) |
| High BP | 0.75 | 0.46 – 1.23 | 0.26 | 19/244 (7.8) |
| - BP medicated | 1.03 | 0.35 – 1.97 | 0.96 | 14/175 (8.0) |
| - Medication # | 0.89 | 0.55 – 1.46 | 0.65 | - |
| T1 DM | 2.01 | 0.43 – 9.38 | 0.37 | 2/11 (18.2) |
| T2 DM | 1.17 | 0.46 – 3.02 | 0.74 | 5/44 (11.4) |

| | | | | |
|---|---|---|---|---|
| - Insulin | - | - | - | 0/3 (0) |
| - Medication # | 0.75 | 0.29 – 1.91 | 0.54 | - |
| Immunosuppression | 1.3 | 0.51 – 3.36 | 0.59 | 5/40 |
| Blood disorder | 1.84 | 0.40 – 8.4 | 0.43 | 2/12 (16.7) |
| Inherited | 1.65 | 0.36 – 7.51 | 0.52 | 2/13 (15.4) |
| Organ transplant | - | - | - | 0/0 (0) |
| Cancer treatment | - | - | - | 0/9 (0) |
| Currently taking: | | | | |
| - Hydroxychloroquine | 0.48 | 0.06 – 3.6 | 0.48 | 1/20 (5.0) |
| - Aspirin | 1.0 | 0.58 – 1.74 | 0.99 | 15/152 (9.9) |
| - ACE inhibitors | 1.52 | 0.74 – 3.12 | 0.25 | 9/64 (14.1) |
| - ARBs | 0.74 | 0.23 – 2.42 | 0.62 | 3/40 (7.5) |
| - Tacrolimus | 2.30 | 0.26 – 20.66 | 0.46 | 1/5 (20) |
| - Mycophenolate | 2.30 | 0.26 – 20.66 | 0.46 | 1/5 (20) |
| - Prednisolone | 1.59 | 0.61 – 4.16 | 0.34 | 5/34 (14.7) |
| - Tocilizumab | 1.84 | 0.21 – 15.8 | 0.58 | 1/6 (16.7) |
| - Azathioprine | 2.31 | 0.49 – 10.92 | 0.29 | 2/10 (20) |

| | | | | |
|---|---|---|---|---|
| - Methotrexate | 1.15 | 0.26 – 5.03 | 0.86 | 2/18 (11.1) |
| - Cyclosporine | 2.30 | 0.26 – 20.66 | 0.46 | 1/5 (20) |
| - Leflunomide | 1.53 | 0.18 – 12.77 | 0.69 | 1/7 (14.3) |
| Ever had: | | | | |
| - Rituximab | 0.83 | 0.11 – 6.48 | 0.86 | 1/12 (8.3) |
| - Abatacept | 2.31 | 0.26 – 20.66 | 0.46 | 1/5 (20) |
| - Adalimumab | 1.15 | 0.14 – 9.21 | 0.90 | 1/9 (11.1) |
| - Etanercept | 0.83 | 0.11 – 6.48 | 0.86 | 1/12 (8.3) |
| - Infliximab | 2.31 | 0.49 – 10.92 | 0.29 | 2/10 (20) |
| - Basiliximab | 2.30 | 0.26 – 20.66 | 0.46 | 1/5 (20) |
| - Cyclophosphamide | 0.76 | 0.10 – 5.90 | 0.80 | 1/13 (7.7) |
| Chemotherapy for cancer | 0.65 | 0.20 – 2.11 | 0.47 | 3/45 (6.7) |
