# Supplementary material for: Demographic, behavioural and occupational risk factors associated with SARS-CoV-2 infection in UK healthcare workers: a retrospective observational study
Source: BMJ Open. 2022 Nov 7;12(11):e063159. doi: 10.1136/bmjopen-2022-063159 (PMC9644078; doi:10.1136/bmjopen-2022-063159)
Supplement: Author's manuscript [file bmjopen-2022-063159.draft_revisions.pdf]

BMJ Open is committed to open peer review. As part of this commitment we make the peer review history of every article we publish publicly available.

When an article is published we post the peer reviewers' comments and the authors' responses online. We also post the versions of the paper that were used during peer review. These are the versions that the peer review comments apply to.

The versions of the paper that follow are the versions that were submitted during the peer review process. They are not the versions of record or the final published versions. They should not be cited or distributed as the published version of this manuscript.

BMJ Open is an open access journal and the full, final, typeset and author-corrected version of record of the manuscript is available on our site with no access controls, subscription charges or pay-per-view fees (http://bmjopen.bmj.com).

If you have any questions on BMJ Open's open peer review process please email info.bmjopen@bmj.com

BMJ Open

A retrospective observational study of demographic, behavioural and occupational risk factors associated with SARS-COV-2 infection in UK healthcare workers

Journal:	*BMJ Open*
Manuscript ID	bmjopen-2022-063159
Article Type:	Original research
Date Submitted by the Author:	23-Mar-2022
Complete List of Authors:	Cooper, Daniel; University of Cambridge School of Clinical Medicine, Department of Medicine ; Cambridge University Hospitals NHS Foundation Trust<br>Lear , Sara; Cambridge University Hospitals NHS Foundation Trust<br>Sithole, Nyarie; Cambridge University Hospitals NHS Foundation Trust<br>Shaw, Ashley; Cambridge University Hospitals NHS Foundation Trust, Medical Director's Office<br>Stark , Hannah; NIHR Cambridge Biomedical Research Centre, NIHR Bioresource<br>Ferris, Mark; Cambridge University Hospitals NHS Foundation Trust, Occupational Health<br>COVID-19 collaboration, CITIID-NIHR BioResource ; NIHR Cambridge Biomedical Research Centre, NIHR Bioresource<br>Bradley, John; University of Cambridge, Department of Medicine; Cambridge University Hospitals NHS Foundation Trust<br>Maxwell, Patrick ; University of Cambridge School of Clinical Medicine, Department of Medicine ; Cambridge University Hospitals NHS Foundation Trust<br>Goodfellow, Ian; University of Cambridge, Department of Pathology, Division of Virology<br>Weekes, Michael; University of Cambridge; Cambridge University Hospitals NHS Foundation Trust<br>Seaman, Shaun; MRC Biostatistics Unit,<br>Baker, Stephen; University of Cambridge School of Clinical Medicine, Cambridge Institute of Therapeutic Immunology and Infectious Disease
Keywords:	COVID-19, Public health < INFECTIOUS DISEASES, Infection control < INFECTIOUS DISEASES, Epidemiology < INFECTIOUS DISEASES

SCHOLARONE™ Manuscripts

I, the Submitting Author has the right to grant and does grant on behalf of all authors of the Work (as defined in the below author licence), an exclusive licence and/or a non-exclusive licence for contributions from authors who are: i) UK Crown employees; ii) where BMJ has agreed a CC-BY licence shall apply, and/or iii) in accordance with the terms applicable for US Federal Government officers or employees acting as part of their official duties; on a worldwide, perpetual, irrevocable, royalty-free basis to BMJ Publishing Group Ltd ("BMJ") its licensees and where the relevant Journal is co-owned by BMJ to the co-owners of the Journal, to publish the Work in this journal and any other BMJ products and to exploit all rights, as set out in our licence.

The Submitting Author accepts and understands that any supply made under these terms is made by BMJ to the Submitting Author unless you are acting as an employee on behalf of your employer or a postgraduate student of an affiliated institution which is paying any applicable article publishing charge ("APC") for Open Access articles. Where the Submitting Author wishes to make the Work available on an Open Access basis (and intends to pay the relevant APC), the terms of reuse of such Open Access shall be governed by a Creative Commons licence – details of these licences and which Creative Commons licence will apply to this Work are set out in our licence referred to above.

Other than as permitted in any relevant BMJ Author's Self Archiving Policies, I confirm this Work has not been accepted for publication elsewhere, is not being considered for publication elsewhere and does not duplicate material already published. I confirm all authors consent to publication of this Work and authorise the granting of this licence.

For peer review only

A retrospective observational study of demographic, behavioural and occupational risk factors associated with SARS-COV-2 infection in UK healthcare workers

Daniel J. Cooper[1, 2, 3*], Sara Lear[2], Nyarie Sithole[2], Ashley Shaw[2], Hannah Stark[4], Mark Ferris[2], CITIID-NIHR BioResource COVID-19 collaboration consortium, John R. Bradley[1, 2], Patrick H. Maxwell[1, 2], Ian Goodfellow[5], Michael P. Weekes[1, 2, 6], Shaun Seaman[7], Stephen Baker[8]

1. Department of Medicine, Cambridge University School of Clinical Medicine, Cambridge, UK.
2. Cambridge University Hospitals NHS Foundation Trust, Cambridge, UK.
3. Global and Tropical Health Division, Menzies School of Heath Research and Charles Darwin University, Darwin, Northern Territory, Australia
4. NIHR Bioresource, NIHR Cambridge Biomedical Research Centre, Cambridge Biomedical Campus, Cambridge, UK
5. Department of Pathology, Division of Virology, University of Cambridge, Cambridge Biomedical Campus, Cambridge, UK
6. Cambridge Institute for Medical Research, Cambridge, UK
7. MRC Biostatistics Unit, University of Cambridge, Cambridge, UK
8. Cambridge Institute of Therapeutic Immunology and Infectious Disease, University of Cambridge School of Clinical Medicine, Cambridge Biomedical Campus, Cambridge, UK.

* Corresponding author

Keywords: COVID-19; Healthcare Workers; Epidemiology; Public Health

Word count: 3108

For peer review only - http://bmjopen.bmj.com/site/about/guidelines.xhtml

Corresponding author:

Dr Daniel Cooper

Department of Medicine

University of Cambridge

E: Dc801@cam.ac.uk

T: 07843662562

For peer review only

ABSTRACT

Objective: Healthcare workers (HCWs) are at higher risk of SARS-COV-2 infection than the general population. This group are pivotal to healthcare system resilience during the COVID-19, and future, pandemics. We investigated demographic, social, behavioural and occupational risk factors for SARS-CoV-2 infection amongst HCWs.

Design/Setting/Participants: HCWs enrolled in a large-scale sero-epidemiological study at a UK university teaching hospital were sent questionnaires spanning a 5-month period from March–August 2021. Univariate logistic regression was used to assess factors associated with risk of SARS-COV- 2 infection. A Least Absolute Shrinkage Selection Operator (LASSO) regression model was used to identify variables to include in a multivariate logistic regression model.

Results: Amongst 2,258 HCWs, highest Odds Ratios associated with SARS-CoV-2 antibody seropositivity on multivariate analysis were having a household member previously testing positive for SARS-CoV-2 antibodies (OR 6.94 [95% CI 4.15 – 11.6]; $p<0.0001$) and being of Black ethnicity (6.21 [95% CI 2.69 – 14.3]; p<0.0001). Occupational factors associated with a higher risk of seropositivity included working as a physiotherapist (OR 2.78 [95% CI 1.21 – 6.36]; $p=0.015$) and working predominantly in acute medicine (OR 2.72 [95% CI 1.57 – 4.69]; p=<0.0001) or medical subspecialities (not including infectious diseases) (OR 2.33 [95% CI 1.4 – 3.88]; $p=0.001$). Reporting that adequate PPE was "rarely" available had an OR of 2.83 (95% CI 1.29 – 6.25; p=0.01). Reporting attending a handover where social distancing was not possible had an OR of 1.39 (95% CI 1.02 – 1.9; $p=0.038$).

Conclusions: Novel SARS-CoV-2 variants and potential vaccine-escape continue to threaten stability of healthcare systems worldwide and sustained vigilance against HCW infection remains a priority.

For peer review only - http://bmjopen.bmj.com/site/about/guidelines.xhtml

Enhanced risk assessments should be considered for HCWs of Black ethnicity, physiotherapists and those working in acute medicine or medical subspecialties. Workplace risk reduction measures include ongoing access to high-quality PPE and effective social distancing measures.

Strengths and limitations of this study:

- A strength of this study was the use of a large, well-defined cohort of UK healthcare workers
- The identification of actionable risk factors for mitigation of HCW infection
- Representative and transferable conclusions for acute hospital trusts
- Limitations include some potential retrospective recall bias of subjective questionnaire responses

For peer review only

BACKGROUND

The COVID-19 pandemic continues to overwhelm healthcare services globally with substantial morbidity and mortality [1]. The COVID-19 vaccination programme has been a major success in the UK, having a major impact on reducing hospitalisations and death[2 3]. However, the recent upsurge of cases associated with the delta variant[4] followed by emergence and dominance of the Omicron variant[4] illustrates how management of the pandemic requires sustained vigilance from the general public, policy makers, and healthcare workers (HCWs). Notably, the delta[5] and omicron[6] variants have increased transmissibility, and a reduced efficacy of vaccination for prevention of infection [7-10]. Therefore, the emergence of additional variants with the potential for vaccine escape are a genuine concern for how we control SARS-CoV-2 in the long term.

HCWs are at a disproportionately high risk of infection from SARS-CoV-2[11] but remain key to the resilience of the health service during this, and all future pandemics. Infections of HCWs with SARS-CoV-2 and the isolation of contacts has resulted in significant staff shortages and increased strain on UK hospitals. Staff absence during September 2021 (most recent available figures) was 5.4% across the NHS; higher than August 2021 (5.1%) and higher than September 2020 (4.2%) [12]. This high level of absence is despite the high rates of vaccination in HCWs, where up to 92.3% of staff in NHS trusts have received at least 2 doses of vaccine as of 28th February 2022 [13]. Measures to reduce the risk of SARS-CoV-2 exposure to HCWs alongside widespread vaccination are vital to create resilience within the healthcare system. We have previously identified several occupational factors associated with increased risk of SARS-CoV-2 seropositivity in HCWs, which included job role, work location and ethnicity[14]. We conducted a retrospective observational cohort study in HCWs working in a major tertiary referral centre in the East of England with the objective of further elucidating the social, demographic, occupational and physical factors that may contribute to a higher risk of SARS-CoV-2 infection in HCWs.

Methods

For peer review only - http://bmjopen.bmj.com/site/about/guidelines.xhtml

Population and setting

Cambridge University Hospitals NHS Foundation Trust (CUH) is a tertiary referral centre and teaching hospital with 1,000 beds and 11,545 staff serving a population of 580,000 people in the East of England. The facility was equipped with 43 ICU beds prior to the pandemic, rising to 103 ICU beds at the peak of the pandemic, and an Emergency Department that receives ~14,000 attendees a month. During the study period (between March and June 2020), CUH treated 525 patients with PCR-confirmed COVID-19. The peak of COVID-19 admissions occurred in late March and early April 2020, with comparatively few COVID-19 admissions from June 2020 to November 2020. The definition of COVID-19 working for the purpose of risk stratification included clinical areas designated as either “Red” (patients with PCR-confirmed SARS-CoV-2 infection) or “Amber” (patients for whom there is a high clinical suspicion of COVID-19).

According to the 2011 England and Wales census[15], 85·3% of the population of the East of England are White British, 5·5% are White Other, 4·8% are Asian, 2% are Black, and 1·9% are of Mixed ethnicity. The proportion of Black, Asian, and Minority Ethnic (BAME) staff employed at CUH at the time of the study was largely representative of the overall NHS workforce[17] (21·2% vs 20·7%, respectively).

A staff screening programme for SARS-CoV-2 serological testing was available from 10th June 2020 to 7th August 2020, and has been described previously[14]. In brief, all staff members were invited by email to participate in the serological screening programme and asked to self-refer for a clinic appointment. Written informed consent was obtained from all participants enrolled into this study. As part of this process all participants were invited to join the NIHR BioResource – COVID-19 Research Cohort (IRAS 220277). Basic demographic and occupational information were recorded and a serum sample was taken and assayed for total SARS-CoV-2 antibodies (detailed below).

Questionnaire

For peer review only - http://bmjopen.bmj.com/site/about/guidelines.xhtml

A questionnaire covering demographic, occupational and behavioural factors potentially associated with risk of infection was designed with input from infectious disease physicians, occupational physicians, virologists, microbiologists, and epidemiologists. Participants previously enrolled in a longitudinal HCW serological study (as described above) were invited by email to complete an online form containing the questionnaire. A copy of the questions included in this questionnaire is included as **appendix 1/supplementary file 1**. Questionnaire invites were sent between October and November 2020 and covered the period between March 2020 and June 2020 (the time of serological sampling). Questions relating to behavioural and demographic factors were separated by time periods covering March – May and June – July to account for differences in behaviour and exposures outside of occupational environments due to the instigation (March 2020) and easing (June 2021) of the first UK national "lockdown" measures.

Laboratory assays

Serological testing for antibodies directed against SARS-CoV-2 was performed using the Centaur XP SARS-Cov-2 Total Antibody assay (Siemens Healthcare Limited, Surrey, UK). This method is a fully automated high throughput enzyme linked chemiluminescent bridging immunoassay which targets the S1RBD antigen of SARS-CoV-2 and can detect all Ig subclasses (IgG, IgM, and IgA). The method was independently validated by Public Health England and has a reported sensitivity and specificity of 98.1% (95% CI 96.6 – 99.1) and 99.9% (95% CI 99.4 – 100)[16] respectively. Samples were processed in the Biochemistry laboratory at CUH following the SOP as stated by the manufacturer in their Instruction for Use (IFU) after a local verification using guidance from The Royal College of Pathologists[17].

Statistical analysis

Univariate logistic regression was used to assess each variable in the questionnaire for association with positive SARS-CoV-2 antibodies. Variables with a p-value of <0.05 on univariate analysis were included in a Least Absolute Shrinkage and Selection Operator (LASSO) regression analysis with post-estimation extended Bayesian information criterion commands for variable selection to include

For peer review only - http://bmjopen.bmj.com/site/about/guidelines.xhtml

in a multivariate logistic regression model. The LASSO method of variable selection was used, in preference to the older stepwise selection method, because it has been shown to lead to higher prediction accuracy and variable selection that is less sensitive to small changes in the data[18 19]. Variables selected by LASSO analysis were included in a final multivariate logistic regression model.

Ethical Approval

Ethical approval for this study was granted by the East of England – Cambridge Central Research Ethics Committee (IRAS ID: 220277).

Patient and public involvement

Staff at CUH contributed to study and questionnaire design.

RESULTS

Baseline characteristics

A total of 2,258 HCW responded to the invitation to complete an online questionnaire. Of the participants that responded to join the study, 19.65% (400/2,044 responses) were male, the median age was 42 years (IQR 32 – 53 years), and 27.7% (618/2,044) reported working in a designated COVID-19 "red" area during this first wave of the pandemic. Notably, 9.8% (n=222/2,044) of the cohort tested seropositive for SARS-CoV-2 antibodies. The demographics of the study group are shown in **Table 1**.

Univariate analysis

Reponses demonstrated to have a significant association (p<0.05) with seropositivity in a univariate analysis are described in **Table 2 (**The odds ratios and p-values for responses to all questions are listed in **supplementary Table 1)**. Noteworthy variables significantly associated with seropositivity for SARS-CoV-2 antibodies included having a household member that had tested positive for SARS-CoV-2 by PCR prior to staff serology testing (OR 3.48 [95% CI 2.09 – 5.78]; p<0.001), or had tested positive by a SARS-CoV-2 antibody test (OR 11.3 [95% CI 7.08 – 18.01]; p<0.001), or had had a

For peer review only - http://bmjopen.bmj.com/site/about/guidelines.xhtml

For peer review only

household member who had been symptomatic (OR 3.71 [95% CI 2.8 – 4.96]; *p*<0.001). Other demographic factors that were positively associated with seropositivity include identifying as being Asian or Asian British (other), mixed ethnicity, or Black or Black British (African) ethnicity. Notably, reporting being born in the UK was associated with a protective effect (OR 0.59 [95% CI 2.8 – 4.96]; *p*<0.001).

Renting a room in a shared house (OR 1.84 [1.22 – 2.74]; *p*=0.003) and living with another healthcare worker (OR 1.49 [95% CI 1.10 – 2.02]; *p*=0.009) were further demographic factors associated with a significantly higher risk of infection on univariate analysis.

Other than job role, specialty and direct COVID patient care, a number of other occupational factors were associated with higher odds of infection, including working night shifts (OR 1.68 [1.26 – 2.25]; *p*<0.001), using the doctors' mess (OR 1.77 [1.17 – 2.69]; *p*=0.007), spending rest or meal time with colleagues "most of the time" (OR 1.99 [95% CI 1.19 – 3.33]; *p*=0.009), and using hospital supplied scrubs (OR 1.15 [95% CI 1.04 – 1.27]; *p*=0.007). Those reporting having received formal PPE training had a 40% higher risk of infection (OR 1.4 [95% CI 1.05 – 1.85]; *p*=0.02) than those who did not. A higher proportion of those that worked in COVID red areas reported receiving formal PPE training (486/613, 79%) than those not working in COVID red areas (646/1594, 41%; *p*<0.0001), and formal PPE training no longer remained significant when controlling for "red area" working (OR 1.2 [0.88 – 1.63]; *p*=0.20).

Those reporting having adequate PPE available "some of the time" (OR 1.93 [95% CI 1.22 – 3.05]; *p*=0.005) or "rarely" (OR 3.60 [95% CI 1.71 – 7.57]; *p*=0.001) were associated with a higher odds of infection compared to those who reported adequate PPE being available "all of the time". A higher proportion of those reporting PPE being available "some of the time" (78/194, 40%) worked in COVID red areas compared to those reporting PPE being available "all of the time" (540/2041, 27%; p<0.0001). Attending shift handover (a staff meeting prior to shift change) where social distancing was not possible was associated with a higher risk of infection (OR 1.74 [95% CI 1.31 – 2.30];

For peer review only - http://bmjopen.bmj.com/site/about/guidelines.xhtml

$p<0.001$). Working predominantly from home between March – June 2020 was associated with a protective effect (OR 0.60 [95% CI 0.39 – 0.91]; $p=0.016$), as was working from home between June – July 2019 (OR 0.58 [95% CI 0.36 – 0.94]; $p=0.026$)

Reporting being a smoker was associated with a lower risk of infection (OR 0.37 [95% CI 0.18 – 0.76]; $p=0.007$) amongst behavioural risk factors. Reporting drinking alcohol was associated with a lower risk of infection (OR 0.74 [95% CI 0.55 – 0.98]; $p=0.38$), however frequency of drinking alcohol had no effect on risk of infection. Having food or grocery deliveries to home "daily" was associated with a higher risk of infection between March – May 2020 (OR 5.38 [95% CI 1.27 – 22.8]; $p=0.022$) and June to July 2020 (OR 6.1 [95% CI 1.01 to 36.7]; $p=0.049$) compared to those who reported "never" having food or groceries delivered. Exercising outdoors "daily" was associated with a lower risk of infection between March – May 2020 (OR 0.58 [95% CI 0.39 – 0.86]; $p=0.007$) and between June – July 2020 (OR 0.56 [95% CI 0.37 – 0.84[; $p=0.005$) compared to those who reported exercising outdoors less than once per week.

LASSO model fitting

The variables selected by the LASSO model are shown in **Table 3,** and included having a household member that had tested positive for SARS-CoV-2 antibodies or had had a positive SARS-CoV-2 PCR test, a household member previously displaying symptoms synonymous with COVID-19, Black ethnicity, working as a Physiotherapist, reporting working in acute medicine or medical subspecialities, reporting that adequate PPE was "rarely" available, working in a designated "Red" area, and attending handovers where adequate social distancing was not possible.

Multivariate analysis

We used a multivariate logistic regression model to include all variables selected by LASSO modelling. In this model, working in a designated "Red" area and having a household member with a

For peer review only - http://bmjopen.bmj.com/site/about/guidelines.xhtml

previous positive SARS-CoV-2 PCR swab were not significantly associated with the participant having a positive antibody test result (p>0.05), and were dropped from the final model. A total of eight variables were included in the final multivariate logistic regression model (Table 3; Figure 1).

In this resulting model the highest reported Odds Ratios associated with participants testing seropositive for SARS-CoV-2 antibodies were having a household member that had previous tested positive for SARS-CoV-2 antibodies (OR 6.94 [95% CI 4.15 – 11.6]; p<0.0001) and being of Black ethnicity (6.21 [95% CI 2.69 – 14.3]; p<0.0001). Occupational factors associated with a higher risk of seropositivity were working as a physiotherapist (OR 2.78 [95% CI 1.21 – 6.36]; p=0.015) and reporting that they predominantly worked in acute medicine (OR 2.72 [95% CI 1.57 – 4.69]; p<0.0001) or medical subspecialities (not including infectious diseases) (OR 2.33 [95% CI 1.4 – 3.88]; p=0.001). Reporting that adequate PPE was "rarely" available was associated with an OR of 2.83 (95% CI 1.29 – 6.25; p=0.01) and reporting attending a handover where social distancing was not possible was associated with an OR of 1.39 (95% CI 1.02 – 1.9; p=0.038).

DISCUSSION

In this systematic evaluation of demographic, occupational and behavioural risk factors associated with COVID-19 seropositivity amongst HCWs, we have identified several targetable risk factors for HCW infection during this, and future pandemics. The ability of healthcare systems to cope with surges of infections requiring hospitalisation has been challenged in a number of countries including the UK[20], India[21], USA[22] and Brazil[23] and resulted in excess deaths[22 24]. The resilience of a healthcare system relies heavily on staff remaining well and able to work. Healthcare workers have been disproportionately affected by infection rates[25 26] during this pandemic.

Both a positive SARS-CoV-2 antibody in a household member and prior symptoms in a household member were significantly associated with seropositivity in a multivariate model. The finding that a positive PCR test in a household member was not associated with seropositivity on multivariate analysis may reflect a proportional relationship between viral load and transmissibility in

For peer review only - http://bmjopen.bmj.com/site/about/guidelines.xhtml

asymptomatic infections. A study of Ct threshold values (as a proxy for viral load) in uncomplicated community SARS-CoV-2 demonstrated that self-reported symptoms were an independent predictor of lower Ct value (i.e. higher viral load), and that Ct values were significantly higher in those who remained antibody negative[27]. Taken together, these results suggest that a household member with positive symptoms (and either untested or false negative test) or a high enough viral load to develop antibodies contribute more to risk of infection in household members than a positive PCR test alone.

The finding that Black ethnicity remained highly significantly associated with seropositivity after controlling for many plausible explanations is concerning. The effect of increased risk of infection in certain ethnicities has been reported elsewhere; the reasons for this are complex and remain poorly understood but may include increased risk of household transmission[28]. South Asian and Black ethnicity have been found to be associated with a higher risk of hospitalisation, ICU admission and death relative to white ethnicity[29]. This finding suggests that occupational risk assessments should include ethnicity for HCWs from minority ethnic backgrounds.

The subjective feeling that adequate PPE was rarely available remained highly statistically significant in the final multivariate model. Whilst interesting, this finding requires a careful consideration of context and the subjective nature of the question. The availability and standard of PPE at CUH has been reported as exceeding that recommended by Public Health England for HCWs during the period of the study [30]. Furthermore, we have demonstrated elsewhere that the use of this enhanced PPE was effective at reducing the risk of infection amongst HCWs[30]. CUH reported the second-lowest number of hospital acquired COVID-19 cases in the East of England[31] out of 14 hospital trusts (suggesting high standards of infection control), with clinical outcomes for COVID-19 patients exceeding the national standard[32]. Despite these factors, 11% of staff reported the perception that adequate PPE was available "some of the time" or "rarely". Similar data are not available for comparison at other NHS sites. Staff at a higher risk of occupational exposure to infectious patients are likely to have experienced higher rates of anxiety related to PPE and therefore recall that anxiety, especially within the wider context of the media reporting of the national and global effects of the COVID-19 pandemic

For peer review only - http://bmjopen.bmj.com/site/about/guidelines.xhtml

during that time. This is demonstrated in the higher proportion of those reporting insufficient PPE being available “some of the time” or “rarely” working in COVID-19 red areas compared to those reporting adequate PPE being available “all of the time”. Nevertheless, the fact that this variable remained highly significant after LASSO variable selection and inclusion in the multivariate model highlights the need for availability of effective PPE for all HCWs at occupational risk of infection. Effective PPE is key for reducing infection, but also staff mental well-being and reducing potential burnout[33]. The impact of social distancing on the risk of COVID-19 infection is now well documented[34 35]. The practice of social distancing and mask wearing during shift-change handovers and other meeting times should continue to be encouraged as a modifiable behaviour that has the potential to decrease the risk of SARS-CoV-2 infection in HCWs.

The risk of hospitalisation with COVID-19 increases with age [36], and physiotherapists constitute an integral part of a multidisciplinary team during acute hospital admissions for elderly people [37]. In addition, physiotherapy played a key role in both ICU and acute medical wards with therapeutic positioning, early mobilisation and breathing exercises [38]. The increased risk of infection amongst physiotherapists during these activities requires further investigation and should be considered when assessing clinical practise risk and PPE standards.

These analyses have limitations. By their nature, questions about behavioural factors contain subjective answers, and must be interpreted with caution, including the subjective experience of availability of PPE. In addition, the questionnaire was sent to participants 3 -7 months following the period encompassed by the questions. This delay leaves responses open to recall bias, however most important factors assessed here (ethnicity, job role, prior household PCR and antibody results) are objective and are unlikely to have changed in the intervening period. Participants were aware of their serostatus at the time of completing the questionnaire, which may also have influenced responses to subjective questions, particularly around the availability of PPE. These analyses cover the time period where the original wild type Wuhan strain was the predominant circulating variant in the UK. Data on established and emerging variants, including the delta variant[4] and the now predominant Omicron

For peer review only - http://bmjopen.bmj.com/site/about/guidelines.xhtml

variant[6], suggest they may be more infectious and thus levels of risk and risk factors may not be identical. We think that the risk factors discussed within this paper are unlikely to be greatly affected by a change in the risk of infection in new variants and remain broadly generalisable as risk factors for HCW infection.

Our work identified a number of targetable risk factors for mitigation of the risk of HCW infection during the ongoing COVID-19 pandemic. Maintaining vigilance and providing adequate social distancing space for shift-change handover is likely to reduce the risk of HCW infection. The subjective experience of staff towards PPE should be considered when providing adequate and safe PPE provision and training. In addition, there are a number of non-modifiable risk factors, which nevertheless are feasible for extra mitigation strategies for healthcare professionals working within a health service to reduce the risk of HCW infection.

Acknowledgements

We thank NIHR BioResource volunteers for their participation, and gratefully acknowledge NIHR BioResource centres, NHS Trusts and staff for their contribution. We thank the National Institute for Health Research, NHS Blood and Transplant, and Health Data Research UK as part of the Digital Innovation Hub Programme. The views expressed are those of the author(s) and not necessarily those of the NHS, the NIHR or the Department of Health and Social Care.

Author Contributions

DJC, SL and SB conceived and designed the study. DJC, SB and SS conducted the analysis. DJC, SL, SB, NS, AS, HS, MF, PHM, JB, MPW and IG contributed to questionnaire design and analysis. Operational input and analysis was provided by AS and MF. Study logistics, questionnaire distribution and data collection were performed by Cambridge NIHR BioResource and the CITIID-NIHR BioResource COVID-19 collaboration consortium, overseen by HS. All authors read the manuscript and provided edits.

For peer review only - http://bmjopen.bmj.com/site/about/guidelines.xhtml

For peer review only

Funding

DJC and SL received funding for this work from Addenbrooke's Charitable Trust (Grant ID 900254). The work was also funded by awards from NIHR to the NIHR BioResource (RG94028 & RG85445). This research was funded in part by the Wellcome Trust [215515/Z/19/Z Senior Fellowship to SGB, 412 207498/Z/17/Z Senior Fellowship to IGG, 108070/Z/15/Z Senior Fellowhsip to MPW]. For the purpose of open access, the author has applied a CC BY public copyright licence to any Author Accepted Manuscript version arising from this submission.

Competing Interests

The authors declare no competing interests related to this study.

Data Sharing Statement

Data are available on reasonable request to the authors.

For peer review only

REFERENCES

1. WHO. WHO coronavirus disease (COVID-19) dashboard 2021 [Available from: https://covid19.who.int/ accessed June 07 2021.

2. Hyams C, Marlow R, Maseko Z, et al. Effectiveness of BNT162b2 and ChAdOx1 nCoV-19 COVID-19 vaccination at preventing hospitalisations in people aged at least 80 years: a test-negative, case-control study. *Lancet Infect Dis* 2021 doi: 10.1016/S1473-3099(21)00330-3 [published Online First: 2021/06/27]

3. Dagan N, Barda N, Kepten E, et al. BNT162b2 mRNA Covid-19 Vaccine in a Nationwide Mass Vaccination Setting. *The New England journal of medicine* 2021;384(15):1412-23. doi: 10.1056/NEJMoa2101765 [published Online First: 2021/02/25]

4. UK Health Security Agency. COVID-19 variants: genomically conformed case numbers [Available from: https://www.gov.uk/government/publications/covid-19-variants-genomically-confirmed-case-numbers accessed 08/02/2021 2021.

5. Frampton D, Rampling T, Cross A, et al. Genomic characteristics and clinical effect of the emergent SARS-CoV-2 B.1.1.7 lineage in London, UK: a whole-genome sequencing and hospital-based cohort study. *Lancet Infect Dis* 2021 doi: 10.1016/S1473-3099(21)00170-5 [published Online First: 2021/04/16]

6. Song JS, Lee J, Kim M, et al. Serial Intervals and Household Transmission of SARS-CoV-2 Omicron Variant, South Korea, 2021. *Emerging infectious diseases* 2022;28(3) doi: 10.3201/eid2803.212607 [published Online First: 2022/02/03]

7. Abu-Raddad LJ, Chemaitelly H, Butt AA, et al. Effectiveness of the BNT162b2 Covid-19 Vaccine against the B.1.1.7 and B.1.351 Variants. *The New England journal of medicine* 2021 doi: 10.1056/NEJMc2104974 [published Online First: 2021/05/06]

8. Emary KRW, Golubchik T, Aley PK, et al. Efficacy of ChAdOx1 nCoV-19 (AZD1222) vaccine against SARS-CoV-2 variant of concern 202012/01 (B.1.1.7): an exploratory analysis of a randomised controlled trial. *Lancet* 2021;397(10282):1351-62. doi: 10.1016/S0140-6736(21)00628-0 [published Online First: 2021/04/03]

For peer review only - http://bmjopen.bmj.com/site/about/guidelines.xhtml

9. Pajon R, Doria-Rose NA, Shen X, et al. SARS-CoV-2 Omicron Variant Neutralization after mRNA-1273 Booster Vaccination. *The New England journal of medicine* 2022 doi: 10.1056/NEJMc2119912 [published Online First: 2022/01/27]

10. Rossler A, Riepler L, Bante D, et al. SARS-CoV-2 Omicron Variant Neutralization in Serum from Vaccinated and Convalescent Persons. *The New England journal of medicine* 2022 doi: 10.1056/NEJMc2119236 [published Online First: 2022/01/13]

11. Nguyen LH, Drew DA, Graham MS, et al. Risk of COVID-19 among front-line health-care workers and the general community: a prospective cohort study. *Lancet Public Health* 2020;5(9):e475-e83. doi: 10.1016/S2468-2667(20)30164-X [published Online First: 2020/08/04]

12. NHS Digital. NHS Sickness Absence Rates, July 2021 to September 2021, Provisional Statistics. Published 3rd February 2022, 2022.

13. NHS England COVID-19 vaccination statistics. https://www.england.nhs.uk/statistics/statistical-work-areas/covid-19-vaccinations/. Accessed 01/03/2022.

14. Cooper DJ, Lear S, Watson L, et al. A prospective study of risk factors associated with seroprevalence of SARS-CoV-2 antibodies in healthcare workers at a large UK teaching hospital. *medRxiv* 2020:2020.11.03.20220699. doi: 10.1101/2020.11.03.20220699

15. Office for National Statistics. 2011 UK census. Available at: https://www.ons.gov.uk/census/2011census. Accessed 23rd September 2020.

16. National S-C-SAEG. Performance characteristics of five immunoassays for SARS-CoV-2: a head-to-head benchmark comparison. *Lancet Infect Dis* 2020 doi: 10.1016/S1473-3099(20)30634-4 [published Online First: 2020/09/27]

17. Royal College of Pathologists. Verification and validation methodology and sample sets for evaluation of assays for SARS-CoV-2 (COVID-19). Available here: https://www.rcpath.org/uploads/assets/541a4523-6058-4424-81c119dd2ab0febb/Verification-validation-of-sample-sets-assays-SARS-CoV-2.pdf. Accessed 23rd September 2020. .

18. Steyerberg EW, Eijkemans MJ, Harrell FE, Jr., et al. Prognostic modelling with logistic regression analysis: a comparison of selection and estimation methods in small data sets. *Stat Med*

For peer review only - http://bmjopen.bmj.com/site/about/guidelines.xhtml

2000;19(8):1059-79. doi: 10.1002/(sici)1097-0258(20000430)19:8<1059::aid-sim412>3.0.co;2-0 [published Online First: 2000/05/03]

19. Pavlou M, Ambler G, Seaman SR, et al. How to develop a more accurate risk prediction model when there are few events. *BMJ* 2015;351:h3868. doi: 10.1136/bmj.h3868 [published Online First: 2015/08/13]

20. Anderson M, Pitchforth E, Asaria M, et al. LSE-Lancet Commission on the future of the NHS: re-laying the foundations for an equitable and efficient health and care service after COVID-19. *Lancet* 2021;397(10288):1915-78. doi: 10.1016/S0140-6736(21)00232-4 [published Online First: 2021/05/10]

21. The L. India's COVID-19 emergency. *Lancet* 2021;397(10286):1683. doi: 10.1016/S0140-6736(21)01052-7 [published Online First: 2021/05/10]

22. Woolf SH, Chapman DA, Sabo RT, et al. Excess Deaths From COVID-19 and Other Causes in the US, March 1, 2020, to January 2, 2021. *JAMA* 2021 doi: 10.1001/jama.2021.5199 [published Online First: 2021/04/03]

23. Lemos DRQ, D'Angelo SM, Farias L, et al. Health system collapse 45 days after the detection of COVID-19 in Ceara, Northeast Brazil: a preliminary analysis. *Rev Soc Bras Med Trop* 2020;53:e20200354. doi: 10.1590/0037-8682-0354-2020 [published Online First: 2020/07/09]

24. Kontopantelis E, Mamas MA, Webb RT, et al. Excess deaths from COVID-19 and other causes by region, neighbourhood deprivation level and place of death during the first 30 weeks of the pandemic in England and Wales: A retrospective registry study. *Lancet Reg Health Eur* 2021;7:100144. doi: 10.1016/j.lanepe.2021.100144 [published Online First: 2021/09/25]

25. Nguyen LH, Drew DA, Joshi AD, et al. Risk of COVID-19 among frontline healthcare workers and the general community: a prospective cohort study. *medRxiv* 2020 doi: 10.1101/2020.04.29.20084111 [published Online First: 2020/06/09]

26. Shah ASV, Wood R, Gribben C, et al. Risk of hospital admission with coronavirus disease 2019 in healthcare workers and their households: nationwide linkage cohort study. *BMJ* 2020;371:m3582. doi: 10.1136/bmj.m3582 [published Online First: 2020/10/30]

For peer review only

For peer review only - http://bmjopen.bmj.com/site/about/guidelines.xhtml

27. Walker AS, Pritchard E, House T, et al. Ct threshold values, a proxy for viral load in community SARS-CoV-2 cases, demonstrate wide variation across populations and over time. *Elife* 2021;10 doi: 10.7554/eLife.64683 [published Online First: 2021/07/13]

28. Morales DR, Ali SN. COVID-19 and disparities affecting ethnic minorities. *Lancet* 2021;397(10286):1684-85. doi: 10.1016/S0140-6736(21)00949-1 [published Online First: 2021/05/04]

29. Mathur R, Rentsch CT, Morton CE, et al. Ethnic disparities in COVID-19: increased risk of infection or severe disease? - Authors' reply. *Lancet* 2021;398(10298):390. doi: 10.1016/S0140-6736(21)01424-0 [published Online First: 2021/08/02]

30. Ferris M, Ferris R, Workman C, et al. Efficacy of FFP3 respirators for prevention of SARS-CoV-2 infection in healthcare workers. *Elife* 2021;10 doi: 10.7554/eLife.71131 [published Online First: 2021/11/17]

31. Cambridge University Hospital NHS Foundation Trust. Infection Control Anuual Report 2020-2021. https://buckup-cuh-production.s3.amazonaws.com/documents/Infection_Control_Annual_Report_2020-21_final_SaUbrqH.pdf, 2021.

32. Gray WK, Navaratnam AV, Day J, et al. Variability in COVID-19 in-hospital mortality rates between national health service trusts and regions in England: A national observational study for the Getting It Right First Time Programme. *EClinicalMedicine* 2021;35:100859. doi: 10.1016/j.eclinm.2021.100859 [published Online First: 2021/05/04]

33. Ferry AV, Wereski R, Strachan FE, et al. Predictors of UK healthcare worker burnout during the COVID-19 pandemic. *QJM* 2021;114(6):374-80. doi: 10.1093/qjmed/hcab065 [published Online First: 2021/03/27]

34. Kwon S, Joshi AD, Lo CH, et al. Association of social distancing and face mask use with risk of COVID-19. *Nat Commun* 2021;12(1):3737. doi: 10.1038/s41467-021-24115-7 [published Online First: 2021/06/20]

35. Chu DK, Akl EA, Duda S, et al. Physical distancing, face masks, and eye protection to prevent person-to-person transmission of SARS-CoV-2 and COVID-19: a systematic review and

For peer review only

meta-analysis. *Lancet* 2020;395(10242):1973-87. doi: 10.1016/S0140-6736(20)31142-9 [published Online First: 2020/06/05]

36. Dashti H, Roche EC, Bates DW, et al. SARS2 simplified scores to estimate risk of hospitalization and death among patients with COVID-19. *Sci Rep* 2021;11(1):4945. doi: 10.1038/s41598-021-84603-0 [published Online First: 2021/03/04]

37. Ellis G, Sevdalis N. Understanding and improving multidisciplinary team working in geriatric medicine. *Age Ageing* 2019;48(4):498-505. doi: 10.1093/ageing/afz021 [published Online First: 2019/03/12]

38. Thomas P, Baldwin C, Bissett B, et al. Physiotherapy management for COVID-19 in the acute hospital setting: clinical practice recommendations. *J Physiother* 2020;66(2):73-82. doi: 10.1016/j.jphys.2020.03.011 [published Online First: 2020/04/22]

For peer review only

Table 1: Participant characteristics

Baseline variable	n / N [responses] (%)
Sex (male)	400/2044 (20)
Age (years), median (IQR)	42 (32 – 53)
Ethnicity	
- White British	1584 (70)
- White Irish	35 (1.6)
- White (other)	294 (13)
- Asian or Asian British (Indian)	70 (3.1)
- Asian or Asian British (Pakistani)	8 (0.4)
- Asian or Asian British (Bangladeshi)	2 (0.1)
- Asian or Asian British – Other	116 (5.1)
- Black or Black British (caribbean)	7 (0.3)
- Black or Black British (African)	29 (1.3)
- Black or Black British (Other)	1 (0.04)
- Mixed – White and Black Caribbean	6 (0.3)
- Mixed – White and Black African	9 (0.4)
- Mixed – White and Asian	12 (0.5)
- Mixed – Other	10 (0.4)
- Chinese	27 (1.2)
- Any other ethnic group	26 (1.2)
- Not stated	19 (0.84)
COVID working	618 / 2235 (28)

For peer review only - http://bmjopen.bmj.com/site/about/guidelines.xhtml

Table 2 – Significant Univariate analysis variables

	Variable	Unadjusted Odds Ratio	95 % CI	p-value	n (positive) / N (responses) (%)
**Demographic**					
	Rent room in shared house	1.84	1.22 – 2.74	0.003	33/209 (16)
	Live with other HCWs	1.49	1.10 – 2.02	0.009	70/550 (13)
	Children attended school in June	0.58	0.35 – 0.97	0.038	30/395 (7.6)
	Household member positive PCR test	3.48	2.09 – 5.78	<0.0001	22/84 (26)
	Household member positive Ab test	11.29	7.08 – 18.01	<0.0001	40/79 (51)
	Household member symptomatic	3.71	2.8 – 4.96	<0.0001	95/437 (22)
	Born in UK	0.59	0.44 – 0.79	<0.001	136/1616 (8.4)
	Ethnicity	1.06	1.03 – 1.10	<0.001[a]	1584/2258[b]
**Occupational**					
	Job role				
	- Admin staff	1	-	-	24/336 (7)
	- Staff nurse	2.02	1.18 – 3.43	0.01	40/298 (13.4)
	- Physiotherapist	4.33	1.83 – 10.25	0.001	9/36 (25)
	Direct patient care COVID	1.86	1.41 – 2.47	<0.001	103/757 (13.6)

For peer review only - http://bmjopen.bmj.com/site/about/guidelines.xhtml

Worked in red area	1.78	1.33 – 2.38	<0.001	85/618 (13.8)
Specialty				
- Non-patient facing roles	1	-	-	10/169 (6)
- Critical care	2.51	1.10 – 5.77	0.029	16/117 (13.7)
- Acute med	4.57	2.08 – 10.07	<0.001	23/103 (22.3)
- Medical specialties	4.35	2.01 – 9.42	<0.001	26/121 (21.5)
- Surgical	2.71	1.24 – 5.93	0.012	22/151 (14.6)
Night shifts	1.68	1.26 – 2.25	<0.001	82/604 (13.6)
Receive formal PPE training	1.40	1.05 – 1.85	0.02	129/1141 (11.3)
Adequate PPE available				
- All of the time	1	-	-	83/1038 (8)
- Most of the time	1.34	0.98 – 1.83	0.065	92/882 (10.4)
- Some of the time	1.93	1.22 – 3.05	0.005	28/195 (14.4)
- Rarely	3.60	1.71 – 7.57	0.001	10/42 (23.8)
Rest/meal with colleagues				
- Never	1	-	-	
- Most of the time	1.99	1.19 – 3.33	0.009	(compared to "Never")

For peer review only - http://bmjopen.bmj.com/site/about/guidelines.xhtml

	Use doctors mess	1.77	1.17 – 2.69	0.007	30/195 (15.4)
	Hospital supplied scrubs	1.15	1.04 – 1.27	0.007	
	Work from home - March	0.60	0.39 – 0.91	0.016	
	Work from home – June	0.58	0.36 – 0.94	0.026	
	Handover w/o social distancing	1.74	1.31 – 2.30	<0.0001	
**Behavioural**					
	Smoker	0.37	0.18 – 0.76	0.007	
	Food deliveries march				Compared to < 1 /week
	- Daily	5.38	1.27 – 22.8	0.022	
	Food deliveries June				
	- Daily	6.10	1.01 – 36.7	0.049	Compared to < 1 /week
	Exercise outdoors March				
	- Daily	0.58	0.39 – 0.86	0.007	Compared to < 1 /week
	Exercise outdoors June				
	- Daily	0.56	0.37 – 0.84	0.005	

[a] p-value for likelihood ratio test

[b] number of participants identifying as white British

For peer review only - http://bmjopen.bmj.com/site/about/guidelines.xhtml

Table 3 – Final multivariate model

Variable	OR	95% CI	p-value
Household positive antibody	6.94	4.15 – 11.6	<0.001
Household positive symptoms	2.95	2.13 – 4.08	<0.001
Black ethnicity	6.21	2.69 – 14.3	<0.001
Physiotherapist	2.78	1.21 – 6.39	0.015
Acute medicine specialty	2.72	1.57 – 4.69	<0.001
Medical specialties	2.33	1.40 – 3.88	0.001
Inadequate PPE	2.84	1.29 – 6.25	0.010
Handover w/o distancing	1.39	1.02 – 1.90	0.038

For peer review only

Figure 1: Forest plot of final multivariate model.

For peer review only

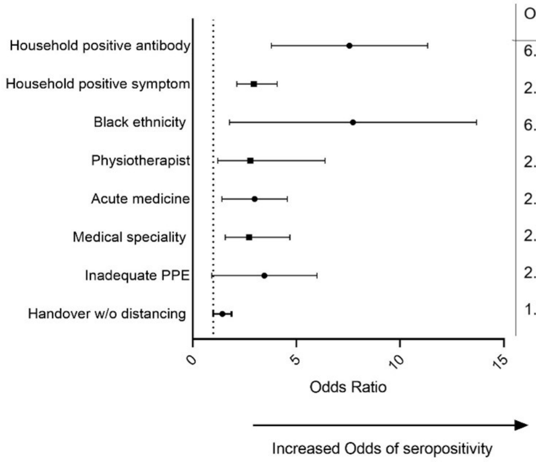

OR	95% CI	p-value
6.94	4.15 – 11.6	<0.001
2.95	2.13 – 4.08	<0.001
6.21	2.69 – 14.3	<0.001
2.78	1.21 – 6.39	0.015
2.72	1.57 – 4.69	<0.001
2.33	1.40 – 3.88	0.001
2.84	1.29 – 6.25	0.010
1.39	1.02 – 1.90	0.038

Figure 1: Forest plot of final multivariate model.

159x80mm (220 x 220 DPI)

A. Demographics

1. How many people live in your household (select number)
2. Do you rent a room in a shared house (yes/no)
3. Do you live with other healthcare workers (yes/no)
4. Do you live with other key workers (who are not healthcare workers) who have worked during this time? (yes/no)
5. Is there more than one generation of your family living in your household (e.g. Children, parents or grandparents) (yes/no) If yes (may be multiple):

- Children
- Parents
- Grandparents
- More than one of the above
- Other (free text)

6. Are there children living in your house? (yes/no)

- If yes, how many.
- What ages (select from drop down list for each child – multiple depending on how many entered in answer above if possibly [– *on REDCap, or provide boxes for paper form*])

7. Do you have school aged children? (yes/no). If yes:

- Did they attend school between March to May 2020? (yes/no)
- Did they attend school between from June to July 2020? (yes/no)

8. Do you have children who attend nursery. (yes/no). If yes:

- Did they attend nursery between March to May 2020? (yes/no)
- Did they attend nursery between from June to July 2020? (yes/no)

9. Is there anyone in your household who is >65 years old? (yes/no)
10. Did anybody in your household (other than yourself) test positive on a throat swab (PCR test) for COVID-19?
11. Did anybody in your household (other than yourself) test positive on a blood test (antibody test) for COVID-19?
12. Did anybody (other than yourself) have symptoms consistent with COVID-19 between February and July 2020?
13. How do you travel to and from work? (drop down list)

- Walk/run; cycle; personal car; bus; train

14. If you drive, do you share lifts with other healthcare workers who aren't in your immediate household? (yes/no)

B. Socioeconomic

1. Were you born in the United Kingdom? (yes/no)

For peer review only

2. Ethnicity (drop down list) *[– insert List of NHS ethnicity codes A-Z]*
3. What is your highest level of education? (Select from list: GCSE; A level; Undergraduate degree; higher degree; other vocational training)
4. Have you been employed in more than one job during this time? (yes/no)
5. Do you have any dependents other than your immediate family members? (yes/no)
6. Do you provide care for anyone outside of your immediate household? (including washing/dressing, cooking, shopping, cleaning, healthcare needs) (yes/no)

C. Occupational

1. What is your job role?

- Admin or reception staff
- Staff nurse
- Nursing Sister / Senior nursing staff
- Consultant
- Junior doctor (including FY1/FY2/Core trainee/Speciality Trainee)
- Laboratory staff
- Healthcare Assistant
- Operating department staff
- Manager
- Radiographer
- Midwife
- Physiotherapist / Physiotherapy assistant
- Pharmacy staff
- Cleaning/domestic staff
- Dietician
- Occupational therapist
- SALT
- Porter
- Other (FREE TEXT)

2. Please select all areas you have worked during this time (may be multiple):

- Ward A2 - Neurosciences critical care unit (NCCU)
- Ward J2 - Trauma high dependency unit
- Ward A4 - Neurology / Neurosurgery
- Ward A5 - Neuro-oncology / Neurosurgery
- Ward C2 - Children's oncology and haematology
- Ward C3 - Children's surgical and medicine
- Ward C4 - Frail and Acute Medicine for the Elderly
- Ward C5 - General medicine and nephrology
- Ward C6 - Medicine for the elderly
- Ward C7 - Gastroenterology
- Ward C8 - Surgical Admissions for 'Amber' patients

For peer review only

- Ward C9 - Teenage Cancer Trust Unit
- Ward C10 - Haematology and haematological oncology
- Ward D2 - Children's surgical and medicine
- Ward D3 - John Farman intensive care unit
- Ward D4 - Intermediate dependancy area
- Ward D5 – DME Medicine for the elderly
- Ward D6 - Neuro/Stroke/ Neurosurgery/Gastro Haematology
- Ward D7 - Diabetes and endocrinology
- Ward D9 - Oncology
- Ward D10 - Respiratory
- Ward EAU 2 - Paediatric Emergency Department
- Ward EAU 3 - Ambulatory care
- Ward EAU 4 - Acute Hub - Green Medical Admissions/Short Stay
- Ward EAU 5 - Acute Hub - Red Medicine
- Ward F2 - Inpatient Occupational Therapy
- Ward F3
- Ward F4 - Renal
- Ward F5 - Transplant high dependency unit
- Ward F6 - Trauma and Orthopaedics
- Ward G2 - Infusion services
- Ward G3 - Diabetes, I.D. and Oncology
- Ward G4 - Hepatology
- Ward G5 - Transplant unit
- Ward G6 - Medicine for the elderly
- Ward J2 - Major trauma unit
- Ward J3 - Post Anaesthetic Care Unit (PACU) and 23 Hour Stays
- Ward K2 - Cardiology
- Ward K3 - Cardiology and coronary care unit
- Ward L2 - Day surgery unit
- Ward L4 - Non-Elective Surgery Patients
- Ward L5 - Non-Elective Surgery Patients
- Ward M4 - Non-Elective Surgery Patients
- Ward M5 - Elective Surgery Patients
- Ward N2 -Amber Medical Admissions for Covid Pathway
- Ward N3 - Respiratory medicine
- Ward R3 - Neurosciences
- Ward S3 - Psychiatry
- Surgical Ambulatory Care Unit
- Clinical Investigation Ward (CIW)
- Clinical Research Facility (CRF)
- Coronary care unit (CCU)
- Haematology day unit
- Intermediate dependency area (IDA)
- Ward EAU 4 - Acute Hub - Green Medical Admissions/Short Stay
- Paediatric intensive care unit (PICU)
- Paediatric Day Unit (PDU)
- Stroke Unit - Ward R2 and Lewin rehabilitation unit
- Delivery unit

For peer review only

- Ward - Lady Mary - Postnatal
- Neonatal unit
- Ward - Sara - Antenatal
- Daphne ward – Gynaecology
- Ward - Charles Wolfson

3. Have you been involved in the direct patient care of patients with confirmed COVID-19? (yes/no)

4. Have you worked in a specified "Red" area between March and July 2020? (yes/no). If yes:

- Less than 1 week
- 1 week
- 1 week – 1 month
- >1 month

5. Which speciality have you predominantly worked in between March and July 2020?

- Emergency Department
- Critical Care
- Acute Medicine
- Respiratory Medicine
- Infectious Diseases
- Medicine (not including Respiratory or Infectious Diseases)
- Operating Department (Theatres)
- ENT
- Surgical specialties
- Paediatrics
- Research
- Non-patient facing role

6. How many hours did you work in the average week from March to May 2020?

7. How many hours did you work in the average week from June to July 2020?

8. Does your working pattern include night shifts? (yes/no)

9. Have you been present during aerosol generating procedures on COVID-19 confirmed patients? (yes/no). If yes:

- tracheal intubation and extubation
- manual ventilation
- tracheotomy or tracheostomy procedures (insertion or removal)
- bronchoscopy
- dental procedures (using high speed devices, for example ultrasonic scalers/high speed drills
- non-invasive ventilation (NIV); Bi-level Positive Airway Pressure Ventilation (BiPAP) and Continuous Positive Airway Pressure Ventilation (CPAP)
- high flow nasal oxygen (HFNO)
- high frequency oscillatory ventilation (HFOV)
- induction of sputum using nebulised saline

For peer review only

- respiratory tract suctioning
- upper ENT airway procedures that involve respiratory suctioning
- upper gastro-intestinal endoscopy where open suction of the upper respiratory tract occurs
-

10. Did you receive formal PPE training? (yes/no)

11. Did you feel that adequate PPE was available to you:

- At all times
- Most of the time
- Some of the time
- Rarely

12. Prior to the introduction of hospital-wide surgical-resistant masks, which type of facemask did you predominantly use at work?

- None
- Water resistant surgical mask
- FFP3
- Respirator hood
- Other respirator
- Other

13. After the introduction of hospital-wide surgical-resistant masks, which type of facemask did you predominantly use at work?

- None
- Water resistant surgical mask
- FFP3
- Respirator hood
- Other respirator
- Other

14. What type of eye protection did you predominantly use at work:

- None
- Own spectacles/glasses
- Protective glasses (hospital supplied)
- Goggles
- Face shield

15. Did you take rest/meal breaks at the same time as colleagues?

- All of the time
- Most of the time
- Some of the time
- Rarely
- Never

16. Did you eat in the staff canteen?

- All of the time
- Most of the time

For peer review only

- Some of the time
- Rarely
- Never

17. Did you use shared rest facilities in your primary area of work (e.g. tea/break room)?

- All of the time
- Most of the time
- Some of the time
- Rarely
- Never

18. Did you use the doctors' mess during this time?

- All of the time
- Most of the time
- Some of the time
- Rarely
- Never

19. Did you wear hospital supplied scrubs at work?

- All of the time
- Most of the time
- Some of the time
- Rarely
- Never

20. Did you wear your own scrubs at work?

- All of the time
- Most of the time
- Some of the time
- Rarely
- Never

21. Did you wear your own clothes to work?

- All of the time
- Most of the time
- Some of the time
- Rarely
- Never

22. Did you use a changing room at work?

- All of the time
- Most of the time
- Some of the time
- Rarely
- Never

23. Did you have dedicated footwear for work during this time?

For peer review only

- All of the time
- Most of the time
- Some of the time
- Rarely
- Never

24. Did you wear your work clothes when leaving the hospital?

- All of the time
- Most of the time
- Some of the time
- Rarely
- Never

25. Did you use a reusable personal water/drinks bottle in your area of work?

- All of the time
- Most of the time
- Some of the time
- Rarely
- Never

26. How would you rate your adherence to trust policy hand-washing technique?

- All of the time
- Most of the time
- Some of the time
- Rarely
- Never

27. How would you rate your adherence to trust policy hand-washing frequency

- All of the time
- Most of the time
- Some of the time
- Rarely
- Never

28. Did you primarily work from home between March to May 2020?

- If yes, was this recommended for shielding reasons?

29. Did you primarily work from home from June to July 2020?

- If yes, was this recommended for shielding reasons?

30. Have you ever been recommended to shield by Occupational Health?

31. Have you ever been in a group that was recommended to shield by Public Health England? (yes/no)

D. Behavioural

For peer review only

1. Were you a smoker at any point between March to July 2020? (yes/no) If yes:

- Fewer than 5 per day
- 5-10 per day
- 10-20 per day
- >20 per day

2. Did you regularly drink alcohol between March to July 2020? (yes/no) If yes:

- Daily
- 2-3 times per week
- Once a week
- Less than once a week

3. How frequently did you visit a supermarket or shop between March to May 2020?

- Daily
- 2-3 times per week
- Once a week
- Less than once a week

4. How frequently did you visit a supermarket or shop between June to July 2020?

- Daily
- 2-3 times per week
- Once a week
- Less than once a week

5. How often did you have contact with people outside of your immediate household (not including work) between March to May 2020?

- Daily
- 2-3 times per week
- Once a week
- Less than once a week

6. How often did you have contact with people outside of your immediate household (not including work) between June to July 2020?

- Daily
- 2-3 times per week
- Once a week
- Less than once a week

7. How often did you order food deliveries (e.g. groceries, take-away) between March to May 2020?

- Daily
- 2-3 times per week
- Once a week

For peer review only

- Less than once a week

8. How often did you order food deliveries (e.g. groceries, take-away) between June to July 2020?

- Daily
- 2-3 times per week
- Once a week
- Less than once a week

9. How often did you exercise outdoors from March to May 2020?

- Daily
- 2-3 times per week
- Once a week
- Less than once a week

10. How often did you exercise outdoors June to July 2020?

- Daily
- 2-3 times per week
- Once a week
- Less than once a week

11. How often did you use public transport (not including travel to and from work) from March to May 2020?

- Daily
- 2-3 times per week
- Once a week
- Less than once a week

12. How often did you use public transport (not including travel to and from work) from June to July 2020?

- Daily
- 2-3 times per week
- Once a week
- Less than once a week

13. Did you use a facemask outside of work from March to May 2020?

- All of the time
- Most of the time
- Some of the time
- Rarely
- Never

14. Did you use a facemask outside of work from June to July 2020?

For peer review only

- All of the time
- Most of the time
- Some of the time
- Rarely
- Never

15. If you used a facemask outside of work, in which situations did you use one? (may be multiple)

- Social interaction
- Grocery shopping
- Commuting
- Exercising
- Other

16. Did you attend meetings or handovers where it was not possible to socially distance between March to May 2020? (yes/no)

17. Did you attend meetings or handovers where it was not possible to socially distance between June to July 2020? (yes/no)

E. Co-morbidities

1. What was your COVID risk-assessment group?

- Green
- Yellow
- Orange
- Red

2. Were your work duties altered because of your risk group? (yes/no)

3. Self-reported height [give measuring unit options ft/inches or m/cm]

4. Self-reported weight [give measuring usingt options st/lb or kg]

5. Have you even been told you are overweight in a medical setting? (yes/no)

6. Have you even been told you are obese in a medical setting? (yes/no

7. How often do you undertake physical exercise?

- Daily
- 2-3 times per week
- Once a week
- Less than once a week

8. Do you have any of the following co-morbidities:

Heart disease. (yes/no) If yes – select (may be multiple)

- Ischaemic heart disease
- Previous myocardial infarction (heart attack)
- Angina
- Valvular heart disease

For peer review only

- Other

Kidney disease. (yes/no) If yes – select

- Chronic kidney disease – not on dialysis
- Are you on haemodialysis?
- Are you on Peritoneal dialysis?
- Have you had a kidney transplant?
- Vasculitis
- Other

Lung disease. (yes/no) If yes – select (may be multiple)

- Chronic obstructive pulmonary disease (COPD)/ Chronic obstructive airway disease (COAD)
- Asthma
- Interstitial lung disease
- Bronchiectasis
- Emphysema
- Other

Have you ever been diagnosed with high blood pressure (yes/no). If yes:

- Are you on any medication. (yes/no)
- How many different medications (insert number)
- Is your blood pressure well controlled? (yes/no)

Type 1 diabetes (yes/no)

Type 2 diabetes (yes/no). If yes:

- Do you take insulin?
- How many medications do you take for diabetes? (must include zero)
- Is your blood sugar well controlled?

Do you have a compromised immune system due to any of the following?

- Immunosuppression drugs (yes/no)
- Blood disorder (including blood cancer) (yes/no)
- An inherited immune deficiency (yes/no)
- Other (free text) (yes/no)

Have you had a solid organ transplant (yes/no). If yes:

- Kidney
- Heart
- Lung
- Liver
- Small intestine
- Pancreas

Are you currently being treated for cancer? (yes/no). If yes:

- Solid organ cancer
- Blood cancer

For peer review only

- Skin cancer
- Other

9. Have you taken hydroxychloroquine at any time between March to July 2020? (yes/no). If yes:

- More than once daily
- Once daily
- 2 – 6 times a week
- Once a week
- Less than once a week

10. Did you take any of the following medication between March and July 2020 (may be multiple):

- Aspirin
- Angiotensin converting enzyme (ACE) inhibitors (including ramipril, lisinopril, captopril, enalopril and others)
- Angiotensin receptor blockers (ARBs) (including candesartan, irbesartan, losartan, valsartan and others)
- Tacrolimus
- Mycophenolate
- Hydroxychloroquine
- Prednisolone
- Tocilizumab
- Azathioprine
- Methotrexate
- Cyclosporine
- Leflunomide

11. Have you ever had any of the following medication (may be multiple):

- Rituximab
- Abatacept
- Adalimumab
- Etanercept
- Infliximab
- Basiliximab
- Cyclophosphamide

12. Have you ever had chemotherapy for cancer? (yes/no)

13. Have you ever had immunosuppressive medication not listed in the above questions? (yes/no). If yes:

- Free text

For peer review only

Univariate logistic regression tables of variables assessed

A. *Demographic factors and association with risk of SARS-CoV-2 infection in HCWs.*

**Variable**	**OR**	**95 % CI**	***p*-value**	n (positive) / N (responses) (%)
Number in househould (1)	1	-	-	14/217 (6.5)
- 2	1.64	0.91 – 2.96	0.10	78/768 (10.1)
- 3	1.43	0.76 – 2.68	0.26	42/468 (9.0)
- 4	1.59	0.86 – 2.92	0.14	55/557 (9.9)
- 5	2.43	0.97 – 4.69	0.09	19/160 (11.9)
- 6	2.11	0.81 – 5.52	0.13	7/55 (12.7)
- 7	0.81	0.10 – 6.48	0.84	1/19 (5.2)
- 8	4.8	0.47 – 49.5	0.19	1/4 (25)
- 9	1	-	-	0/5 (0)
Rent room in shared house	1.84	1.22 – 2.74	0.003	33/209 (16)
Live with other HCWs	1.49	1.10 – 2.02	0.009	70/550 (13)
Live other key workers (not HCWs)	0.95	0.70 – 1.29	0.73	63/663 (9.5)

For peer review only

Multigenerational household	0.96	0.73 – 1.27	0.79	104/1073 (9.7)
Children in household	1.13	0.85 – 1.49	0.40	99/944 (10.5)
Number of children				
- 0	1	-	-	126/1331 (9.5)
- 1				
- 2	1.04	0.72 – 1.50	0.84	43/439 (9.8)
- 3	1.28	0.70 – 2.35	0.42	13/110 (11.8)
- 4	1.91	0.41 – 8.83	0.41	2/12 (16.7)
- 5	2.39	0.27 – 21.6	0.44	1/5 (20)
School aged children	0.97	0.72 – 1.32	0.86	66/684 (9.7)
Children attended school in March	1.52	0.90 – 2.56	0.12	27/225 (12)
Children attended school in June	0.58	0.35 – 0.97	0.038	30/395 (7.6)
Nursery age children	0.69	0.38 – 1.23	0.20	13/183 (7.1)
Children attend nursery March	1.91	0.56 – 6.51	0.30	6/72 (8.3)
Children attend nursery June	1.05	0.31 – 3.55	0.94	9/125 (7.2)
People >65 in household	1.05	0.65 – 1.68	0.86	21/207 (10.1)
Household member positive PCR test	3.48	2.09 – 5.78	<0.0001	22/84 (26)

For peer review only

Household member positive Ab test	11.29	7.08 – 18.01	<0.0001	40/79 (51)
Household member symptomatic	3.71	2.8 – 4.96	<0.0001	95/437 (22)
Travel to work				
- Drive	1	-	-	133/1449 (9.2)
- Walk	1.28	0.78 – 2.10	0.34	20/175 (11.4)
- Cycle	1.19	0.84 – 1.69	0.33	47/438 (10.7)
- Bus	1.15	0.63 – 2.10	0.65	13/125 (10.4)
- Train	1.62	0.75 – 3.48	0.22	8/57 (14.0)
Share a car	1.49	0.84 – 2.61	0.17	15/109 (13.8)

For peer review only

B. Socioeconomic *factors and association with risk of SARS-CoV-2 infection in HCWs.*

**Variable**	**OR**	**95 % CI**	***p*-value**	n (positive) / N (responses) (%)
Born in UK	0.59	0.44 – 0.79	<0.001	136/1616 (8.4)
Ethnicity	1.06	1.03 – 1.10	<0.001[a]	1584/2258[b]
Highest level of education				
- Higher degree	1	-	-	84/869 (9.7)
- GCSE	1.02	0.61 – 1.71	0.94	20/203 (9.9)
- A level	0.90	0.54 – 1.51	0.70	20/227 (8.8)
- Undergraduate degree	1.15	0.83 – 1.59	0.40	79/722 (10.9)
- Other vocational training	0.75	0.44 – 1.30	0.31	17/228 (7.5)
More than one job	0.95	0.61 – 1.49	0.84	24/254 (9.5)
Other dependents	1.21	0.63 – 2.30	0.57	11/95 (11.6)
Care outside of household	0.57	0.34 – 0.11	0.056	19/232 (8.2)

[a] p-value for likelihood ratio test

[b] number of participants identifying as white British

For peer review only

C. Occupational factors and association with risk of SARS-CoV-2 infection in HCWs.

Variable	OR	95 % CI	p-value	n (positive) / N (responses) (%)
Job role				
- Administrative staff	1	-	-	24/336 (7.1)
- Staff nurse	2.02	1.18 – 3.43	0.01	40/298 (13.4)
- Senior nursing staff	1.54	0.89 – 2.67	1.55	33/311 (10.6)
- Consultant	1.66	0.86 – 3.19	0.13	17/150 (11.3)
- Junior doctor	1.67	0.77 – 3.63	0.20	10/88 (11.4)
- Laboratory staff	0.58	0.23 – 1.44	0.24	6/141 (4.3)
- Healthcare assistant	1.71	0.93 – 3.15	0.08	22/189 (11.6)
- Theatre staff	0.54	0.07 – 4.18	0.56	1/25 (4)
- Manager	1.75	0.87 – 3.51	0.12	14/118 (11.9)
- Radiographer	1.39	0.54 – 3.56	0.69	6/62 (9.7)
- Midwife	0.20	0.27 – 1.53	0.12	1/65 (1.5)

For peer review only

- Physio	4.33	1.83 – 10.25	0.001	9/36 (25)
- Pharmacy staff	2.07	0.84 – 5.08	0.11	7.51 (13.7)
- Cleaning/domestic staff	1	-	-	0/6 (0)
- Dietician	0.59	0.076 – 4.57	0.61	1/23 (4.4)
- Occupational therapist	0.87	0.11 – 6.84	0.89	1/16 (6.25)
- Speech and Language therapist	2.29	0.63 – 8.38	0.48	3/20 (15)
- Porter	2.17	0.25 – 18.7	0.48	1/7 (14.3)
- Other	1.17	0.05 – 0.12	0.59	26/314 (8.3)
Direct patient care COVID	1.86	1.41 – 2.47	<0.001	103/757 (13.6)
Worked in red area	1.78	1.33 – 2.38	<0.001	85/618 (13.8)
Time in red area	0.99	0.83 – 1.20	0.99	-
Specialty				
- Non-patient facing	1	-	-	10/169 (5.9)
- Emergency department	1.32	1.10 – 5.77	0.44	44/574 (7.7)
- Critical care	2.51	1.10 – 5.77	0.029	16/117 (13.7)
- Acute medicine	4.57	2.08 – 10.07	<0.001	23/103 (22.3)
- Respiratory medicine	2.0	0.51 – 7.74	0.32	3/27 (11.1)

For peer review only

- Infectious diseases	1.59	0.32 – 7.78	0.57	2/22 (9.1)
- Medical specialties	4.35	2.01 – 9.42	<0.001	26/121 (21.5)
- Theatres	2.01	0.80 – 5.04	0.14	10/89 (11.2)
- ENT	0.66	0.08 – 5.41	0.70	1/25 (4)
- Surgical	2.71	1.24 – 5.93	0.012	22/151 (14.6)
- Paediatrics	0.71	0.24 – 2.13	0.54	5/117 (4.3)
- Research	1.44	0.64 – 3.25	0.37	17/204 (8.3)
- Other	1.27	0.61 – 2.66	0.53	11/101 (10.9)
Average hours per week March	1.02	0.96 – 1.09	0.49	-
Average hours per week June	0.99	0.93 – 1.06	0.84	-
Work nights	1.68	1.26 – 2.25	<0.001	82/604 (13.6)
Present for AGPs	1.30	0.93 – 1.84	0.13	47/396 (11.9)
Receive formal PPE training	1.40	1.05 – 1.85	0.02	129/1141 (11.3)
Adequate PPE available				
- All of the time	1	-	-	83/1038 (8.0)
- Most of the time	1.34	0.98 – 1.83	0.065	92/882 (10.4)
- Some of the time	1.93	1.22 – 3.05	0.005	28/195 (14.4)

For peer review only

- Rarely	3.60	1.71 – 7.57	0.001	10/42 (23.8)
Use mask at work before widespread	0.98	0.86 – 1.12	0.79	127/1198 (10.6)
Which mask when mandatory	0.94	0.81 – 1.10	0.45	-
What type of eye protection	1.05	0.97 – 1.14	0.25	-
Rest/meal with colleagues				
- Never	1	-	-	21/297 (7.1)
- All of the time	1.49	0.81 – 2.76	0.20	24/235 (10.2)
- Most of the time	1.99	1.19 – 3.33	0.009	64/487 (13.1)
- Some of the time	1.52	0.92 – 2.51	0.10	76/733 (10.4)
- Rarely	1.05	0.60 – 1.85	0.86	35/472 (7.4)
Eat in staff canteen	1.08	0.99 – 1.17	0.06	-
Shared rest areas	0.99	0.90 – 1.11	0.96	-
Use doctors mess	1.77	1.17 – 2.69	0.007	30/195 (15.4)
Hospital supplied scrubs	1.15	1.04 – 1.27	0.007	124/1056 (11.7)
Own scrubs	1.04	0.88 – 1.23	0.62	26/256 (10.2)
Own clothes to work	0.95	0.80 – 1.13	0.54	165/1684 (9.8)
Use changing room at work	1.04	0.92 – 1.18	0.52	139/1323 (10.5)

For peer review only

Dedicated footwear for work	1.14	0.97 – 1.33	0.11	151/1390 (10.9)
Wear own clothes when going home	1.00	0.87 – 1.16	0.95	90/937 (9.6)
Re-usable water bottle	1.15	0.98 – 1.29	0.56	178/1760 (10.1)
Adherence to handwashing technique	1.15	0.88 – 1.51	0.31	-
Handwashing frequency	1.00	0.76 – 1.32	0.99	-
Work from home March	0.60	0.39 – 0.91	0.016	27/410 (6.6)
- For shielding?	0.58	0.36	0.94	4/62 (6.5)
Work from home June	0.58	0.36 – 0.94	0.026	2-/314 (6.4)
- For shielding?	1.81	0.63 – 5.22	0.27	5/50 (10)

For peer review only

D. Behavioural factors and association with risk of SARS-CoV-2 infection in HCWs.

Variable	OR	95 % CI	p-value	n (positive) / N (responses) (%)
Smoker	0.37	0.18 – 0.76	0.007	8/196 (4.1)
Quantity smoked	0.54	0.20 – 1.48	0.23	-
Alcohol	0.74	0.55 – 0.98	0.038	78/864 (4.1)
Frequency of alcohol	0.91	0.66 – 1.24	0.54	-
Shopping frequency March	0.91	0.75 – 1.10	0.32	-
Shopping frequency June	0.90	0.74 – 1.08	0.26	-
Contact with people March	1.04	0.86 – 1.25	0.72	-
Contact with people June	1.06	0.90 – 0.25	0.50	-
Food deliveries march				
- Less than once/week	1	-	-	141/1406 (10.0)
- Once a week	0.96	0.70 – 1.31	0.81	64/661 (9.7)
- 2-3 times / week	0.68	0.35 – 1.31	0.25	10/143 (7.0)
- Daily	5.38	1.27 – 22.8	0.022	3/8 (37.5)
Food deliveries June				

For peer review only

- Less than once/week	1	-	-	136/1376 (9.9)
- Once a week	0.96	0.70 – 1.30	0.78	66/695 (9.5)
- 2-3 times / week	1.01	0.58 – 1.78	0.96	15/150 (10)
- Daily	6.10	1.01 – 36.7	0.049	2/5 (40)
Exercise outdoors March				
- Less than once/week	1	-	-	53/428 (12.4)
- Once a week	1.07	0.69 – 1.66	0.77	40/305 (13)
- 2-3 times / week	0.73	0.50 – 1.06	0.10	72/770 (9.4)
- Daily	0.58	0.39 – 0.86	0.007	54/718 (7.5)
Exercise outdoors June				
- Less than once/week	1	-	-	51/405 (12.6)
- Once a week	0.89	0.57 – 1.41	0.63	35/307 (11.4)
- 2-3 times / week	0.76	0.52 – 1.10	0.14	79/804 (9.8)
- Daily	0.56	0.37 – 0.84	0.005	53/709
Public transport March	0.81	0.64 – 1.04	0.10	-
Public transport June	0.80	0.63 – 1.01	0.06	-
Facemask March	0.99	0.90 – 1.10	0.86	-

For peer review only

Facemask June	1.0	0.88 – 1.13	1.0	-
No social distancing March	1.74	1.31 – 2.30	<0.0001	127/1021 (12.4)
No social distancing June	1.31	1.0 – 1.73	0.06	105/933 (11.3)
Work duties altered for risk	1.12	0.75 – 1.69	0.58	40/278 (10.8)

For peer review only

E. Health factors and association with risk of SARS-CoV-2 infection in HCWs.

**Variable**	**OR**	**95 % CI**	**p-value**	**n (positive) / N (responses) (%)**
Told overweight	0.88	0.63 – 1.23	0.46	49/543 (9.0)
Told obese	0.93	0.58 – 1.49	0.77	21/225 (9.3)
Exercise frequency				
- 2-3 times/week	1	-	-	101/919 (11.0)
- Daily	0.67	0.46 – 0.97	0.033	43/566 (7.6)
- Once a week	0.91	0.61 – 1.35	0.64	37/367 (10.1)
- < once a week	0.91	0.61 – 1.34	0.63	39/387 (10.1)
Heart disease	1.17	0.41 – 3.34	0.77	4/35 (11.4)
Lung disease	0.74	0.36 – 1.55	0.43	8/106 (7.6)
Kidney disease	-	-	-	0/10 (0)
High BP	0.75	0.46 – 1.23	0.26	19/244 (7.8)
- BP medicated	1.03	0.35 – 1.97	0.96	14/175 (8.0)
- Medication #	0.89	0.55 – 1.46	0.65	-
T1 DM	2.01	0.43 – 9.38	0.37	2/11 (18.2)
T2 DM	1.17	0.46 – 3.02	0.74	5/44 (11.4)

For peer review only

- Insulin	-	-	-	0/3 (0)
- Medication #	0.75	0.29 – 1.91	0.54	-
Immunosuppression	1.3	0.51 – 3.36	0.59	5/40
Blood disorder	1.84	0.40 – 8.4	0.43	2/12 (16.7)
Inherited	1.65	0.36 – 7.51	0.52	2/13 (15.4)
Organ transplant	-	-	-	0/0 (0)
Cancer treatment	-	-	-	0/9 (0)
Currently taking:				
- Hydroxychloroquine	0.48	0.06 – 3.6	0.48	1/20 (5.0)
- Aspirin	1.0	0.58 – 1.74	0.99	15/152 (9.9)
- ACE inhibitors	1.52	0.74 – 3.12	0.25	9/64 (14.1)
- ARBs	0.74	0.23 – 2.42	0.62	3/40 (7.5)
- Tacrolimus	2.30	0.26 – 20.66	0.46	1/5 (20)
- Mycophenolate	2.30	0.26 – 20.66	0.46	1/5 (20)
- Prednisolone	1.59	0.61 – 4.16	0.34	5/34 (14.7)
- Tocilizumab	1.84	0.21 – 15.8	0.58	1/6 (16.7)
- Azathioprine	2.31	0.49 – 10.92	0.29	2/10 (20)

For peer review only

- Methotrexate	1.15	0.26 – 5.03	0.86	2/18 (11.1)
- Cyclosporine	2.30	0.26 – 20.66	0.46	1/5 (20)
- Leflunomide	1.53	0.18 – 12.77	0.69	1/7 (14.3)
Ever had:				
- Rituximab	0.83	0.11 – 6.48	0.86	1/12 (8.3)
- Abatacept	2.31	0.26 – 20.66	0.46	1/5 (20)
- Adalimumab	1.15	0.14 – 9.21	0.90	1/9 (11.1)
- Etanercept	0.83	0.11 – 6.48	0.86	1/12 (8.3)
- Infliximab	2.31	0.49 – 10.92	0.29	2/10 (20)
- Basiliximab	2.30	0.26 – 20.66	0.46	1/5 (20)
- Cyclophosphamide	0.76	0.10 – 5.90	0.80	1/13 (7.7)
Chemotherapy for cancer	0.65	0.20 – 2.11	0.47	3/45 (6.7)

For peer review only

Reporting checklist for cross sectional study.

Based on the STROBE cross sectional guidelines.

Instructions to authors

Complete this checklist by entering the page numbers from your manuscript where readers will find each of the items listed below.

Your article may not currently address all the items on the checklist. Please modify your text to include the missing information. If you are certain that an item does not apply, please write "n/a" and provide a short explanation.

Upload your completed checklist as an extra file when you submit to a journal.

In your methods section, say that you used the STROBE cross sectionalreporting guidelines, and cite them as:

von Elm E, Altman DG, Egger M, Pocock SJ, Gotzsche PC, Vandenbroucke JP. The Strengthening the Reporting of Observational Studies in Epidemiology (STROBE) Statement: guidelines for reporting observational studies.

For peer review only

		Reporting Item	Page Number
**Title and abstract**			
Title	#1a	Indicate the study's design with a commonly used term in the title or the abstract	1

Abstract	#1b	Provide in the abstract an informative and balanced summary of what was done and what was found	3-4
**Introduction**			
Background / rationale	#2	Explain the scientific background and rationale for the investigation being reported	5
Objectives	#3	State specific objectives, including any prespecified hypotheses	5
**Methods**			
Study design	#4	Present key elements of study design early in the paper	6
Setting	#5	Describe the setting, locations, and relevant dates, including periods of recruitment, exposure, follow-up, and data collection	6
Eligibility criteria	#6a	Give the eligibility criteria, and the sources and methods of selection of participants.	7
	#7	Clearly define all outcomes, exposures, predictors, potential confounders, and effect modifiers. Give diagnostic criteria, if applicable	7
Data sources / measurement	#8	For each variable of interest give sources of data and details of methods of assessment (measurement). Describe comparability of assessment methods if there is more than one group. Give information separately for for exposed and unexposed groups if applicable.	7

For peer review only

Bias	#9	Describe any efforts to address potential sources of bias	7
Study size	#10	Explain how the study size was arrived at	8
Quantitative variables	#11	Explain how quantitative variables were handled in the analyses. If applicable, describe which groupings were chosen, and why	7-8
Statistical methods	#12a	Describe all statistical methods, including those used to control for confounding	7-8
Statistical methods	#12b	Describe any methods used to examine subgroups and interactions	7-8
Statistical methods	#12c	Explain how missing data were addressed	-
Statistical methods	#12d	If applicable, describe analytical methods taking account of sampling strategy	-
Statistical methods	#12e	Describe any sensitivity analyses	-
**Results**			
Participants	#13a	Report numbers of individuals at each stage of study—eg numbers potentially eligible, examined for eligibility, confirmed eligible, included in the study, completing follow-up, and analysed. Give information separately for for exposed and unexposed groups if applicable.	8
Participants	#13b	Give reasons for non-participation at each stage	-

For peer review only

Participants	#13c	Consider use of a flow diagram	
Descriptive data	#14a	Give characteristics of study participants (eg demographic, clinical, social) and information on exposures and potential confounders. Give information separately for exposed and unexposed groups if applicable.	Page 8; Table 1
Descriptive data	#14b	Indicate number of participants with missing data for each variable of interest	Table 2
Outcome data	#15	Report numbers of outcome events or summary measures. Give information separately for exposed and unexposed groups if applicable.	Table 2
Main results	#16a	Give unadjusted estimates and, if applicable, confounder-adjusted estimates and their precision (eg, 95% confidence interval). Make clear which confounders were adjusted for and why they were included	8-10
Main results	#16b	Report category boundaries when continuous variables were categorized	-
Main results	#16c	If relevant, consider translating estimates of relative risk into absolute risk for a meaningful time period	-
Other analyses	#17	Report other analyses done—e.g., analyses of subgroups and interactions, and sensitivity analyses	10

Discussion

Key results	#18	Summarise key results with reference to study objectives	11

For peer review only

Limitations	#19	Discuss limitations of the study, taking into account sources of potential bias or imprecision. Discuss both direction and magnitude of any potential bias.	13
Interpretation	#20	Give a cautious overall interpretation considering objectives, limitations, multiplicity of analyses, results from similar studies, and other relevant evidence.	13
Generalisability	#21	Discuss the generalisability (external validity) of the study results	13
**Other Information**			
Funding	#22	Give the source of funding and the role of the funders for the present study and, if applicable, for the original study on which the present article is based	14

None The STROBE checklist is distributed under the terms of the Creative Commons Attribution License CC-BY. This checklist can be completed online using https://www.goodreports.org/, a tool made by the EQUATOR Network in collaboration with Penelope.ai

For peer review only

BMJ Open

A retrospective observational study of demographic, behavioural and occupational risk factors associated with SARS-COV-2 infection in UK healthcare workers

Journal:	*BMJ Open*
Manuscript ID	bmjopen-2022-063159.R1
Article Type:	Original research
Date Submitted by the Author:	22-Sep-2022
Complete List of Authors:	Cooper, Daniel; University of Cambridge School of Clinical Medicine, Department of Medicine ; Cambridge University Hospitals NHS Foundation Trust<br>Lear , Sara; Cambridge University Hospitals NHS Foundation Trust<br>Sithole, Nyarie; Cambridge University Hospitals NHS Foundation Trust<br>Shaw, Ashley; Cambridge University Hospitals NHS Foundation Trust, Medical Director's Office<br>Stark , Hannah; NIHR Cambridge Biomedical Research Centre, NIHR Bioresource<br>Ferris, Mark; Cambridge University Hospitals NHS Foundation Trust, Occupational Health<br>COVID-19 collaboration, CITIID-NIHR BioResource ; NIHR Cambridge Biomedical Research Centre, NIHR Bioresource<br>Bradley, John; University of Cambridge, Department of Medicine; Cambridge University Hospitals NHS Foundation Trust<br>Maxwell, Patrick ; University of Cambridge School of Clinical Medicine, Department of Medicine ; Cambridge University Hospitals NHS Foundation Trust<br>Goodfellow, Ian; University of Cambridge, Department of Pathology, Division of Virology<br>Weekes, Michael; University of Cambridge; Cambridge University Hospitals NHS Foundation Trust<br>Seaman, Shaun; MRC Biostatistics Unit,<br>Baker, Stephen; University of Cambridge School of Clinical Medicine, Cambridge Institute of Therapeutic Immunology and Infectious Disease
**Primary Subject Heading**:	Infectious diseases
Secondary Subject Heading:	Epidemiology, Public health
Keywords:	COVID-19, Public health < INFECTIOUS DISEASES, Infection control < INFECTIOUS DISEASES, Epidemiology < INFECTIOUS DISEASES

SCHOLARONE™
Manuscripts

I, the Submitting Author has the right to grant and does grant on behalf of all authors of the Work (as defined in the below author licence), an exclusive licence and/or a non-exclusive licence for contributions from authors who are: i) UK Crown employees; ii) where BMJ has agreed a CC-BY licence shall apply, and/or iii) in accordance with the terms applicable for US Federal Government officers or employees acting as part of their official duties; on a worldwide, perpetual, irrevocable, royalty-free basis to BMJ Publishing Group Ltd ("BMJ") its licensees and where the relevant Journal is co-owned by BMJ to the co-owners of the Journal, to publish the Work in this journal and any other BMJ products and to exploit all rights, as set out in our licence.

The Submitting Author accepts and understands that any supply made under these terms is made by BMJ to the Submitting Author unless you are acting as an employee on behalf of your employer or a postgraduate student of an affiliated institution which is paying any applicable article publishing charge ("APC") for Open Access articles. Where the Submitting Author wishes to make the Work available on an Open Access basis (and intends to pay the relevant APC), the terms of reuse of such Open Access shall be governed by a Creative Commons licence – details of these licences and which Creative Commons licence will apply to this Work are set out in our licence referred to above.

Other than as permitted in any relevant BMJ Author's Self Archiving Policies, I confirm this Work has not been accepted for publication elsewhere, is not being considered for publication elsewhere and does not duplicate material already published. I confirm all authors consent to publication of this Work and authorise the granting of this licence.

For peer review only

A retrospective observational study of demographic, behavioural and occupational risk factors associated with SARS-COV-2 infection in UK healthcare workers

Daniel J. Cooper[1, 2, 3*], Sara Lear[2], Nyarie Sithole[2], Ashley Shaw[2], Hannah Stark[4], Mark Ferris[2], CITIID-NIHR BioResource COVID-19 collaboration consortium, John R. Bradley[1, 2], Patrick H. Maxwell[1, 2], Ian Goodfellow[5], Michael P. Weekes[1, 2, 6], Shaun Seaman[7], Stephen Baker[8]

1. Department of Medicine, Cambridge University School of Clinical Medicine, Cambridge, UK.
2. Cambridge University Hospitals NHS Foundation Trust, Cambridge, UK.
3. Global and Tropical Health Division, Menzies School of Heath Research and Charles Darwin University, Darwin, Northern Territory, Australia
4. NIHR Bioresource, NIHR Cambridge Biomedical Research Centre, Cambridge Biomedical Campus, Cambridge, UK
5. Department of Pathology, Division of Virology, University of Cambridge, Cambridge Biomedical Campus, Cambridge, UK
6. Cambridge Institute for Medical Research, Cambridge, UK
7. MRC Biostatistics Unit, University of Cambridge, Cambridge, UK
8. Cambridge Institute of Therapeutic Immunology and Infectious Disease, University of Cambridge School of Clinical Medicine, Cambridge Biomedical Campus, Cambridge, UK.

* Corresponding author

Keywords: COVID-19; Healthcare Workers; Epidemiology; Public Health

Word count: 3108

For peer review only

For peer review only - http://bmjopen.bmj.com/site/about/guidelines.xhtml

Corresponding author:

Dr Daniel Cooper

Department of Medicine

University of Cambridge

E: Dc801@cam.ac.uk

T: 07843662562

For peer review only

ABSTRACT

Objective: Healthcare workers (HCWs) are at higher risk of SARS-COV-2 infection than the general population. This group are pivotal to healthcare system resilience during the COVID-19, and future, pandemics. We investigated demographic, social, behavioural and occupational risk factors for SARS-CoV-2 infection amongst HCWs.

Design/Setting/Participants: HCWs enrolled in a large-scale sero-epidemiological study at a UK university teaching hospital were sent questionnaires spanning a 5-month period from March–July 2020. In a retrospective observational cohort study, univariate logistic regression was used to assess factors associated with SARS-COV- 2 infection. A Least Absolute Shrinkage Selection Operator (LASSO) regression model was used to identify variables to include in a multivariate logistic regression model.

Results: Amongst 2,258 HCWs, highest Odds Ratios associated with SARS-CoV-2 antibody seropositivity on multivariate analysis were having a household member previously testing positive for SARS-CoV-2 antibodies (OR 6.94 [95% CI 4.15 – 11.6]; p<0.0001) and being of Black ethnicity (6.21 [2.69 – 14.3]; p<0.0001). Occupational factors associated with a higher risk of seropositivity included working as a physiotherapist (OR 2.78 [1.21 – 6.36]; p=0.015) and working predominantly in acute medicine (OR 2.72 [1.57 – 4.69]; p=<0.0001) or medical subspecialities (not including infectious diseases) (OR 2.33 [1.4 – 3.88]; p=0.001). Reporting that adequate PPE was “rarely” available had an OR of 2.83 (1.29 – 6.25; p=0.01). Reporting attending a handover where social distancing was not possible had an OR of 1.39 (1.02 – 1.9; p=0.038).

Conclusions: The emergence of SARS-CoV-2 variants and potential vaccine-escape continue to threaten stability of healthcare systems worldwide and sustained vigilance against HCW infection

For peer review only

remains a priority. Enhanced risk assessments should be considered for HCWs of Black ethnicity, physiotherapists and those working in acute medicine or medical subspecialties. Workplace risk reduction measures include ongoing access to high-quality PPE and effective social distancing measures.

Strengths and limitations of this study:

- A strength of this study was the use of a large, well-defined cohort of UK healthcare workers
- The identification of actionable risk factors for mitigation of HCW infection
- Representative and transferable conclusions for acute hospital trusts
- Limitations include some potential retrospective recall bias of subjective questionnaire responses

For peer review only - http://bmjopen.bmj.com/site/about/guidelines.xhtml

BACKGROUND

The COVID-19 pandemic continues to overwhelm healthcare services globally with substantial morbidity and mortality [1]. The COVID-19 vaccination programme has been a major success in the UK, having a major impact on reducing hospitalisations and death [2,3]. However, the recent upsurge of cases associated with the delta variant [4] followed by emergence and dominance of the Omicron variant [4] illustrates how management of the pandemic requires sustained vigilance from the general public, policy makers, and healthcare workers (HCWs). Notably, the delta [5] and omicron [6] variants have increased transmissibility, and a reduced efficacy of vaccination for prevention of infection [7-10]. Therefore, the emergence of additional variants with the potential for vaccine escape are a genuine concern for how we control SARS-CoV-2 in the long term.

HCWs are at a disproportionately high risk of infection from SARS-CoV-2 [11] but remain key to the resilience of the health service during this, and all future pandemics. Infections of HCWs with SARS-CoV-2 and the isolation of contacts has resulted in significant staff shortages and increased strain on UK hospitals. Staff absence during September 2021 (most recent available figures) was 5.4% across the NHS; higher than August 2021 (5.1%) and higher than September 2020 (4.2%) [12]. This high level of absence is despite the high rates of vaccination in HCWs, where up to 92.3% of staff in NHS trusts have received at least 2 doses of vaccine as of 28th February 2022 [13]. Measures to reduce the risk of SARS-CoV-2 exposure to HCWs alongside widespread vaccination are vital to create resilience within the healthcare system. We have previously identified several occupational factors associated with increased risk of SARS-CoV-2 seropositivity in HCWs, which included job role, work location and ethnicity [14]. We conducted a retrospective observational cohort study in HCWs working in a major tertiary referral centre in the East of England with the objective of further elucidating the social, demographic, occupational and physical factors that may contribute to a higher risk of SARS-CoV-2 infection in HCWs.

For peer review only - http://bmjopen.bmj.com/site/about/guidelines.xhtml

Methods

Population and setting

Cambridge University Hospitals NHS Foundation Trust (CUH) is a tertiary referral centre and teaching hospital with 1,000 beds and 11,545 staff serving a population of 580,000 people in the East of England. The facility was equipped with 43 ICU beds prior to the pandemic, rising to 103 ICU beds at the peak of the pandemic, and an Emergency Department that receives ~14,000 attendees a month. During the study period (between March and June 2020), CUH treated 525 patients with PCR-confirmed COVID-19. The peak of COVID-19 admissions occurred in late March and early April 2020, with comparatively few COVID-19 admissions from June 2020 to November 2020. The definition of COVID-19 working for the purpose of risk stratification included clinical areas caring for patients with PCR-confirmed SARS-CoV-2 infection) and those with patients for whom there is a high clinical suspicion of COVID-19, awaiting the results of SARS-CoV-2 PCR tests.

According to the 2011 England and Wales census [15], 85·3% of the population of the East of England are White British, 5·5% are White Other, 4·8% are Asian, 2% are Black, and 1·9% are of Mixed ethnicity. The proportion of Black, Asian, and Minority Ethnic (BAME) staff employed at CUH at the time of the study was largely representative of the overall NHS workforce [16] (21·2% vs 20·7%, respectively).

A staff screening programme for SARS-CoV-2 serological testing was available from 10th June 2020 to 7th August 2020 and has been described previously [14] (detailed in **Figure 1**). In brief, all staff members were invited by email to participate in the serological screening programme and asked to self-refer for a clinic appointment. Written informed consent was obtained from all participants enrolled into this study. As part of this process all participants were invited to join the NIHR BioResource – COVID-19 Research Cohort (IRAS 220277). Basic demographic and occupational information were recorded and a serum sample was taken and assayed for total SARS-CoV-2 antibodies (detailed below).

For peer review only - http://bmjopen.bmj.com/site/about/guidelines.xhtml

As no prior data were available to assess between-group differences on the metrics assessed in this study, a formal sample size calculation was not feasible.

Questionnaire

A questionnaire covering demographic, occupational and behavioural factors potentially associated with risk of infection was designed with input and pre-testing from infectious disease physicians, occupational physicians, virologists, microbiologists, and epidemiologists. Formal reliability testing was not performed. Participants previously enrolled in a longitudinal HCW serological study (as described above) were invited by email to complete an online form containing the questionnaire in English, with the option to request the questionnaire in another language. A copy of the questions included in this questionnaire is included as **Supplementary appendix 1**. Questionnaire invites were sent between October and November 2021 and questions within them related to participants' recalled behaviour during two periods: March – May 2020 and June – July 2020. Questions relating to behavioural and demographic factors were separated by time periods covering March – May and June – July to account for differences in behaviour and exposures outside of occupational environments due to the instigation (March 2020) and easing (June 2020) of the first UK national "lockdown" measures.

Laboratory assays

Serological testing for antibodies directed against SARS-CoV-2 was performed using the Centaur XP SARS-Cov-2 Total Antibody assay (Siemens Healthcare Limited, Surrey, UK). This method is a fully automated high throughput enzyme linked chemiluminescent bridging immunoassay which targets the S1RBD antigen of SARS-CoV-2 and can detect all Ig subclasses (IgG, IgM, and IgA). The method was independently validated by Public Health England and has a reported sensitivity and specificity of 98.1% (95% CI 96.6 – 99.1) and 99.9% (95% CI 99.4 – 100)[17] respectively. Samples were processed in the Biochemistry laboratory at CUH following the SOP as stated by the manufacturer in their Instruction for Use (IFU) after a local verification using guidance from The Royal College of Pathologists [18].

For peer review only - http://bmjopen.bmj.com/site/about/guidelines.xhtml

Statistical analysis

Univariate logistic regression was used to assess each variable in the questionnaire for association with positive SARS-CoV-2 antibodies. Variables with a p-value of <0.05 on univariate analysis were included in a Least Absolute Shrinkage and Selection Operator (LASSO) regression analysis with post-estimation extended Bayesian information criterion commands for variable selection to include in a multivariate logistic regression model. The LASSO method of variable selection was used, in preference to the older stepwise selection method, because it has been shown to lead to higher prediction accuracy and variable selection that is less sensitive to small changes in the data [19,20]. Variables selected by LASSO analysis were included in a final multivariate logistic regression model. Data were analysed using Stata v14.2 (StataCorp, College Station, Texas).

Ethical Approval

Ethical approval for this study was granted by the East of England – Cambridge Central Research Ethics Committee (IRAS ID: 220277).

Patient and public involvement

Staff at CUH contributed to study and questionnaire design.

RESULTS

Baseline characteristics

A total of 2,258 of 5,698 (40%) invited HCWs responded to the invitation to complete an online questionnaire. Of the participants that responded to join the study, 19.65% (400/2,044 responses) were male, the median age was 42 years (IQR 32 – 53 years), and 27.7% (618/2,044) reported working in a designated COVID-19 "red" area during this first wave of the pandemic. Notably, 9.8% (n=222/2,044) of the cohort tested seropositive for SARS-CoV-2 antibodies. The demographics of the

For peer review only - http://bmjopen.bmj.com/site/about/guidelines.xhtml

study group are shown in **Table 1**. Full details of variables and questionnaire responses are available in the Supplementary Tables A – E.

Univariate analysis

Reponses demonstrated to have a significant association (*p*<0.05) with seropositivity in a univariate analysis are described in **Table 2 (**The odds ratios and *p*-values for responses to all questions are listed in **Supplementary Tables A – E)**. Noteworthy variables significantly associated with seropositivity for SARS-CoV-2 antibodies included having a household member that had tested positive for SARS-CoV-2 by PCR prior to staff serology testing (OR 3.48 [95% CI 2.09 – 5.78]; *p*<0.001), or had tested positive by a SARS-CoV-2 antibody test (OR 11.3 [95% CI 7.08 – 18.01]; *p*<0.001), or had had a household member who had been symptomatic (OR 3.71 [95% CI 2.8 – 4.96]; *p*<0.001). Other demographic factors that were positively associated with seropositivity include identifying as being Asian or Asian British – other (OR 2.14 [1.27 – 3.60; *p*=0.004]), mixed ethnicity (OR 4.68 [1.20 – 18.29; *p*=0.027]), or Black or Black British – African ethnicity (5.74 [2.61 – 12.60; p<0.001). Notably, reporting being born in the UK was associated with a protective effect (OR 0.59 [95% CI 2.8 – 4.96]; *p*<0.001).

Renting a room in a shared house (OR 1.84 [1.22 – 2.74]; *p*=0.003) and living with another healthcare worker (OR 1.49 [95% CI 1.10 – 2.02]; *p*=0.009) were further demographic factors associated with a significantly higher risk of infection on univariate analysis.

Other than job role, specialty and direct COVID patient care, a number of other occupational factors were associated with higher odds of infection, including working night shifts (OR 1.68 [1.26 – 2.25]; *p*<0.001), using the doctors' mess (OR 1.77 [1.17 – 2.69]; *p*=0.007), spending rest or meal time with colleagues "most of the time" (OR 1.99 [95% CI 1.19 – 3.33]; *p*=0.009), and using hospital supplied scrubs (OR 1.15 [95% CI 1.04 – 1.27]; *p*=0.007). Those reporting having received formal PPE training had a 40% higher risk of infection (OR 1.4 [95% CI 1.05 – 1.85]; *p*=0.02) than those who did not. A higher proportion of those that worked in COVID red areas reported receiving formal PPE

For peer review only - http://bmjopen.bmj.com/site/about/guidelines.xhtml

training (486/613, 79%) than those not working in COVID red areas (646/1594, 41%; $p<0.0001$), and formal PPE training no longer remained significant when controlling for "red area" working (OR 1.2 [0.88 – 1.63]; $p=0.20$).

Those reporting having adequate PPE available "some of the time" (OR 1.93 [95% CI 1.22 – 3.05]; $p=0.005$) or "rarely" (OR 3.60 [95% CI 1.71 – 7.57]; $p=0.001$) were associated with a higher odds of infection compared to those who reported adequate PPE being available "all of the time". A higher proportion of those reporting PPE being available "some of the time" (78/194, 40%) worked in COVID red areas compared to those reporting PPE being available "all of the time" (540/2041, 27%; p<0.0001). Attending shift handover (a staff meeting prior to shift change) where social distancing was not possible was associated with a higher risk of infection (OR 1.74 [95% CI 1.31 – 2.30]; $p<0.001$). Working predominantly from home between March – June 2020 was associated with a protective effect (OR 0.60 [95% CI 0.39 – 0.91]; $p=0.016$), as was working from home between June – July 2019 (OR 0.58 [95% CI 0.36 – 0.94]; $p=0.026$)

Reporting being a smoker was associated with a lower risk of infection (OR 0.37 [95% CI 0.18 – 0.76]; $p=0.007$) amongst behavioural risk factors. Reporting drinking alcohol was associated with a lower risk of infection (OR 0.74 [95% CI 0.55 – 0.98]; $p=0.38$), however frequency of drinking alcohol had no effect on risk of infection. Having food or grocery deliveries to home "daily" was associated with a higher risk of infection between March – May 2020 (OR 5.38 [95% CI 1.27 – 22.8]; $p=0.022$) and June to July 2020 (OR 6.1 [95% CI 1.01 to 36.7]; $p=0.049$) compared to those who reported "never" having food or groceries delivered. Exercising outdoors "daily" was associated with a lower risk of infection between March – May 2020 (OR 0.58 [95% CI 0.39 – 0.86]; $p=0.007$) and between June – July 2020 (OR 0.56 [95% CI 0.37 – 0.84[; $p=0.005$) compared to those who reported exercising outdoors less than once per week.

For peer review only - http://bmjopen.bmj.com/site/about/guidelines.xhtml

LASSO model fitting

The variables selected by the LASSO model are shown in **Table 3,** and included having a household member that had tested positive for SARS-CoV-2 antibodies or had had a positive SARS-CoV-2 PCR test, a household member previously displaying symptoms synonymous with COVID-19, Black ethnicity, working as a Physiotherapist, reporting working in acute medicine or medical subspecialities, reporting that adequate PPE was "rarely" available, working in a designated "Red" area, and attending handovers where adequate social distancing was not possible.

Multivariate analysis

We used a multivariate logistic regression model to include all variables selected by LASSO modelling. In this model, working in a designated COVID-19 area and having a household member with a previous positive SARS-CoV-2 PCR swab were not significantly associated with the participant having a positive antibody test result (p>0.05), and were dropped from the final model. A total of eight variables were included in the final multivariate logistic regression model (**Table 3**; **Figure** 2).

In this resulting model the highest reported adjusted Odds Ratios associated with participants testing seropositive for SARS-CoV-2 antibodies were having a household member that had previous tested positive for SARS-CoV-2 antibodies (OR 6.94 [95% CI 4.15 – 11.6]; p<0.0001) and being of Black ethnicity (6.21 [95% CI 2.69 – 14.3]; p<0.0001). Occupational factors associated with a higher risk of seropositivity were working as a physiotherapist (aOR 2.78 [95% CI 1.21 – 6.36]; p=0.015) and reporting that they predominantly worked in acute medicine (aOR 2.72 [95% CI 1.57 – 4.69]; p<0.0001) or medical subspecialities (not including infectious diseases) (aOR 2.33 [95% CI 1.4 – 3.88]; p=0.001). Reporting that adequate PPE was "rarely" available was associated with an aOR of 2.83 (95% CI 1.29 – 6.25; p=0.01) and reporting attending a handover where social distancing was not possible was associated with an aOR of 1.39 (95% CI 1.02 – 1.9; p=0.038).

For peer review only - http://bmjopen.bmj.com/site/about/guidelines.xhtml

DISCUSSION

In this systematic evaluation of demographic, occupational and behavioural risk factors associated with COVID-19 seropositivity amongst HCWs, we have identified several targetable risk factors for HCW infection from SARS-CoV-2. These may also serve as a framework for targeting HCW risk during future respiratory pathogen pandemics. The ability of healthcare systems to cope with surges of infections requiring hospitalisation has been challenged in a number of countries including the UK [21], India [22], USA [23] and Brazil [24] and resulted in excess deaths [23,25]. The resilience of a healthcare system relies heavily on staff remaining well and able to work. Healthcare workers have been disproportionately affected by infection rates [26,27] during this pandemic.

Both a positive SARS-CoV-2 antibody in a household member and prior symptoms in a household member were significantly associated with seropositivity in a multivariate model. The finding that a positive PCR test in a household member was not associated with seropositivity on multivariate analysis may reflect a proportional relationship between viral load and transmissibility in asymptomatic infections. A study of Ct threshold values (as a proxy for viral load) in uncomplicated community SARS-CoV-2 demonstrated that self-reported symptoms were an independent predictor of lower Ct value (i.e. higher viral load), and that Ct values were significantly higher in those who remained antibody negative [28]. Taken together, these results suggest that a household member with positive symptoms (and either untested or false negative test) or a high enough viral load to develop antibodies contribute more to risk of infection in household members than a positive PCR test alone.

The finding that Black ethnicity remained highly significantly associated with seropositivity after controlling for many plausible explanations is concerning. The effect of increased risk of infection in certain ethnicities has been reported elsewhere; the reasons for this are complex and remain poorly understood but may include increased risk of household transmission [29]. South Asian and Black ethnicity have been found to be associated with a higher risk of hospitalisation, ICU admission and death relative to white ethnicity [30]. An increased risk of infection in non-white ethnicity has been reported across multiple other studies in other countries and healthcare settings, including Black and

For peer review only - http://bmjopen.bmj.com/site/about/guidelines.xhtml

Asian staff in UK hospitals [31], Black staff in US health care systems [32,33], non-white workers in Brazil [34], and Black or Hispanic ethnicity in Canada [35]. Observational studies in countries not assessing ethnicity in HCW risk-factor analyses have reported risks that have been suggested as potentially contributing to health disparities in non-white ethnicities including income level, educational background, and use of mass-transit systems [34,36].

The subjective feeling that adequate PPE was rarely available remained highly statistically significant in the final multivariate model. Whilst interesting, this finding requires a careful consideration of context and the subjective nature of the question. The availability and standard of PPE at CUH has been reported as exceeding that recommended by Public Health England for HCWs during the period of the study [37]. Furthermore, we have demonstrated elsewhere that the use of this enhanced PPE was effective at reducing the risk of infection amongst HCWs [37]. CUH reported the second-lowest number of hospital acquired COVID-19 cases in the East of England [38] out of 14 hospital trusts (suggesting high standards of infection control), with clinical outcomes for COVID-19 patients exceeding the national standard [39]. Despite these factors, 11% of staff reported the perception that adequate PPE was available “some of the time” or “rarely”. Similar data are not available for comparison at other NHS sites. Staff at a higher risk of occupational exposure to infectious patients are likely to have experienced higher rates of anxiety related to PPE and therefore recall that anxiety, especially within the wider context of the media reporting of the national and global effects of the COVID-19 pandemic during that time. This is demonstrated in the higher proportion of those reporting insufficient PPE being available “some of the time” or “rarely” working in COVID-19 red areas compared to those reporting adequate PPE being available “all of the time”. Nevertheless, the fact that this variable remained highly significant after LASSO variable selection and inclusion in the multivariate model highlights the need for availability of effective PPE for all HCWs at occupational risk of infection. Effective PPE is key for reducing infection, but also staff mental well-being and reducing potential burnout [40].

For peer review only - http://bmjopen.bmj.com/site/about/guidelines.xhtml

The impact of social distancing on the risk of COVID-19 infection is now well documented [41,42]. Our analysis suggests that the practice of social distancing and mask wearing during shift-change handovers and other meeting times should continue to be encouraged as a modifiable behaviour that has the potential to decrease the risk of SARS-CoV-2 infection in HCWs.

Physiotherapy played a key role in both ICU and acute medical wards with therapeutic positioning, early mobilisation and breathing exercises [43]. In addition, the risk of hospitalisation with COVID-19 increases with age, and elderly populations constituted a large proportion of non-ICU hospital admissions [44]. Physiotherapists constitute an integral part of a face-to-face multidisciplinary team during acute hospital admissions for elderly people [45], and would therefore have had significant exposure to SARS-CoV-2 infected patients. The increased risk of infection amongst physiotherapists during these activities requires further investigation and should be considered when assessing clinical practise risk and PPE standards.

These analyses have limitations. By their nature, questions about behavioural factors contain subjective answers, and must be interpreted with caution, including the subjective experience of availability of PPE. In addition, the questionnaire was sent to participants 3 -7 months following the period encompassed by the questions, which could add imprecision. This delay leaves responses open to recall bias, however most important factors assessed here (ethnicity, job role, prior household PCR and antibody results) are objective and are unlikely to have changed in the intervening period. Participants were aware of their serostatus at the time of completing the questionnaire, which may also have influenced responses to subjective questions, particularly around the availability of PPE. We have previously shown that porters and domestic staff are at a higher risk of infection [46], however their experience was not captured in this study due to low numbers of respondents (n=7 and n=0 respectively). These analyses cover the time period where the original wild type Wuhan strain was the predominant circulating variant in the UK. Data on established and emerging variants, including the delta variant [4] and the now predominant Omicron variant [6], suggest they may be more infectious and thus levels of risk and risk factors may not be identical. We think that the risk factors discussed

For peer review only - http://bmjopen.bmj.com/site/about/guidelines.xhtml

within this paper are unlikely to be greatly affected by a change in the risk of infection in new variants and remain broadly generalisable as risk factors for HCW infection, although the widespread introduction of both population and HCW vaccination since this study is likely to have had a significant impact on these risk factors [47].

Our work identified a number of targetable risk factors for mitigation of the risk of HCW infection during the ongoing COVID-19 pandemic. Maintaining vigilance and providing adequate social distancing space for shift-change handover is likely to reduce the risk of HCW infection. The subjective experience of staff towards PPE should be considered when providing adequate and safe PPE provision and training. In addition, there are a number of non-modifiable risk factors, which nevertheless are feasible for extra mitigation strategies for healthcare professionals working within a health service to reduce the risk of HCW infection.

Acknowledgements

We thank NIHR BioResource volunteers for their participation, and gratefully acknowledge NIHR BioResource centres, NHS Trusts and staff for their contribution. We thank the National Institute for Health Research, NHS Blood and Transplant, and Health Data Research UK as part of the Digital Innovation Hub Programme. The views expressed are those of the author(s) and not necessarily those of the NHS, the NIHR or the Department of Health and Social Care.

Author Contributions

DJC, SL and SB conceived and designed the study. DJC, SB and SS conducted the analysis. DJC, SL, SB, NS, AS, HS, MF, PHM, JB, MPW and IG contributed to questionnaire design and analysis. Operational input and analysis was provided by AS and MF. Study logistics, questionnaire distribution and data collection were performed by Cambridge NIHR BioResource and the CITIID-NIHR BioResource COVID-19 collaboration consortium, overseen by HS. All authors read the manuscript and provided edits.

For peer review only - http://bmjopen.bmj.com/site/about/guidelines.xhtml

Funding

DJC and SL received funding for this work from Addenbrooke's Charitable Trust (Grant ID 900254). The work was also funded by awards from NIHR to the NIHR BioResource (RG94028 & RG85445). This research was funded in part by the Wellcome Trust [215515/Z/19/Z Senior Fellowship to SGB, 412 207498/Z/17/Z Senior Fellowship to IGG, 108070/Z/15/Z Senior Fellowhsip to MPW]. For the purpose of open access, the author has applied a CC BY public copyright licence to any Author Accepted Manuscript version arising from this submission.

Competing Interests

The authors declare no competing interests related to this study.

Ethical Approval

Ethical approval for this study was granted by the East of England – Cambridge Central Research Ethics Committee (IRAS ID: 220277).

Data Sharing Statement

Data are available on reasonable request to the authors.

For peer review only

REFERENCES

1. WHO. WHO coronavirus disease (COVID-19) dashboard. Available at: https://covid19.who.int/. Accessed June 07.
2. Hyams C, Marlow R, Maseko Z, et al. Effectiveness of BNT162b2 and ChAdOx1 nCoV-19 COVID-19 vaccination at preventing hospitalisations in people aged at least 80 years: a test-negative, case-control study. Lancet Infect Dis **2021**.
3. Dagan N, Barda N, Kepten E, et al. BNT162b2 mRNA Covid-19 Vaccine in a Nationwide Mass Vaccination Setting. N Engl J Med **2021**; 384(15): 1412-23.
4. UK Health Security Agency. COVID-19 variants: genomically conformed case numbers. Available at: https://www.gov.uk/government/publications/covid-19-variants-genomically-confirmed-case-numbers. Accessed 08/02/2021.
5. Frampton D, Rampling T, Cross A, et al. Genomic characteristics and clinical effect of the emergent SARS-CoV-2 B.1.1.7 lineage in London, UK: a whole-genome sequencing and hospital-based cohort study. Lancet Infect Dis **2021**.
6. Song JS, Lee J, Kim M, et al. Serial Intervals and Household Transmission of SARS-CoV-2 Omicron Variant, South Korea, 2021. Emerg Infect Dis **2022**; 28(3).
7. Abu-Raddad LJ, Chemaitelly H, Butt AA, National Study Group for C-V. Effectiveness of the BNT162b2 Covid-19 Vaccine against the B.1.1.7 and B.1.351 Variants. N Engl J Med **2021**.
8. Emary KRW, Golubchik T, Aley PK, et al. Efficacy of ChAdOx1 nCoV-19 (AZD1222) vaccine against SARS-CoV-2 variant of concern 202012/01 (B.1.1.7): an exploratory analysis of a randomised controlled trial. Lancet **2021**; 397(10282): 1351-62.
9. Pajon R, Doria-Rose NA, Shen X, et al. SARS-CoV-2 Omicron Variant Neutralization after mRNA-1273 Booster Vaccination. N Engl J Med **2022**.
10. Rossler A, Riepler L, Bante D, von Laer D, Kimpel J. SARS-CoV-2 Omicron Variant Neutralization in Serum from Vaccinated and Convalescent Persons. N Engl J Med **2022**.

For peer review only - http://bmjopen.bmj.com/site/about/guidelines.xhtml

11. Nguyen LH, Drew DA, Graham MS, et al. Risk of COVID-19 among front-line health-care workers and the general community: a prospective cohort study. Lancet Public Health **2020**; 5(9): e475-e83.

12. NHS Digital. NHS Sickness Absence Rates, July 2021 to September 2021, Provisional Statistics. Published 3rd February 2022, **2022**.

13. NHS England COVID-19 vaccination statistics. https://www.england.nhs.uk/statistics/statistical-work-areas/covid-19-vaccinations/. Accessed 01/03/2022.

14. Cooper DJ, Lear S, Watson L, et al. A prospective study of risk factors associated with seroprevalence of SARS-CoV-2 antibodies in healthcare workers at a large UK teaching hospital. J Infect **2022**.

15. Office for National Statistics. 2011 UK census. Available at: https://www.ons.gov.uk/census/2011census. Accessed 23rd September 2020.

16. Gov.uk. NHS Workforce. Available at: https://www.ethnicity-facts-figures.service.gov.uk/workforce-and-business/workforce-diversity/nhs-workforce/latest. Accessed 23rd September 2020. .

17. National S-C-SAEG. Performance characteristics of five immunoassays for SARS-CoV-2: a head-to-head benchmark comparison. Lancet Infect Dis **2020**.

18. Royal College of Pathologists. Verification and validation methodology and sample sets for evaluation of assays for SARS-CoV-2 (COVID-19). Available here: https://www.rcpath.org/uploads/assets/541a4523-6058-4424-81c119dd2ab0febb/Verification-validation-of-sample-sets-assays-SARS-CoV-2.pdf. Accessed 23rd September 2020. .

19. Steyerberg EW, Eijkemans MJ, Harrell FE, Jr., Habbema JD. Prognostic modelling with logistic regression analysis: a comparison of selection and estimation methods in small data sets. Stat Med **2000**; 19(8): 1059-79.

20. Pavlou M, Ambler G, Seaman SR, et al. How to develop a more accurate risk prediction model when there are few events. BMJ **2015**; 351: h3868.

For peer review only - http://bmjopen.bmj.com/site/about/guidelines.xhtml

21. Anderson M, Pitchforth E, Asaria M, et al. LSE-Lancet Commission on the future of the NHS: re-laying the foundations for an equitable and efficient health and care service after COVID-19. Lancet **2021**; 397(10288): 1915-78.

22. The L. India's COVID-19 emergency. Lancet **2021**; 397(10286): 1683.

23. Woolf SH, Chapman DA, Sabo RT, Zimmerman EB. Excess Deaths From COVID-19 and Other Causes in the US, March 1, 2020, to January 2, 2021. JAMA **2021**.

24. Lemos DRQ, D'Angelo SM, Farias L, et al. Health system collapse 45 days after the detection of COVID-19 in Ceara, Northeast Brazil: a preliminary analysis. Rev Soc Bras Med Trop **2020**; 53: e20200354.

25. Kontopantelis E, Mamas MA, Webb RT, et al. Excess deaths from COVID-19 and other causes by region, neighbourhood deprivation level and place of death during the first 30 weeks of the pandemic in England and Wales: A retrospective registry study. Lancet Reg Health Eur **2021**; 7: 100144.

26. Nguyen LH, Drew DA, Joshi AD, et al. Risk of COVID-19 among frontline healthcare workers and the general community: a prospective cohort study. medRxiv **2020**.

27. Shah ASV, Wood R, Gribben C, et al. Risk of hospital admission with coronavirus disease 2019 in healthcare workers and their households: nationwide linkage cohort study. BMJ **2020**; 371: m3582.

28. Walker AS, Pritchard E, House T, et al. Ct threshold values, a proxy for viral load in community SARS-CoV-2 cases, demonstrate wide variation across populations and over time. Elife **2021**; 10.

29. Morales DR, Ali SN. COVID-19 and disparities affecting ethnic minorities. Lancet **2021**; 397(10286): 1684-5.

30. Mathur R, Rentsch CT, Morton CE, et al. Ethnic disparities in COVID-19: increased risk of infection or severe disease? - Authors' reply. Lancet **2021**; 398(10298): 390.

31. Eyre DW, Lumley SF, O'Donnell D, et al. Differential occupational risks to healthcare workers from SARS-CoV-2 observed during a prospective observational study. Elife **2020**; 9.

For peer review only

32. Howard-Anderson JR, Adams C, Sherman AC, et al. Occupational risk factors for severe acute respiratory coronavirus virus 2 (SARS-CoV-2) infection among healthcare personnel: A cross-sectional analysis of subjects enrolled in the COVID-19 Prevention in Emory Healthcare Personnel (COPE) study. Infect Control Hosp Epidemiol **2022**; 43(3): 381-6.

33. Baker JM, Nelson KN, Overton E, et al. Quantification of Occupational and Community Risk Factors for SARS-CoV-2 Seropositivity Among Health Care Workers in a Large U.S. Health Care System. Ann Intern Med **2021**; 174(5): 649-54.

34. Correia RF, da Costa ACC, Moore D, et al. SARS-CoV-2 seroprevalence and social inequalities in different subgroups of healthcare workers in Rio de Janeiro, Brazil. Lancet Reg Health Am **2022**; 7: 100170.

35. Brousseau N, Morin L, Ouakki M, et al. SARS-CoV-2 seroprevalence in health care workers from 10 hospitals in Quebec, Canada: a cross-sectional study. CMAJ **2021**; 193(49): E1868-E77.

36. Stead D, Adeniyi OV, Singata-Madliki M, et al. Cumulative incidence of SARS-CoV-2 and associated risk factors among healthcare workers: a cross-sectional study in the Eastern Cape, South Africa. BMJ Open **2022**; 12(3): e058761.

37. Ferris M, Ferris R, Workman C, et al. Efficacy of FFP3 respirators for prevention of SARS-CoV-2 infection in healthcare workers. Elife **2021**; 10.

38. Cambridge University Hospital NHS Foundation Trust. Infection Control Anuual Report 2020-2021. https://buckup-cuh-production.s3.amazonaws.com/documents/Infection_Control_Annual_Report_2020-21_final_SaUbrqH.pdf, **2021**.

39. Gray WK, Navaratnam AV, Day J, et al. Variability in COVID-19 in-hospital mortality rates between national health service trusts and regions in England: A national observational study for the Getting It Right First Time Programme. EClinicalMedicine **2021**; 35: 100859.

40. Ferry AV, Wereski R, Strachan FE, Mills NL. Predictors of UK healthcare worker burnout during the COVID-19 pandemic. QJM **2021**; 114(6): 374-80.

For peer review only - http://bmjopen.bmj.com/site/about/guidelines.xhtml

41. Kwon S, Joshi AD, Lo CH, et al. Association of social distancing and face mask use with risk of COVID-19. Nat Commun **2021**; 12(1): 3737.

42. Chu DK, Akl EA, Duda S, et al. Physical distancing, face masks, and eye protection to prevent person-to-person transmission of SARS-CoV-2 and COVID-19: a systematic review and meta-analysis. Lancet **2020**; 395(10242): 1973-87.

43. Thomas P, Baldwin C, Bissett B, et al. Physiotherapy management for COVID-19 in the acute hospital setting: clinical practice recommendations. J Physiother **2020**; 66(2): 73-82.

44. Dashti H, Roche EC, Bates DW, Mora S, Demler O. SARS2 simplified scores to estimate risk of hospitalization and death among patients with COVID-19. Sci Rep **2021**; 11(1): 4945.

45. Ellis G, Sevdalis N. Understanding and improving multidisciplinary team working in geriatric medicine. Age Ageing **2019**; 48(4): 498-505.

46. Cooper DJ LS, Watson L, Shaw A, Ferris M, et al. A prospective study of risk factors associated with seroprevalence of SARS-CoV-2 antibodies in healthcare workers at a large UK teaching hospital. J Infect **2022**.

47. Vivaldi G, Jolliffe DA, Holt H, et al. Risk factors for SARS-CoV-2 infection after primary vaccination with ChAdOx1 nCoV-19 or BNT1262b2 and after booster vaccination with BNT1262b2 or mRNA-1273: a population-based cohort study (COVIDENCE UK). medRxiv **2022**: 2022.03.11.22272276.

For peer review only

For peer review only - http://bmjopen.bmj.com/site/about/guidelines.xhtml

Table 1: Participant characteristics

Baseline variable	n (%)
Sex (male)	400/2044 (20)
Age (years), median (IQR)	42 (32 – 53)
Ethnicity	
- White British	1584 (70)
- White Irish	35 (1.6)
- White (other)	294 (13)
- Asian or Asian British (Indian)	70 (3.1)
- Asian or Asian British (Pakistani)	8 (0.4)
- Asian or Asian British (Bangladeshi)	2 (0.1)
- Asian or Asian British – Other	116 (5.1)
- Black or Black British (caribbean)	7 (0.3)
- Black or Black British (African)	29 (1.3)
- Black or Black British (Other)	1 (0.04)
- Mixed – White and Black Caribbean	6 (0.3)
- Mixed – White and Black African	9 (0.4)
- Mixed – White and Asian	12 (0.5)
- Mixed – Other	10 (0.4)
- Chinese	27 (1.2)
- Any other ethnic group	26 (1.2)
- Not stated	19 (0.8)
Occupation	
- Administrative staff	336 (14.9)
- Staff nurse	298 (13.2)
- Senior nursing staff	311 (13.8)
- Consultant	150 (6.6)
- Junior doctor	88 (3.9)

For peer review only

- Laboratory staff	141 (6.3)
- Healthcare assistant	189 (8.4)
- Theatre staff	25 (1.1)
- Manager	118 (5.2)
- Radiographer	62 (2.8)
- Midwife	65 (2.9)
- Physio	36 (1.6)
- Pharmacy staff	51 (2.3)
- Cleaning/domestic staff	6 (0.3)
- Dietician	23 (1)
- Occupational therapist	16 (0.7)
- Speech and Language therapist	20 (0.9)
- Porter	7 (0.3)
- Other	314 (13.9)
COVID working	618 / 2235 (28)

For peer review only - http://bmjopen.bmj.com/site/about/guidelines.xhtml

Table 2 – Significant Univariate analysis variables

	Variable	Odds Ratio[a]	95 % CI	p-value	n (positive) / N (responses) (%)
**Demographic**					
	Rent room in shared house	1.84	1.22 – 2.74	0.003	33/209 (16)
	Live with other HCWs	1.49	1.10 – 2.02	0.009	70/550 (13)
	Children attended school in June	0.58	0.35 – 0.97	0.038	30/395 (7.6)
	Household member +ve PCR test	3.48	2.09 – 5.78	<0.0001	22/84 (26)
	Household member +ve Ab test	11.29	7.08 – 18.01	<0.0001	40/79 (51)
	Household member symptomatic	3.71	2.8 – 4.96	<0.0001	95/437 (22)
	Born in UK	0.59	0.44 – 0.79	<0.001	136/1616 (8.4)
	Ethnicity	1.06	1.03 – 1.10	<0.001[b]	1584/2258[c]
**Occupational**					
	Job role				
	- Admin staff	1	-	-	24/336 (7)
	- Staff nurse	2.02	1.18 – 3.43	0.01	40/298 (13.4)
	- Physiotherapist	4.33	1.83 – 10.25	0.001	9/36 (25)
	Direct patient care COVID	1.86	1.41 – 2.47	<0.001	103/757 (13.6)
	Worked in red area	1.78	1.33 – 2.38	<0.001	85/618 (13.8)
	Specialty				
	- Non-patient facing roles	1	-	-	10/169 (6)
	- Critical care	2.51	1.10 – 5.77	0.029	16/117 (13.7)
	- Acute med	4.57	2.08 – 10.07	<0.001	23/103 (22.3)
	- Medical specialties	4.35	2.01 – 9.42	<0.001	26/121 (21.5)
	- Surgical	2.71	1.24 – 5.93	0.012	22/151 (14.6)
	Night shifts	1.68	1.26 – 2.25	<0.001	82/604 (13.6)
	Receive formal PPE training	1.40	1.05 – 1.85	0.02	129/1141 (11.3)
	Adequate PPE available				

For peer review only - http://bmjopen.bmj.com/site/about/guidelines.xhtml

	- All of the time	1	-	-	83/1038 (8)
	- Most of the time	1.34	0.98 – 1.83	0.065	92/882 (10.4)
	- Some of the time	1.93	1.22 – 3.05	0.005	28/195 (14.4)
	- Rarely	3.60	1.71 – 7.57	0.001	10/42 (23.8)
	Rest/meal with colleagues				
	- Never	1	-	-	
	- Most of the time	1.99	1.19 – 3.33	0.009	[d]
	Use doctors mess	1.77	1.17 – 2.69	0.007	30/195 (15.4)
	Hospital supplied scrubs	1.15	1.04 – 1.27	0.007	
	Work from home - March	0.60	0.39 – 0.91	0.016	
	Work from home – June	0.58	0.36 – 0.94	0.026	
	Handover w/o social distancing	1.74	1.31 – 2.30	<0.0001	
**Behavioural**					
	Smoker	0.37	0.18 – 0.76	0.007	
	Food deliveries march				
	- Daily	5.38	1.27 – 22.8	0.022	[e]
	Food deliveries June				
	- Daily	6.10	1.01 – 36.7	0.049	[e]
	Exercise outdoors March				
	- Daily	0.58	0.39 – 0.86	0.007	[e]
	Exercise outdoors June				
	- Daily	0.56	0.37 – 0.84	0.005	[e]

[a] Unadjusted Odds Ratio

[b] p-value for likelihood ratio test

[c] number of participants identifying as white British

[d] Compared to “never”

[e] Compared to < once per week.

For peer review only - http://bmjopen.bmj.com/site/about/guidelines.xhtml

Table 3 – Final multivariate model

Variable	OR	95% CI	p-value
Household positive antibody	6.94	4.15 – 11.6	<0.001
Household positive symptoms	2.95	2.13 – 4.08	<0.001
Black ethnicity	6.21	2.69 – 14.3	<0.001
Physiotherapist	2.78	1.21 – 6.39	0.015
Acute medicine specialty	2.72	1.57 – 4.69	<0.001
Medical specialties	2.33	1.40 – 3.88	0.001
Inadequate PPE	2.84	1.29 – 6.25	0.010
Handover w/o distancing	1.39	1.02 – 1.90	0.038

For peer review only

For peer review only - http://bmjopen.bmj.com/site/about/guidelines.xhtml

Figure 1: Flow chart of study procedures.

Figure 2: Forest plot of final multivariate model.

For peer review only

For peer review only - http://bmjopen.bmj.com/site/about/guidelines.xhtml

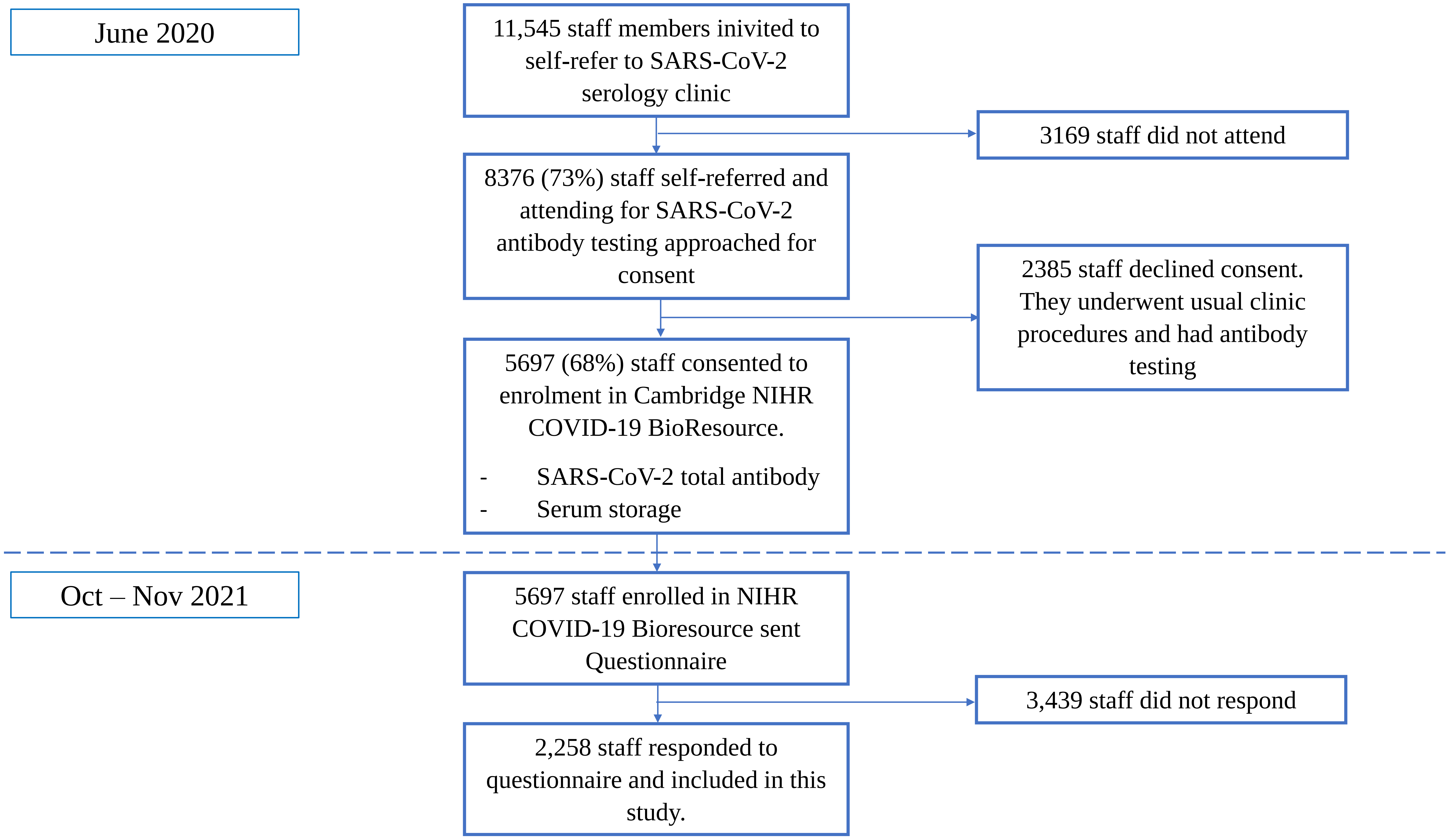
June 2020
11,545 staff members inivited to self-refer to SARS-CoV-2 serology clinic
3169 staff did not attend
8376 (73%) staff self-referred and attending for SARS-CoV-2 antibody testing approached for consent
2385 staff declined consent. They underwent usual clinic procedures and had antibody testing
5697 (68%) staff consented to enrolment in Cambridge NIHR COVID-19 BioResource.
- SARS-CoV-2 total antibody
- Serum storage
Oct – Nov 2021
5697 staff enrolled in NIHR COVID-19 Bioresource sent Questionnaire
3,439 staff did not respond
2,258 staff responded to questionnaire and included in this study.

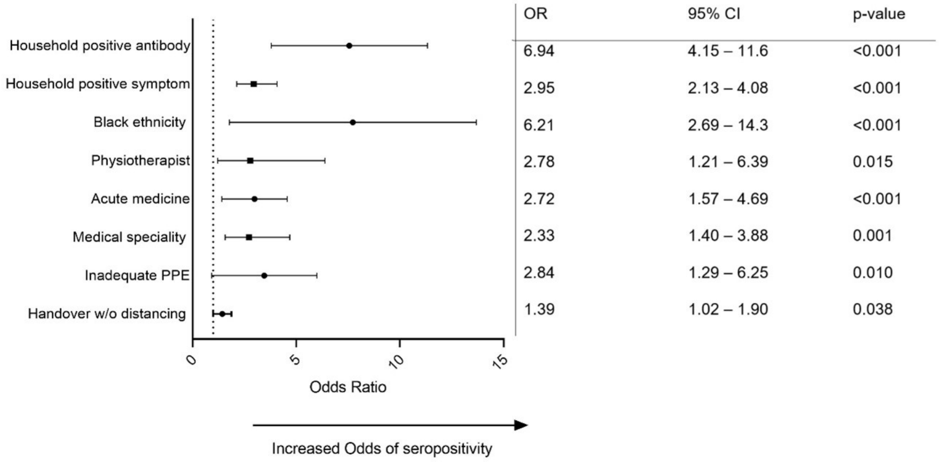

Figure 2: Forest plot of final multivariate model.

159x80mm (220 x 220 DPI)

Univariate logistic regression tables of variables assessed

A. *Demographic factors and association with risk of SARS-CoV-2 infection in HCWs.*

**Variable**	**OR**	**95 % CI**	***p*-value**	n (positive) / N (responses) (%)
Number in househould (1)	1	-	-	14/217 (6.5)
- 2	1.64	0.91 – 2.96	0.10	78/768 (10.1)
- 3	1.43	0.76 – 2.68	0.26	42/468 (9.0)
- 4	1.59	0.86 – 2.92	0.14	55/557 (9.9)
- 5	2.43	0.97 – 4.69	0.09	19/160 (11.9)
- 6	2.11	0.81 – 5.52	0.13	7/55 (12.7)
- 7	0.81	0.10 – 6.48	0.84	1/19 (5.2)
- 8	4.8	0.47 – 49.5	0.19	1/4 (25)
- 9	1	-	-	0/5 (0)
Rent room in shared house	1.84	1.22 – 2.74	0.003	33/209 (16)
Live with other HCWs	1.49	1.10 – 2.02	0.009	70/550 (13)
Live other key workers (not HCWs)	0.95	0.70 – 1.29	0.73	63/663 (9.5)

For peer review only

Multigenerational household	0.96	0.73 – 1.27	0.79	104/1073 (9.7)
Children in household	1.13	0.85 – 1.49	0.40	99/944 (10.5)
Number of children				
- 0	1	-	-	126/1331 (9.5)
- 1				
- 2	1.04	0.72 – 1.50	0.84	43/439 (9.8)
- 3	1.28	0.70 – 2.35	0.42	13/110 (11.8)
- 4	1.91	0.41 – 8.83	0.41	2/12 (16.7)
- 5	2.39	0.27 – 21.6	0.44	1/5 (20)
School aged children	0.97	0.72 – 1.32	0.86	66/684 (9.7)
Children attended school in March	1.52	0.90 – 2.56	0.12	27/225 (12)
Children attended school in June	0.58	0.35 – 0.97	0.038	30/395 (7.6)
Nursery age children	0.69	0.38 – 1.23	0.20	13/183 (7.1)
Children attend nursery March	1.91	0.56 – 6.51	0.30	6/72 (8.3)
Children attend nursery June	1.05	0.31 – 3.55	0.94	9/125 (7.2)
People >65 in household	1.05	0.65 – 1.68	0.86	21/207 (10.1)
Household member positive PCR test	3.48	2.09 – 5.78	<0.0001	22/84 (26)

For peer review only

Household member positive Ab test	11.29	7.08 – 18.01	<0.0001	40/79 (51)
Household member symptomatic	3.71	2.8 – 4.96	<0.0001	95/437 (22)
Travel to work				
- Drive	1	-	-	133/1449 (9.2)
- Walk	1.28	0.78 – 2.10	0.34	20/175 (11.4)
- Cycle	1.19	0.84 – 1.69	0.33	47/438 (10.7)
- Bus	1.15	0.63 – 2.10	0.65	13/125 (10.4)
- Train	1.62	0.75 – 3.48	0.22	8/57 (14.0)
Share a car	1.49	0.84 – 2.61	0.17	15/109 (13.8)

For peer review only

B. Socioeconomic *factors and association with risk of SARS-CoV-2 infection in HCWs.*

**Variable**	**OR**	**95 % CI**	***p*-value**	n (positive) / N (responses) (%)
Born in UK	0.59	0.44 – 0.79	<0.001	136/1616 (8.4)
Ethnicity	1.06	1.03 – 1.10	<0.001[a]	1584/2258[b]
Highest level of education				
- Higher degree	1	-	-	84/869 (9.7)
- GCSE	1.02	0.61 – 1.71	0.94	20/203 (9.9)
- A level	0.90	0.54 – 1.51	0.70	20/227 (8.8)
- Undergraduate degree	1.15	0.83 – 1.59	0.40	79/722 (10.9)
- Other vocational training	0.75	0.44 – 1.30	0.31	17/228 (7.5)
More than one job	0.95	0.61 – 1.49	0.84	24/254 (9.5)
Other dependents	1.21	0.63 – 2.30	0.57	11/95 (11.6)
Care outside of household	0.57	0.34 – 0.11	0.056	19/232 (8.2)

[a] p-value for likelihood ratio test

[b] number of participants identifying as white British

For peer review only

C. Occupational factors and association with risk of SARS-CoV-2 infection in HCWs.

Variable	OR	95 % CI	p-value	n (positive) / N (responses) (%)
Job role				
- Administrative staff	1	-	-	24/336 (7.1)
- Staff nurse	2.02	1.18 – 3.43	0.01	40/298 (13.4)
- Senior nursing staff	1.54	0.89 – 2.67	1.55	33/311 (10.6)
- Consultant	1.66	0.86 – 3.19	0.13	17/150 (11.3)
- Junior doctor	1.67	0.77 – 3.63	0.20	10/88 (11.4)
- Laboratory staff	0.58	0.23 – 1.44	0.24	6/141 (4.3)
- Healthcare assistant	1.71	0.93 – 3.15	0.08	22/189 (11.6)
- Theatre staff	0.54	0.07 – 4.18	0.56	1/25 (4)
- Manager	1.75	0.87 – 3.51	0.12	14/118 (11.9)
- Radiographer	1.39	0.54 – 3.56	0.69	6/62 (9.7)
- Midwife	0.20	0.27 – 1.53	0.12	1/65 (1.5)

For peer review only

- Physio	4.33	1.83 – 10.25	0.001	9/36 (25)
- Pharmacy staff	2.07	0.84 – 5.08	0.11	7/51 (13.7)
- Cleaning/domestic staff	1	-	-	0/6 (0)
- Dietician	0.59	0.076 – 4.57	0.61	1/23 (4.4)
- Occupational therapist	0.87	0.11 – 6.84	0.89	1/16 (6.25)
- Speech and Language therapist	2.29	0.63 – 8.38	0.48	3/20 (15)
- Porter	2.17	0.25 – 18.7	0.48	1/7 (14.3)
- Other	1.17	0.05 – 0.12	0.59	26/314 (8.3)
Direct patient care COVID	1.86	1.41 – 2.47	<0.001	103/757 (13.6)
Worked in red area	1.78	1.33 – 2.38	<0.001	85/618 (13.8)
Time in red area	0.99	0.83 – 1.20	0.99	-
Specialty				
- Non-patient facing	1	-	-	10/169 (5.9)
- Emergency department	1.32	1.10 – 5.77	0.44	44/574 (7.7)
- Critical care	2.51	1.10 – 5.77	0.029	16/117 (13.7)
- Acute medicine	4.57	2.08 – 10.07	<0.001	23/103 (22.3)
- Respiratory medicine	2.0	0.51 – 7.74	0.32	3/27 (11.1)

For peer review only

- Infectious diseases	1.59	0.32 – 7.78	0.57	2/22 (9.1)
- Medical specialties	4.35	2.01 – 9.42	<0.001	26/121 (21.5)
- Theatres	2.01	0.80 – 5.04	0.14	10/89 (11.2)
- ENT	0.66	0.08 – 5.41	0.70	1/25 (4)
- Surgical	2.71	1.24 – 5.93	0.012	22/151 (14.6)
- Paediatrics	0.71	0.24 – 2.13	0.54	5/117 (4.3)
- Research	1.44	0.64 – 3.25	0.37	17/204 (8.3)
- Other	1.27	0.61 – 2.66	0.53	11/101 (10.9)
Average hours per week March	1.02	0.96 – 1.09	0.49	-
Average hours per week June	0.99	0.93 – 1.06	0.84	-
Work nights	1.68	1.26 – 2.25	<0.001	82/604 (13.6)
Present for AGPs	1.30	0.93 – 1.84	0.13	47/396 (11.9)
Receive formal PPE training	1.40	1.05 – 1.85	0.02	129/1141 (11.3)
Adequate PPE available				
- All of the time	1	-	-	83/1038 (8.0)
- Most of the time	1.34	0.98 – 1.83	0.065	92/882 (10.4)
- Some of the time	1.93	1.22 – 3.05	0.005	28/195 (14.4)

For peer review only

- Rarely	3.60	1.71 – 7.57	0.001	10/42 (23.8)
Use mask at work before widespread	0.98	0.86 – 1.12	0.79	127/1198 (10.6)
Which mask when mandatory	0.94	0.81 – 1.10	0.45	-
What type of eye protection	1.05	0.97 – 1.14	0.25	-
Rest/meal with colleagues				
- Never	1	-	-	21/297 (7.1)
- All of the time	1.49	0.81 – 2.76	0.20	24/235 (10.2)
- Most of the time	1.99	1.19 – 3.33	0.009	64/487 (13.1)
- Some of the time	1.52	0.92 – 2.51	0.10	76/733 (10.4)
- Rarely	1.05	0.60 – 1.85	0.86	35/472 (7.4)
Eat in staff canteen	1.08	0.99 – 1.17	0.06	-
Shared rest areas	0.99	0.90 – 1.11	0.96	-
Use doctors mess	1.77	1.17 – 2.69	0.007	30/195 (15.4)
Hospital supplied scrubs	1.15	1.04 – 1.27	0.007	124/1056 (11.7)
Own scrubs	1.04	0.88 – 1.23	0.62	26/256 (10.2)
Own clothes to work	0.95	0.80 – 1.13	0.54	165/1684 (9.8)
Use changing room at work	1.04	0.92 – 1.18	0.52	139/1323 (10.5)

For peer review only

Dedicated footwear for work	1.14	0.97 – 1.33	0.11	151/1390 (10.9)
Wear own clothes when going home	1.00	0.87 – 1.16	0.95	90/937 (9.6)
Re-usable water bottle	1.15	0.98 – 1.29	0.56	178/1760 (10.1)
Adherence to handwashing technique	1.15	0.88 – 1.51	0.31	-
Handwashing frequency	1.00	0.76 – 1.32	0.99	-
Work from home March	0.60	0.39 – 0.91	0.016	27/410 (6.6)
- For shielding?	0.58	0.36	0.94	4/62 (6.5)
Work from home June	0.58	0.36 – 0.94	0.026	2-/314 (6.4)
- For shielding?	1.81	0.63 – 5.22	0.27	5/50 (10)

For peer review only

D. Behavioural factors and association with risk of SARS-CoV-2 infection in HCWs.

**Variable**	**OR**	**95 % CI**	**p-value**	**n (positive) / N (responses) (%)**
Smoker	0.37	0.18 – 0.76	0.007	8/196 (4.1)
Quantity smoked	0.54	0.20 – 1.48	0.23	-
Alcohol	0.74	0.55 – 0.98	0.038	78/864 (4.1)
Frequency of alcohol	0.91	0.66 – 1.24	0.54	-
Shopping frequency March	0.91	0.75 – 1.10	0.32	-
Shopping frequency June	0.90	0.74 – 1.08	0.26	-
Contact with people March	1.04	0.86 – 1.25	0.72	-
Contact with people June	1.06	0.90 – 0.25	0.50	-
Food deliveries march				
- Less than once/week	1	-	-	141/1406 (10.0)
- Once a week	0.96	0.70 – 1.31	0.81	64/661 (9.7)
- 2-3 times / week	0.68	0.35 – 1.31	0.25	10/143 (7.0)
- Daily	5.38	1.27 – 22.8	0.022	3/8 (37.5)
Food deliveries June				

For peer review only

- Less than once/week	1	-	-	136/1376 (9.9)
- Once a week	0.96	0.70 – 1.30	0.78	66/695 (9.5)
- 2-3 times / week	1.01	0.58 – 1.78	0.96	15/150 (10)
- Daily	6.10	1.01 – 36.7	0.049	2/5 (40)
Exercise outdoors March				
- Less than once/week	1	-	-	53/428 (12.4)
- Once a week	1.07	0.69 – 1.66	0.77	40/305 (13)
- 2-3 times / week	0.73	0.50 – 1.06	0.10	72/770 (9.4)
- Daily	0.58	0.39 – 0.86	0.007	54/718 (7.5)
Exercise outdoors June				
- Less than once/week	1	-	-	51/405 (12.6)
- Once a week	0.89	0.57 – 1.41	0.63	35/307 (11.4)
- 2-3 times / week	0.76	0.52 – 1.10	0.14	79/804 (9.8)
- Daily	0.56	0.37 – 0.84	0.005	53/709
Public transport March	0.81	0.64 – 1.04	0.10	-
Public transport June	0.80	0.63 – 1.01	0.06	-
Facemask March	0.99	0.90 – 1.10	0.86	-

For peer review only

Facemask June	1.0	0.88 – 1.13	1.0	-
No social distancing March	1.74	1.31 – 2.30	<0.0001	127/1021 (12.4)
No social distancing June	1.31	1.0 – 1.73	0.06	105/933 (11.3)
Work duties altered for risk	1.12	0.75 – 1.69	0.58	40/278 (10.8)

For peer review only

E. Health factors and association with risk of SARS-CoV-2 infection in HCWs.

Variable	OR	95 % CI	p-value	n (positive) / N (responses) (%)
Told overweight	0.88	0.63 – 1.23	0.46	49/543 (9.0)
Told obese	0.93	0.58 – 1.49	0.77	21/225 (9.3)
Exercise frequency				
- 2-3 times/week	1	-	-	101/919 (11.0)
- Daily	0.67	0.46 – 0.97	0.033	43/566 (7.6)
- Once a week	0.91	0.61 – 1.35	0.64	37/367 (10.1)
- < once a week	0.91	0.61 – 1.34	0.63	39/387 (10.1)
Heart disease	1.17	0.41 – 3.34	0.77	4/35 (11.4)
Lung disease	0.74	0.36 – 1.55	0.43	8/106 (7.6)
Kidney disease	-	-	-	0/10 (0)
High BP	0.75	0.46 – 1.23	0.26	19/244 (7.8)
- BP medicated	1.03	0.35 – 1.97	0.96	14/175 (8.0)
- Medication #	0.89	0.55 – 1.46	0.65	-
T1 DM	2.01	0.43 – 9.38	0.37	2/11 (18.2)
T2 DM	1.17	0.46 – 3.02	0.74	5/44 (11.4)

For peer review only

- Insulin	-	-	-	0/3 (0)
- Medication #	0.75	0.29 – 1.91	0.54	-
Immunosuppression	1.3	0.51 – 3.36	0.59	5/40
Blood disorder	1.84	0.40 – 8.4	0.43	2/12 (16.7)
Inherited	1.65	0.36 – 7.51	0.52	2/13 (15.4)
Organ transplant	-	-	-	0/0 (0)
Cancer treatment	-	-	-	0/9 (0)
Currently taking:				
- Hydroxychloroquine	0.48	0.06 – 3.6	0.48	1/20 (5.0)
- Aspirin	1.0	0.58 – 1.74	0.99	15/152 (9.9)
- ACE inhibitors	1.52	0.74 – 3.12	0.25	9/64 (14.1)
- ARBs	0.74	0.23 – 2.42	0.62	3/40 (7.5)
- Tacrolimus	2.30	0.26 – 20.66	0.46	1/5 (20)
- Mycophenolate	2.30	0.26 – 20.66	0.46	1/5 (20)
- Prednisolone	1.59	0.61 – 4.16	0.34	5/34 (14.7)
- Tocilizumab	1.84	0.21 – 15.8	0.58	1/6 (16.7)
- Azathioprine	2.31	0.49 – 10.92	0.29	2/10 (20)

For peer review only

- Methotrexate	1.15	0.26 – 5.03	0.86	2/18 (11.1)
- Cyclosporine	2.30	0.26 – 20.66	0.46	1/5 (20)
- Leflunomide	1.53	0.18 – 12.77	0.69	1/7 (14.3)
Ever had:				
- Rituximab	0.83	0.11 – 6.48	0.86	1/12 (8.3)
- Abatacept	2.31	0.26 – 20.66	0.46	1/5 (20)
- Adalimumab	1.15	0.14 – 9.21	0.90	1/9 (11.1)
- Etanercept	0.83	0.11 – 6.48	0.86	1/12 (8.3)
- Infliximab	2.31	0.49 – 10.92	0.29	2/10 (20)
- Basiliximab	2.30	0.26 – 20.66	0.46	1/5 (20)
- Cyclophosphamide	0.76	0.10 – 5.90	0.80	1/13 (7.7)
Chemotherapy for cancer	0.65	0.20 – 2.11	0.47	3/45 (6.7)

For peer review only

A. Demographics

1. How many people live in your household (select number)
2. Do you rent a room in a shared house (yes/no)
3. Do you live with other healthcare workers (yes/no)
4. Do you live with other key workers (who are not healthcare workers) who have worked during this time? (yes/no)
5. Is there more than one generation of your family living in your household (e.g. Children, parents or grandparents) (yes/no) If yes (may be multiple):

- Children
- Parents
- Grandparents
- More than one of the above
- Other (free text)

6. Are there children living in your house? (yes/no)

- If yes, how many.
- What ages (select from drop down list for each child – multiple depending on how many entered in answer above if possibly [– *on REDCap, or provide boxes for paper form*])

7. Do you have school aged children? (yes/no). If yes:

- Did they attend school between March to May 2020? (yes/no)
- Did they attend school between from June to July 2020? (yes/no)

8. Do you have children who attend nursery. (yes/no). If yes:

- Did they attend nursery between March to May 2020? (yes/no)
- Did they attend nursery between from June to July 2020? (yes/no)

9. Is there anyone in your household who is >65 years old? (yes/no)
10. Did anybody in your household (other than yourself) test positive on a throat swab (PCR test) for COVID-19?
11. Did anybody in your household (other than yourself) test positive on a blood test (antibody test) for COVID-19?
12. Did anybody (other than yourself) have symptoms consistent with COVID-19 between February and July 2020?
13. How do you travel to and from work? (drop down list)

- Walk/run; cycle; personal car; bus; train

14. If you drive, do you share lifts with other healthcare workers who aren't in your immediate household? (yes/no)

B. Socioeconomic

1. Were you born in the United Kingdom? (yes/no)

For peer review only

2. Ethnicity (drop down list) *[– insert List of NHS ethnicity codes A-Z]*
3. What is your highest level of education? (Select from list: GCSE; A level; Undergraduate degree; higher degree; other vocational training)
4. Have you been employed in more than one job during this time? (yes/no)
5. Do you have any dependents other than your immediate family members? (yes/no)
6. Do you provide care for anyone outside of your immediate household? (including washing/dressing, cooking, shopping, cleaning, healthcare needs) (yes/no)

C. Occupational

1. What is your job role?

- Admin or reception staff
- Staff nurse
- Nursing Sister / Senior nursing staff
- Consultant
- Junior doctor (including FY1/FY2/Core trainee/Speciality Trainee)
- Laboratory staff
- Healthcare Assistant
- Operating department staff
- Manager
- Radiographer
- Midwife
- Physiotherapist / Physiotherapy assistant
- Pharmacy staff
- Cleaning/domestic staff
- Dietician
- Occupational therapist
- SALT
- Porter
- Other (FREE TEXT)

2. Please select all areas you have worked during this time (may be multiple):

- Ward A2 - Neurosciences critical care unit (NCCU)
- Ward J2 - Trauma high dependency unit
- Ward A4 - Neurology / Neurosurgery
- Ward A5 - Neuro-oncology / Neurosurgery
- Ward C2 - Children's oncology and haematology
- Ward C3 - Children’s surgical and medicine
- Ward C4 - Frail and Acute Medicine for the Elderly
- Ward C5 - General medicine and nephrology
- Ward C6 - Medicine for the elderly
- Ward C7 - Gastroenterology
- Ward C8 - Surgical Admissions for ‘Amber’ patients

For peer review only

- Ward C9 - Teenage Cancer Trust Unit
- Ward C10 - Haematology and haematological oncology
- Ward D2 - Children's surgical and medicine
- Ward D3 - John Farman intensive care unit
- Ward D4 - Intermediate dependancy area
- Ward D5 – DME Medicine for the elderly
- Ward D6 - Neuro/Stroke/ Neurosurgery/Gastro Haematology
- Ward D7 - Diabetes and endocrinology
- Ward D9 - Oncology
- Ward D10 - Respiratory
- Ward EAU 2 - Paediatric Emergency Department
- Ward EAU 3 - Ambulatory care
- Ward EAU 4 - Acute Hub - Green Medical Admissions/Short Stay
- Ward EAU 5 - Acute Hub - Red Medicine
- Ward F2 - Inpatient Occupational Therapy
- Ward F3
- Ward F4 - Renal
- Ward F5 - Transplant high dependency unit
- Ward F6 - Trauma and Orthopaedics
- Ward G2 - Infusion services
- Ward G3 - Diabetes, I.D. and Oncology
- Ward G4 - Hepatology
- Ward G5 - Transplant unit
- Ward G6 - Medicine for the elderly
- Ward J2 - Major trauma unit
- Ward J3 - Post Anaesthetic Care Unit (PACU) and 23 Hour Stays
- Ward K2 - Cardiology
- Ward K3 - Cardiology and coronary care unit
- Ward L2 - Day surgery unit
- Ward L4 - Non-Elective Surgery Patients
- Ward L5 - Non-Elective Surgery Patients
- Ward M4 - Non-Elective Surgery Patients
- Ward M5 - Elective Surgery Patients
- Ward N2 -Amber Medical Admissions for Covid Pathway
- Ward N3 - Respiratory medicine
- Ward R3 - Neurosciences
- Ward S3 - Psychiatry
- Surgical Ambulatory Care Unit
- Clinical Investigation Ward (CIW)
- Clinical Research Facility (CRF)
- Coronary care unit (CCU)
- Haematology day unit
- Intermediate dependency area (IDA)
- Ward EAU 4 - Acute Hub - Green Medical Admissions/Short Stay
- Paediatric intensive care unit (PICU)
- Paediatric Day Unit (PDU)
- Stroke Unit - Ward R2 and Lewin rehabilitation unit
- Delivery unit

For peer review only

- Ward - Lady Mary - Postnatal
- Neonatal unit
- Ward - Sara - Antenatal
- Daphne ward – Gynaecology
- Ward - Charles Wolfson

3. Have you been involved in the direct patient care of patients with confirmed COVID-19? (yes/no)

4. Have you worked in a specified "Red" area between March and July 2020? (yes/no). If yes:

- Less than 1 week
- 1 week
- 1 week – 1 month
- >1 month

5. Which speciality have you predominantly worked in between March and July 2020?

- Emergency Department
- Critical Care
- Acute Medicine
- Respiratory Medicine
- Infectious Diseases
- Medicine (not including Respiratory or Infectious Diseases)
- Operating Department (Theatres)
- ENT
- Surgical specialties
- Paediatrics
- Research
- Non-patient facing role

6. How many hours did you work in the average week from March to May 2020?

7. How many hours did you work in the average week from June to July 2020?

8. Does your working pattern include night shifts? (yes/no)

9. Have you been present during aerosol generating procedures on COVID-19 confirmed patients? (yes/no). If yes:

- tracheal intubation and extubation
- manual ventilation
- tracheotomy or tracheostomy procedures (insertion or removal)
- bronchoscopy
- dental procedures (using high speed devices, for example ultrasonic scalers/high speed drills
- non-invasive ventilation (NIV); Bi-level Positive Airway Pressure Ventilation (BiPAP) and Continuous Positive Airway Pressure Ventilation (CPAP)
- high flow nasal oxygen (HFNO)
- high frequency oscillatory ventilation (HFOV)
- induction of sputum using nebulised saline

For peer review only

- respiratory tract suctioning
- upper ENT airway procedures that involve respiratory suctioning
- upper gastro-intestinal endoscopy where open suction of the upper respiratory tract occurs
-

10. Did you receive formal PPE training? (yes/no)

11. Did you feel that adequate PPE was available to you:

- At all times
- Most of the time
- Some of the time
- Rarely

12. Prior to the introduction of hospital-wide surgical-resistant masks, which type of facemask did you predominantly use at work?

- None
- Water resistant surgical mask
- FFP3
- Respirator hood
- Other respirator
- Other

13. After the introduction of hospital-wide surgical-resistant masks, which type of facemask did you predominantly use at work?

- None
- Water resistant surgical mask
- FFP3
- Respirator hood
- Other respirator
- Other

14. What type of eye protection did you predominantly use at work:

- None
- Own spectacles/glasses
- Protective glasses (hospital supplied)
- Goggles
- Face shield

15. Did you take rest/meal breaks at the same time as colleagues?

- All of the time
- Most of the time
- Some of the time
- Rarely
- Never

16. Did you eat in the staff canteen?

- All of the time
- Most of the time

For peer review only

- Some of the time
- Rarely
- Never

17. Did you use shared rest facilities in your primary area of work (e.g. tea/break room)?

- All of the time
- Most of the time
- Some of the time
- Rarely
- Never

18. Did you use the doctors' mess during this time?

- All of the time
- Most of the time
- Some of the time
- Rarely
- Never

19. Did you wear hospital supplied scrubs at work?

- All of the time
- Most of the time
- Some of the time
- Rarely
- Never

20. Did you wear your own scrubs at work?

- All of the time
- Most of the time
- Some of the time
- Rarely
- Never

21. Did you wear your own clothes to work?

- All of the time
- Most of the time
- Some of the time
- Rarely
- Never

22. Did you use a changing room at work?

- All of the time
- Most of the time
- Some of the time
- Rarely
- Never

23. Did you have dedicated footwear for work during this time?

For peer review only

- All of the time
- Most of the time
- Some of the time
- Rarely
- Never

24. Did you wear your work clothes when leaving the hospital?

- All of the time
- Most of the time
- Some of the time
- Rarely
- Never

25. Did you use a reusable personal water/drinks bottle in your area of work?

- All of the time
- Most of the time
- Some of the time
- Rarely
- Never

26. How would you rate your adherence to trust policy hand-washing technique?

- All of the time
- Most of the time
- Some of the time
- Rarely
- Never

27. How would you rate your adherence to trust policy hand-washing frequency

- All of the time
- Most of the time
- Some of the time
- Rarely
- Never

28. Did you primarily work from home between March to May 2020?

- If yes, was this recommended for shielding reasons?

29. Did you primarily work from home from June to July 2020?

- If yes, was this recommended for shielding reasons?

30. Have you ever been recommended to shield by Occupational Health?

31. Have you ever been in a group that was recommended to shield by Public Health England? (yes/no)

D. Behavioural

For peer review only

1. Were you a smoker at any point between March to July 2020? (yes/no) If yes:

- Fewer than 5 per day
- 5-10 per day
- 10-20 per day
- >20 per day

2. Did you regularly drink alcohol between March to July 2020? (yes/no) If yes:

- Daily
- 2-3 times per week
- Once a week
- Less than once a week

3. How frequently did you visit a supermarket or shop between March to May 2020?

- Daily
- 2-3 times per week
- Once a week
- Less than once a week

4. How frequently did you visit a supermarket or shop between June to July 2020?

- Daily
- 2-3 times per week
- Once a week
- Less than once a week

5. How often did you have contact with people outside of your immediate household (not including work) between March to May 2020?

- Daily
- 2-3 times per week
- Once a week
- Less than once a week

6. How often did you have contact with people outside of your immediate household (not including work) between June to July 2020?

- Daily
- 2-3 times per week
- Once a week
- Less than once a week

7. How often did you order food deliveries (e.g. groceries, take-away) between March to May 2020?

- Daily
- 2-3 times per week
- Once a week

For peer review only

- Less than once a week

8. How often did you order food deliveries (e.g. groceries, take-away) between June to July 2020?

- Daily
- 2-3 times per week
- Once a week
- Less than once a week

9. How often did you exercise outdoors from March to May 2020?

- Daily
- 2-3 times per week
- Once a week
- Less than once a week

10. How often did you exercise outdoors June to July 2020?

- Daily
- 2-3 times per week
- Once a week
- Less than once a week

11. How often did you use public transport (not including travel to and from work) from March to May 2020?

- Daily
- 2-3 times per week
- Once a week
- Less than once a week

12. How often did you use public transport (not including travel to and from work) from June to July 2020?

- Daily
- 2-3 times per week
- Once a week
- Less than once a week

13. Did you use a facemask outside of work from March to May 2020?

- All of the time
- Most of the time
- Some of the time
- Rarely
- Never

14. Did you use a facemask outside of work from June to July 2020?

For peer review only

- All of the time
- Most of the time
- Some of the time
- Rarely
- Never

15. If you used a facemask outside of work, in which situations did you use one? (may be multiple)

- Social interaction
- Grocery shopping
- Commuting
- Exercising
- Other

16. Did you attend meetings or handovers where it was not possible to socially distance between March to May 2020? (yes/no)

17. Did you attend meetings or handovers where it was not possible to socially distance between June to July 2020? (yes/no)

E. Co-morbidities

1. What was your COVID risk-assessment group?

- Green
- Yellow
- Orange
- Red

2. Were your work duties altered because of your risk group? (yes/no)

3. Self-reported height [give measuring unit options ft/inches or m/cm]

4. Self-reported weight [give measuring usingt options st/lb or kg]

5. Have you even been told you are overweight in a medical setting? (yes/no)

6. Have you even been told you are obese in a medical setting? (yes/no

7. How often do you undertake physical exercise?

- Daily
- 2-3 times per week
- Once a week
- Less than once a week

8. Do you have any of the following co-morbidities:

Heart disease. (yes/no) If yes – select (may be multiple)

- Ischaemic heart disease
- Previous myocardial infarction (heart attack)
- Angina
- Valvular heart disease

For peer review only

- Other

Kidney disease. (yes/no) If yes – select

- Chronic kidney disease – not on dialysis
- Are you on haemodialysis?
- Are you on Peritoneal dialysis?
- Have you had a kidney transplant?
- Vasculitis
- Other

Lung disease. (yes/no) If yes – select (may be multiple)

- Chronic obstructive pulmonary disease (COPD)/ Chronic obstructive airway disease (COAD)
- Asthma
- Interstitial lung disease
- Bronchiectasis
- Emphysema
- Other

Have you ever been diagnosed with high blood pressure (yes/no). If yes:

- Are you on any medication. (yes/no)
- How many different medications (insert number)
- Is your blood pressure well controlled? (yes/no)

Type 1 diabetes (yes/no)

Type 2 diabetes (yes/no). If yes:

- Do you take insulin?
- How many medications do you take for diabetes? (must include zero)
- Is your blood sugar well controlled?

Do you have a compromised immune system due to any of the following?

- Immunosuppression drugs (yes/no)
- Blood disorder (including blood cancer) (yes/no)
- An inherited immune deficiency (yes/no)
- Other (free text) (yes/no)

Have you had a solid organ transplant (yes/no). If yes:

- Kidney
- Heart
- Lung
- Liver
- Small intestine
- Pancreas

Are you currently being treated for cancer? (yes/no). If yes:

- Solid organ cancer
- Blood cancer

For peer review only

- Skin cancer
- Other

9. Have you taken hydroxychloroquine at any time between March to July 2020? (yes/no). If yes:

- More than once daily
- Once daily
- 2 – 6 times a week
- Once a week
- Less than once a week

10. Did you take any of the following medication between March and July 2020 (may be multiple):

- Aspirin
- Angiotensin converting enzyme (ACE) inhibitors (including ramipril, lisinopril, captopril, enalopril and others)
- Angiotensin receptor blockers (ARBs) (including candesartan, irbesartan, losartan, valsartan and others)
- Tacrolimus
- Mycophenolate
- Hydroxychloroquine
- Prednisolone
- Tocilizumab
- Azathioprine
- Methotrexate
- Cyclosporine
- Leflunomide

11. Have you ever had any of the following medication (may be multiple):

- Rituximab
- Abatacept
- Adalimumab
- Etanercept
- Infliximab
- Basiliximab
- Cyclophosphamide

12. Have you ever had chemotherapy for cancer? (yes/no)

13. Have you ever had immunosuppressive medication not listed in the above questions? (yes/no). If yes:

- Free text

For peer review only

Reporting checklist for cross sectional study.

Based on the STROBE cross sectional guidelines.

Instructions to authors

Complete this checklist by entering the page numbers from your manuscript where readers will find each of the items listed below.

Your article may not currently address all the items on the checklist. Please modify your text to include the missing information. If you are certain that an item does not apply, please write "n/a" and provide a short explanation.

Upload your completed checklist as an extra file when you submit to a journal.

In your methods section, say that you used the STROBE cross sectionalreporting guidelines, and cite them as:

von Elm E, Altman DG, Egger M, Pocock SJ, Gotzsche PC, Vandenbroucke JP. The Strengthening the Reporting of Observational Studies in Epidemiology (STROBE) Statement: guidelines for reporting observational studies.

		Reporting Item	Page Number
**Title and abstract**			
Title	#1a	Indicate the study's design with a commonly used term in the title or the abstract	1

For peer review only

Abstract	#1b	Provide in the abstract an informative and balanced summary of what was done and what was found	3-4
**Introduction**			
Background / rationale	#2	Explain the scientific background and rationale for the investigation being reported	5
Objectives	#3	State specific objectives, including any prespecified hypotheses	5
**Methods**			
Study design	#4	Present key elements of study design early in the paper	6
Setting	#5	Describe the setting, locations, and relevant dates, including periods of recruitment, exposure, follow-up, and data collection	6
Eligibility criteria	#6a	Give the eligibility criteria, and the sources and methods of selection of participants.	7
	#7	Clearly define all outcomes, exposures, predictors, potential confounders, and effect modifiers. Give diagnostic criteria, if applicable	7
Data sources / measurement	#8	For each variable of interest give sources of data and details of methods of assessment (measurement). Describe comparability of assessment methods if there is more than one group. Give information separately for for exposed and unexposed groups if applicable.	7

For peer review only

Bias	#9	Describe any efforts to address potential sources of bias	7
Study size	#10	Explain how the study size was arrived at	8
Quantitative variables	#11	Explain how quantitative variables were handled in the analyses. If applicable, describe which groupings were chosen, and why	7-8
Statistical methods	#12a	Describe all statistical methods, including those used to control for confounding	7-8
Statistical methods	#12b	Describe any methods used to examine subgroups and interactions	7-8
Statistical methods	#12c	Explain how missing data were addressed	-
Statistical methods	#12d	If applicable, describe analytical methods taking account of sampling strategy	-
Statistical methods	#12e	Describe any sensitivity analyses	-
**Results**			
Participants	#13a	Report numbers of individuals at each stage of study—eg numbers potentially eligible, examined for eligibility, confirmed eligible, included in the study, completing follow-up, and analysed. Give information separately for for exposed and unexposed groups if applicable.	8
Participants	#13b	Give reasons for non-participation at each stage	-

For peer review only

Participants	#13c	Consider use of a flow diagram	
Descriptive data	#14a	Give characteristics of study participants (eg demographic, clinical, social) and information on exposures and potential confounders. Give information separately for exposed and unexposed groups if applicable.	Page 8; Table 1
Descriptive data	#14b	Indicate number of participants with missing data for each variable of interest	Table 2
Outcome data	#15	Report numbers of outcome events or summary measures. Give information separately for exposed and unexposed groups if applicable.	Table 2
Main results	#16a	Give unadjusted estimates and, if applicable, confounder-adjusted estimates and their precision (eg, 95% confidence interval). Make clear which confounders were adjusted for and why they were included	8-10
Main results	#16b	Report category boundaries when continuous variables were categorized	-
Main results	#16c	If relevant, consider translating estimates of relative risk into absolute risk for a meaningful time period	-
Other analyses	#17	Report other analyses done—e.g., analyses of subgroups and interactions, and sensitivity analyses	10
**Discussion**			
Key results	#18	Summarise key results with reference to study objectives	11

For peer review only

Limitations	#19	Discuss limitations of the study, taking into account sources of potential bias or imprecision. Discuss both direction and magnitude of any potential bias.	13
Interpretation	#20	Give a cautious overall interpretation considering objectives, limitations, multiplicity of analyses, results from similar studies, and other relevant evidence.	13
Generalisability	#21	Discuss the generalisability (external validity) of the study results	13
**Other Information**			
Funding	#22	Give the source of funding and the role of the funders for the present study and, if applicable, for the original study on which the present article is based	14

None The STROBE checklist is distributed under the terms of the Creative Commons Attribution License CC-BY. This checklist can be completed online using https://www.goodreports.org/, a tool made by the EQUATOR Network in collaboration with Penelope.ai

For peer review only
